# Maresin 1 promotes nerve regeneration and alleviates neuropathic pain after nerve injury

**DOI:** 10.1186/s12974-022-02405-1

**Published:** 2022-02-02

**Authors:** Jinhuan Wei, Wenfeng Su, Yayu Zhao, Zhongya Wei, Yuchen Hua, Peng Xue, Xiang Zhu, Ying Chen, Gang Chen

**Affiliations:** 1 Center for Basic Medical Research, Medical School of Nantong University, Nantong, 226001 Jiangsu China; 2 Key Laboratory of Neuroregeneration of Jiangsu and the Ministry of Education, Co-Innovation Center of Neuroregeneration, Nantong University, Nantong, 226001 Jiangsu China; 3 Department of Anesthesiology, Affiliated Hospital of Nantong University, Nantong, China; 4 Department of Histology and Embryology, Medical School of Nantong University, Nantong, 226001 Jiangsu China

**Keywords:** Maresin 1, Inflammation, Nerve regeneration, Neurological recovery, Neuropathic pain, Never injury

## Abstract

**Background:**

Peripheral nerve injury (PNI) is a public health concern that results in sensory and motor disorders as well as neuropathic pain and secondary lesions. Currently, effective treatments for PNI are still limited. For example, while nerve growth factor (NGF) is widely used in the treatment of PNI to promote nerve regeneration, it also induces pain. Maresin 1 (MaR1) is an anti-inflammatory and proresolving mediator that has the potential to regenerate tissue. We determined whether MaR1 is able to promote nerve regeneration as well as alleviating neuropathic pain, and to be considered as a putative therapeutic agent for treating PNI.

**Methods:**

PNI models were constructed with 8-week-old adult male ICR mice and treated with NGF, MaR1 or saline by local application, intrathecal injection or intraplantar injection. Behavioral analysis and muscle atrophy test were assessed after treatment. Immunofluorescence assay was performed to examine the expression of ATF-3, GFAP, IBA1, and NF200. The expression transcript levels of inflammatory factors IL1β, IL-6, and TNF-α were detected by quantitative real-time RT-PCR. AKT, ERK, mTOR, PI3K, phosphorylated AKT, phosphorylated ERK, phosphorylated mTOR, and phosphorylated PI3K levels were examined by western blot analysis. Whole-cell patch-clamp recordings were executed to detect transient receptor potential vanilloid 1 (TRPV1) currents.

**Results:**

MaR1 demonstrated a more robust ability to promote sensory and motor function recovery in mice after sciatic nerve crush injury than NGF. Immunohistochemistry analyses showed that the administration of MaR1 to mice with nerve crush injury reduced the number of damaged DRG neurons, promoted injured nerve regeneration and inhibited gastrocnemius muscle atrophy. Western blot analysis of ND7/23 cells cultured with MaR1 or DRG neurons collected from MaR1 treated mice revealed that MaR1 regulated neurite outgrowth through the PI3K–AKT–mTOR signaling pathway. Moreover, MaR1 dose-dependently attenuated the mechanical allodynia and thermal hyperalgesia induced by nerve injury. Consistent with the analgesic effect, MaR1 inhibited capsaicin-elicited TRPV1 currents, repressed the nerve injury-induced activation of spinal microglia and astrocytes and reduced the production of proinflammatory cytokines in the spinal cord dorsal horn in PNI mice.

**Conclusions:**

Application of MaR1 to PNI mice significantly promoted nerve regeneration and alleviated neuropathic pain, suggesting that MaR1 is a promising therapeutic agent for PNI.

**Supplementary Information:**

The online version contains supplementary material available at 10.1186/s12974-022-02405-1.

## Background

Peripheral nerve injury (PNI) is a common clinical disease that is usually caused by sudden crushing, strong external force, ischemic injuries, transection, or other iatrogenic injuries. Patients with PNI are somewhat prohibited by sensory and motor disorders and endure neuropathic pain and its secondary lesions [1–3]. Various experimental and clinical strategies, such as nerve grafts, nerve transfers, nerve conduits, cell-based therapies, and gene therapy, have been implemented to improve neuron survival, improve axon regeneration and target reinnervation [1, 2]. The use of appropriate cytokines to protect damaged neurons and promote axonal regeneration has always been an important strategy in the study of nerve regeneration. Among cytokines, nerve growth factor (NGF) is well known to play key roles in neuronal survival, growth and maintenance in response to injury [4]. However, researchers studying the effects of NGF on PNI have not paid much attention to its side effect of inducing neuropathic pain [5]. For the last two decades, an increasing number of studies have focused on anti-NGF therapy [6]. Since NGF plays two roles in the treatment of PNI, improving nerve regeneration but accelerating neuropathic pain, the discovery of novel cytokine(s) or drug molecular(s) that can protect injured neurons, promote axonal regeneration, and inhibit neuropathic pain in the treatment of PNI is a very urgent and important task.

Maresin 1 (MaR1), an anti-inflammatory and proresolving mediator, is a dioxygenation product produced by human macrophages that was discovered and named by Serhan et al. [7]. It has shown promising value in the treatment of airway inflammation, pneumonia, colitis, delayed wound healing and diabetes complications and has been shown to ameliorate pain hypersensitivity and provide neuronal protection [8–12]. In planaria, MaR1 promoted the speed of head reappearance and increased the rate of surgical regeneration [13]; in rats and mice, MaR1 promoted bone regeneration [14, 15]. These results prompted us to investigate whether MaR1 can simultaneously exert anti-inflammatory and analgesic effects and accelerate nerve regeneration after nervous system injury.

In this report, we examined whether MaR1 could promote nerve regeneration and neurological functional recovery and alleviate neuropathic pain after nerve injury and compared its effects with those of NGF.

## Methods

### Mice and surgery

Mouse experiments were performed according to guidelines established by the Institutional Animal Care and Use Committee of Nantong University and were conducted following the ARRIVE guidelines [16, 17]. A prior sample size calculation was executed using degree of freedom (*E* value) [18] and sample sizes were estimated based on our previous studies for similar types of behavioral, biochemical, and electrophysiological analyses [19, 20]. The ICR mice used for the experiments were obtained from the Laboratory Animal Center of Nantong University. Eight-week-old adult male ICR mice (25–30 g) were used to construct the PNI models, including a sciatic nerve crush model and a sciatic nerve chronic constriction injury model (CCI). Primary dorsal root ganglion (DRG) neurons were isolated from newborn ICR mice (postnatal day 0–1) or adult male ICR mice. Total 235 mice were killed in the current study, including 163 adult male mice (8–10 weeks old) and 72 newborn ICR mice (postnatal day 0–1). The sciatic nerve crush injury model was established according to our previous report [21] and the schematic overview of the experimental timeline is shown in Additional file 1: Figure S1. Briefly, after anaesthetization with isoflurane, the left sciatic nerve of the mouse was squeezed with no. 5 jeweler’s forceps for 20 s. In some cases, a diluted fluorescent dye (0.5 μl of Vybrant CM-Dil in 20 μl of PBS) was injected into the hind paw on the injured side at 7 days after nerve crushing, and L5 DRG sections were examined 7 days later. The CCI model was produced by placing three ligatures (7-0 Prolene, 1-mm intervals) around the left sciatic nerve proximal to the trifurcation. The ligatures were softly tied until a short flick of the ipsilateral hind limb occurred [22]. The mice in the sham group were subjected to the surgery described above but not to nerve injury. The mice were separated into groups (5 mice per cage) and housed under standard conditions (25 ± 1 °C, 12-h light/dark cycle, ad libitum access to food and water).

### Reagents and administration

Capsaicin (catalog: 404-86-4) and NGF-7S (catalog: N0513, 130 kDa) were purchased from Sigma-Aldrich. Maresin 1 (7R, 14S-dihydroxy-docosa-4Z, 8E, 10E, 12Z, 16Z, 19Z-hexaenoic acid) was purchased from Cayman Chemical Company (catalog: 1268720-28-0). Vybrant CM-Dil Cell-Labeling Solution was purchased from Invitrogen (catalog: V22885). A sterilized hemostatic gelatin sponge containing 500 ng of MaR1 or saline (control) was immediately applied locally to the injured nerve after crushing, and the wound was then closed. Drugs in 20 μl of PBS were intraplantarly injected using a Hamilton microsyringe with a 30-G needle. The spinal cord was punctured with a 30-gauge needle between the L5 and L6 levels for intrathecal drug delivery.

### Cell culture

DRG neurons were harvested from newborn ICR mice and then subjected to explant culture or dissociated culture as previously described [23]. A widely applied neuronal ND cell line (ND7/23) that is a hybrid between dorsal root neurons and neuroblastoma N18 Tg2, exhibiting sensory neuron-like properties was used in this work [24–26]. In brief, ND7/23 rat DRG/mouse neuroblastoma hybrid cells were obtained from Sigma-Aldrich and maintained in DMEM supplemented with 10% FBS, 2 mM l-glutamate and 10% penicillin/streptomycin.

### Whole-cell patch-clamp recordings in dissociated mouse DRG neurons

As we described previously [20], whole-cell patch-clamp recordings in dissociated DRG neurons (small size, < 25 mm) harvested from 4- to 6-week-old mice were performed at room temperature using an Axopatch-200B amplifier (Axon Instruments, USA). The patch pipettes were pulled from borosilicate capillaries (Chase Scientific Glass Inc., Rockwood, TN, USA), and their resistance when filled with the pipette solution (in mM: 126K-gluconate, 10 NaCl, 1 MgCl_2_, 10 EGTA, 2 NaATP, and 0.1 MgGTP, adjusted to pH 7.3 with KOH) was 4–5 MΩ. Whole-cell recordings were performed in an extracellular solution (in mM): 140 NaCl, 5 KCl, 2 CaCl_2_, 1 MgCl_2_, 10 HEPES, 10 glucose, adjusted to pH 7.4 NaOH. A voltage clamp was applied at a holding membrane potential of − 70 mV to record the inward currents, and the recording chamber (300 µl) was superfused continuously (3–4 ml/min). We compensated for series resistance (> 80%) and performed leak subtraction. The data were low-pass filtered at 2 kHz and sampled at 10 kHz, and pClamp10 (Axon Instruments) software was used for the experiments and data analysis.

### Behavioral analysis

A total of four double-blind behavioral tests were used. The double-blind behavioral tests were performed like this: the anonymous reagents (saline, NGF or Maresin 1) were administered to the mice by one researcher, and the behavior analysis were performed and recorded by another observer who has no information about the experimental design. (1) Walking track (footprint) analysis was used to analyze basic motor functions, and the sciatic function index (SFI) was calculated as previously reported [21]. In brief, the plantar surface of each mouse’s hind paw was smeared with ink, and the mouse was allowed to walk in a straight path on white paper. (2) The rotarod test (LE8200, RWD Life Science Co., Ltd) was used to test complex motor functions as previously reported [27]. Briefly, mice were tested three times at 10-min intervals, and the time spent on the rod was recorded and averaged. During the test, the speed was increased from 2 to 20 rpm over a 3-min period. (3) The von Frey test was used to test mechanical sensitivity as previously reported [20]. Briefly, the mice were placed in a box on an elevated metal mesh floor and stimulated with a series of von Frey filaments of logarithmically increasing stiffness (0.02–2.56 gf; Stoelting Company) on their hind paws. The 50% paw withdrawal threshold was determined by Dixon’s up–down method. (4) Using a Hargreaves radiant heat apparatus (IITC Life Science) as previously reported [27], we tested the thermal sensitivity. The basal paw withdrawal latency was adjusted to 9 to 12 s with a cutoff of 20 s to avoid tissue damage.

### Immunofluorescence assay

As we described previously [28], the mice were deeply anesthetized with isoflurane, and their ascending aortas were perfused first with PBS and then with 4% paraformaldehyde. After perfusion, the L4–L6 spinal cord segments were collected and then postfixed overnight. The spinal cord sections were sliced at a thickness of 30 µm (free-floating) on a cryostat and processed for immunohistochemistry analysis. The sections were first blocked with 2% goat serum at room temperature for 1 h and then incubated at 4 °C overnight with the following primary antibodies: anti-ATF-3 (rabbit, 1:1000, Santa Cruz Biotechnology Inc.), anti-GFAP (mouse, 1:1000, EMD Millipore), anti-IBA1 (rabbit, 1:1000; Wako Chemicals Inc., USA) and anti-NF200 (mouse, 1:1000, Sigma, catalog: N0142). After washing, the sections were incubated at room temperature for 2 h with the following secondary antibodies (1:400, Jackson ImmunoResearch): Cy3-donkey anti-rabbit (catalog: 711-165-152) and FITC-donkey anti-mouse (catalog: 715-095-150). The stained sections were observed and photographed with a Leica fluorescence microscope.

### Muscle atrophy test

After the administration of MaR1 for 9 days after CCI, the gastrocnemius muscles from both hind legs were separated for imaging and weight measurements. The muscle size and weight were used to assess muscle atrophy.

### Quantitative real-time RT-PCR

Total RNA was collected from ipsilateral and contralateral spinal dorsal horn tissues using TRIzol reagent (Life Technologies, USA), and 1 µg of RNA was reverse-transcribed using the PrimeScript RT reagent kit (Takara, Dalian, China). Gene-specific mRNA analyses were performed using the MiniOpticon Real-Time PCR system (BioRad), and the mRNA expression levels were calculated using the 2−ΔΔCt method. The specific primers, including those for the GAPDH control, were synthesized by Thermo Fisher Scientific, and the sequences were as follows: IL1β (forward TACATCAGCACCTCACAAGC, reverse AGAAACAGTCCAGCCCATACT), IL-6 (forward TCCATCCAGTTGCCTTCTTGG, reverse CCACGATTTCCCAGAGAACATG), TNF-α (forward CCCCAAAGGGATGAGAAGTT, reverse CACTTGGTGGTTTGCTACGA) and GAPDH (forward TTGATGGCAACAATCTCCAC, reverse CGTCCCGTAGACAAAATGGT).

### Western blot analysis

Total proteins were extracted from ND7/23 cells or DRG neurons isolated from mice with RIPA lysis buffer (Beyotime, Shanghai, China) containing a protease inhibitor cocktail and phosphate inhibitors (Roche Molecular Biochemicals, Inc. Mannheim, Germany). The proteins were separated on 8% or 10% SDS-PAGE gels and electrophoretically transferred onto PVDF membranes. The membranes were blocked with 5% nonfat milk for 1–2 h and then incubated with antibodies against phosphorylated AKT (p-AKT) (1:1000, rabbit, Cell Signaling, catalog: 9271), AKT (1:1000, rabbit, Cell Signaling, catalog: 9272), phosphorylated ERK (p-ERK) (1:1000, rabbit, Cell Signaling, catalog: 9101), total ERK (1:1000, rabbit, Cell Signaling, catalog: 9102), phosphorylated mTOR (p-mTOR) (1:1000, rabbit, Cell Signaling, catalog: 2971), mTOR (1:1000, rabbit, Cell Signaling, catalog: 2972), phosphorylated PI3K (p-PI3K) (1:1000, rabbit, catalog: 4228), PI3K (1:1000, rabbit, Cell Signaling, catalog: 4292), and GAPDH (1:10,000, mouse, Proteintech, catalog: 60004-1-lg) overnight at 4 °C. The next day, the membranes were incubated with the corresponding secondary antibody at room temperature for 2 h. Bands were detected using PierceTM ECL western blotting substrate (Thermo, USA), and the results were analyzed by ImageJ software.

### Statistical analyses

All data are expressed as the mean ± SD, as indicated in the figure captions. Student’s *t*-test (two groups) or ANOVA (one-way and two-way) test or Bonferroni post hoc test was used to compare the differences between groups, followed by Bonferroni’s test. The criterion for statistical significance was *p* < 0.05. Statistical report and *t*-test comparison are shown in Additional file 3, 4; Supplementary Table 1 and Table 2, respectively.

## Results

### Sciatic nerve crush injury caused motor dysfunction and neuropathic pain in mice

**Fig. 1:**
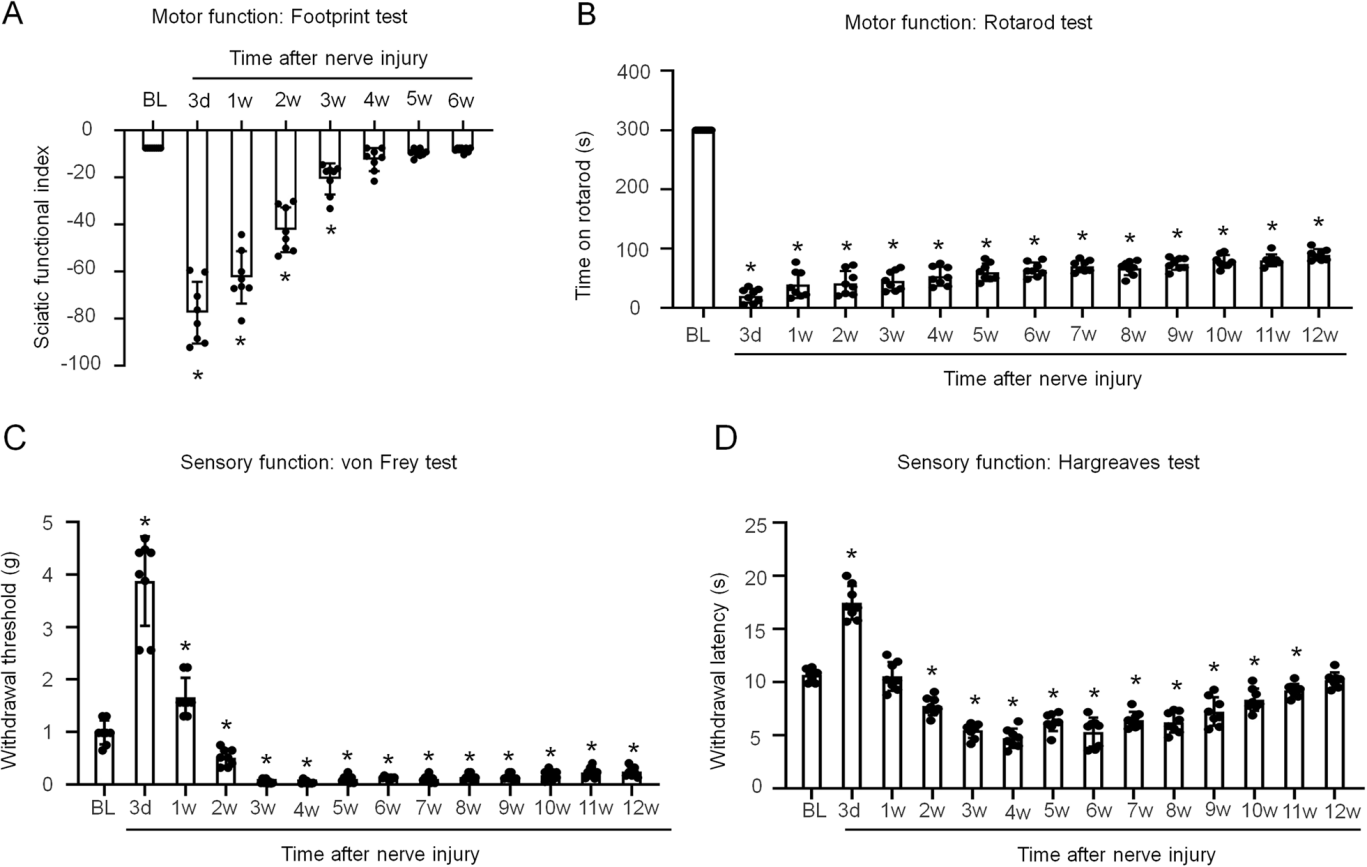
Motor and sensory dysfunction and neuropathic pain in mice with sciatic nerve crush injury. The motor functions of mice with sciatic nerve crush injury were measured by footprint (**A**) and rotarod (**B**) tests. The sensory functions of mice with sciatic nerve crush injury were measured by the von Frey (**C**) and Hargreaves (**D**) tests. The data are presented as the mean ± SD, *n* = 8 mice in each group, *p < 0.05 versus the baseline (BL) group, one-way ANOVA test

We aimed to study both nerve regeneration and neuropathic pain in one model. Therefore, we established a sciatic nerve crush injury model in mice and examined motor and sensory functions and pain behavior after nerve injury. We first used the SFI evaluation to assess the motor function recovery of the affected hindlimb. The footprint test showed that the mice developed severe motor dysfunction at 3 days after sciatic nerve injury, and the recovery took almost 4 weeks (Fig. 1A). Next, we performed a rotarod test to evaluate the fine motor functions of the mice after nerve crushing. Severely impaired fine motor functions were observed after nerve injury, and recovery took 12 weeks (Fig. 1B). This result differed from that of the footprint test, which showed a full recovery of basic motor function after 4 weeks. Moreover, we investigated pain behavior by the von Frey and Hargreaves tests. In the 1st week, the mice with nerve injury showed a higher mechanical withdrawal threshold than normal mice, and pain sensitivity was high during the initial recovery period, peaking in the 3rd week but lasting for more than 12 weeks (Fig. 1C). The Hargreaves test results showed that the paw withdrawal latencies of nerve-injured mice recovered to normal levels after 11 weeks (Fig. 1D). Therefore, in this model, both motor and sensory functions were damaged to some degree, and pain behaviors were obvious.

**Fig. 2:**
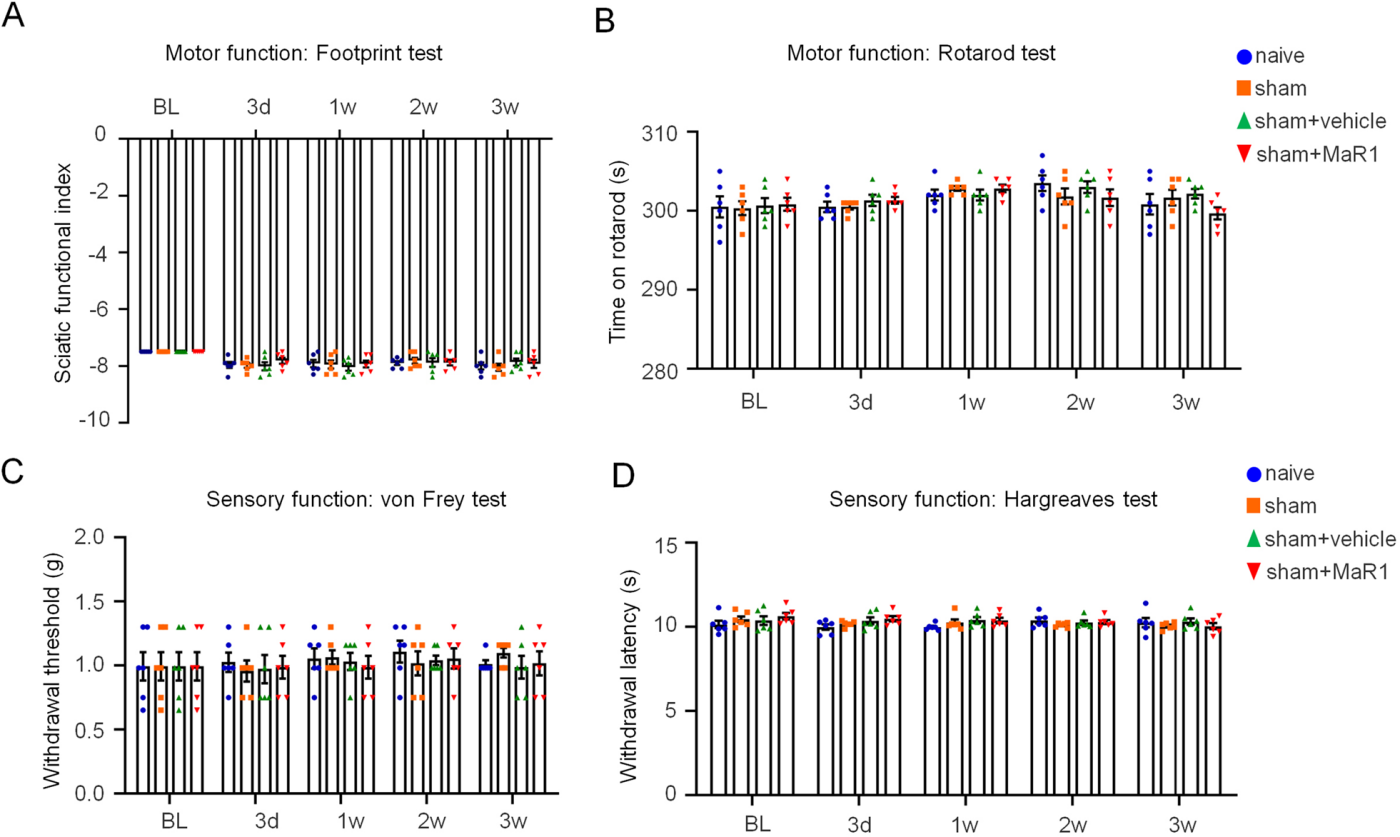
Motor and sensory functions were not affected in control groups. Naïve group, sham group, sham + vehicle group and sham + MaR1 group were used as the control groups in this study. The motor functions of mice with sciatic nerve crush injury were measured by footprint (**A**) and rotarod (**B**) tests. The sensory functions of mice with sciatic nerve crush injury were measured by the von Frey (**C**) and Hargreaves (**D**) tests. The data are presented as the mean ± SD, *n* = 6 mice in each group, one-way ANOVA test

Besides, we executed a 3-week-period experiment to monitor motor functions and sensory functions of four groups: naïve group (normal mice), sham control (surgery but no crush), sham + MaR1 group, sham + vehicle group, and found there was no significant change among four groups, which suggesting that MaR1 has no detectable negative effect on healthy mice (Fig. 2).

### MaR1 stimulated axon regeneration

**Fig. 3:**
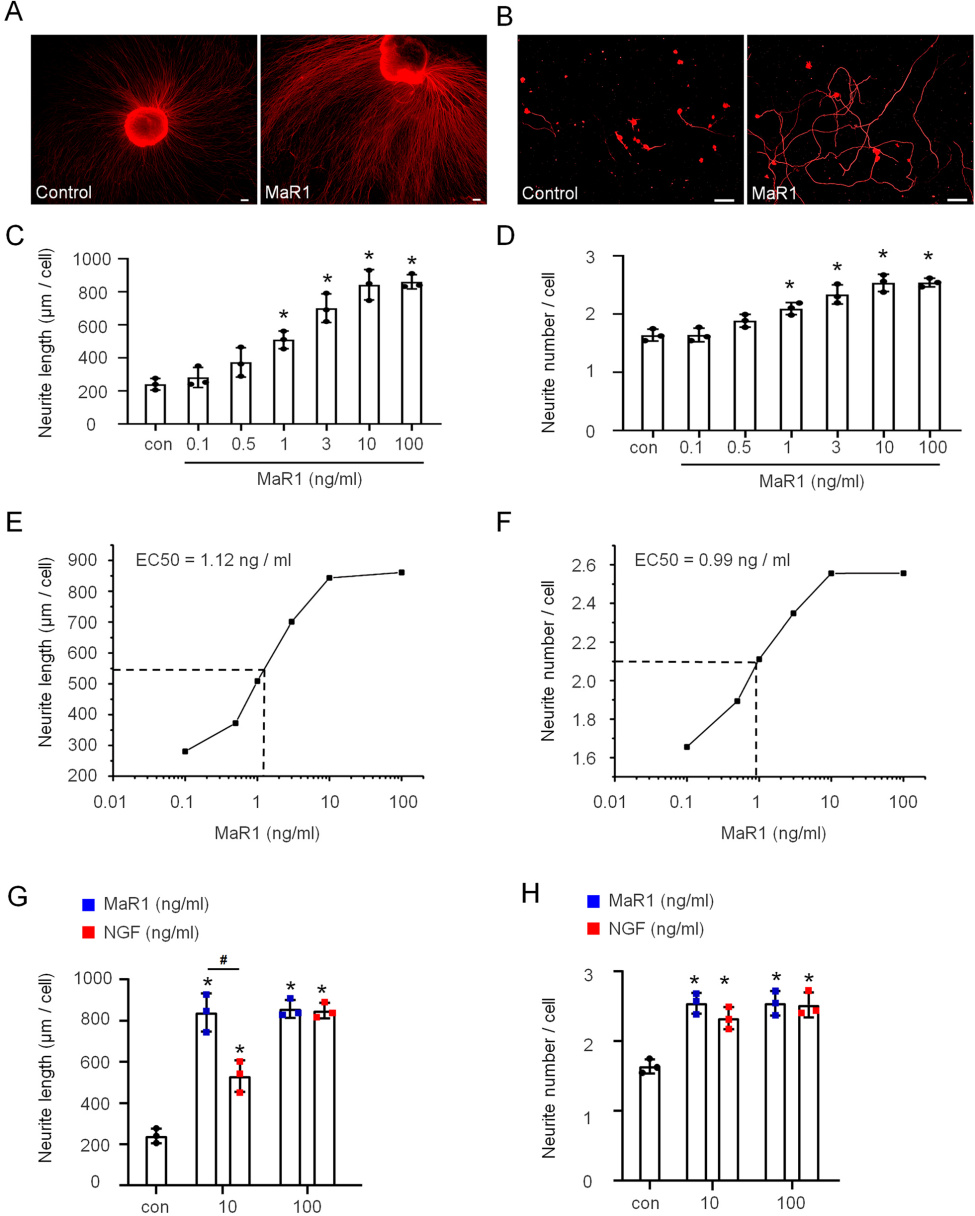
MaR1 dose-dependently promoted axon regeneration in vitro. MaR1 stimulated neurite outgrowth in DRG neurons grown in explant and dissociated cultures in vitro. Neurofilament staining was employed to examine the outgrowth of neurites in the DRG. **A** DRG neurons grown in explant cultures for 5 days and treated with (right) and without (left) MaR1. Scale bars = 100 μm. **B** DRG neurons grown in dissociated cultures for 36 h and treated with (right) and without (left) MaR1. Scale bars = 100 μm. **C**, **D** MaR1 increased the neurite length and number in a dose-dependent manner. DRG neurons cultured with MaR1 for 36 h. **E**, **F** The EC50 values at which MaR1 increased the neurite length and number were measured. **G**, **H** Comparison of the effects of MaR1 and NGF at the same concentration on increasing the neurite length and number. MaR1 and NGF were added in the culture medium. The data are presented as the mean ± SD, *p < 0.05 versus the control group; ^#^*p* < 0.05 versus the NGF group, Student’s *t*-test and one-way ANOVA

A previous study reported that MaR1 potentially stimulated tissue regeneration in planaria [13]. And we herein investigated whether MaR1 could promote nerve regeneration in injured mice. To answer this question, we harvested newborn mouse DRGs and then cultured them with MaR1. After the administration of MaR1 (10 ng/ml) for 5 days, the DRG explants showed more extending nerve fiber bundles and more neurons than those not treated with MaR1 (Fig. 3A). Similar results were observed when MaR1 was applied to dissociated DRG cell cultures for 36 h. The DRGs in the MaR1 group displayed substantially more and longer neurites than those in the control group without MaR1 treatment (Fig. 3B). Therefore, MaR1 directly influences neurite outgrowth in DRG cells from both dissociated and explant cultures.

Most neurotrophic factors promote neurite outgrowth in a dose-dependent manner [29]. To determine the optimal dosage of MaR1 for further study, we cultured DRG cells with the agent at a series of concentrations (0.1, 0.5, 1, 3, 10, 100 ng/ml) for 36 h. MaR1 (1, 3, 10, 100 ng/ml) significantly increased the length and number of neurites (Fig. 3C, D, *p* < 0.05) in a dose-dependent manner compared with those in the control group. However, the results of the 100 ng/ml MaR1 group were not significantly different from those of the 10 ng/ml MaR1 group. In addition, we assessed the EC50 values (concentration needed to achieve 50% of the maximal effect) of MaR1 and found that the values were 1.12 ng/ml and 0.99 ng/ml for promoting the neurite length and number, respectively (Fig. 3E, F). Moreover, we compared the effects of NGF and MaR1 at the same dosage on neurite outgrowth. NGF promoted the length and number of neurites also in a dose-dependent manner with an optimal threshold effect occurring at 100 ng/ml (Additional file 2: Figure S2). Compared with EC50 for MaR1 was around 1 ng/ml to promote neurite length and number, the EC50 for NGF was 7–9 ng/ml. More interestingly, 10 ng/ml MaR1 had a much stronger ability to increase neurite length than NGF at the same concentration (Fig. 3G), but their abilities to increase neurite numbers were not significantly different (Fig. 3H). Taken together, our results suggest that MaR1 promotes neurite outgrowth in DRG cells cultured in vitro at a lower dosage than that required for NGF.

### MaR1 reduced DRG neuronal damage and promoted nerve path reconstruction after nerve injury

**Fig. 4:**
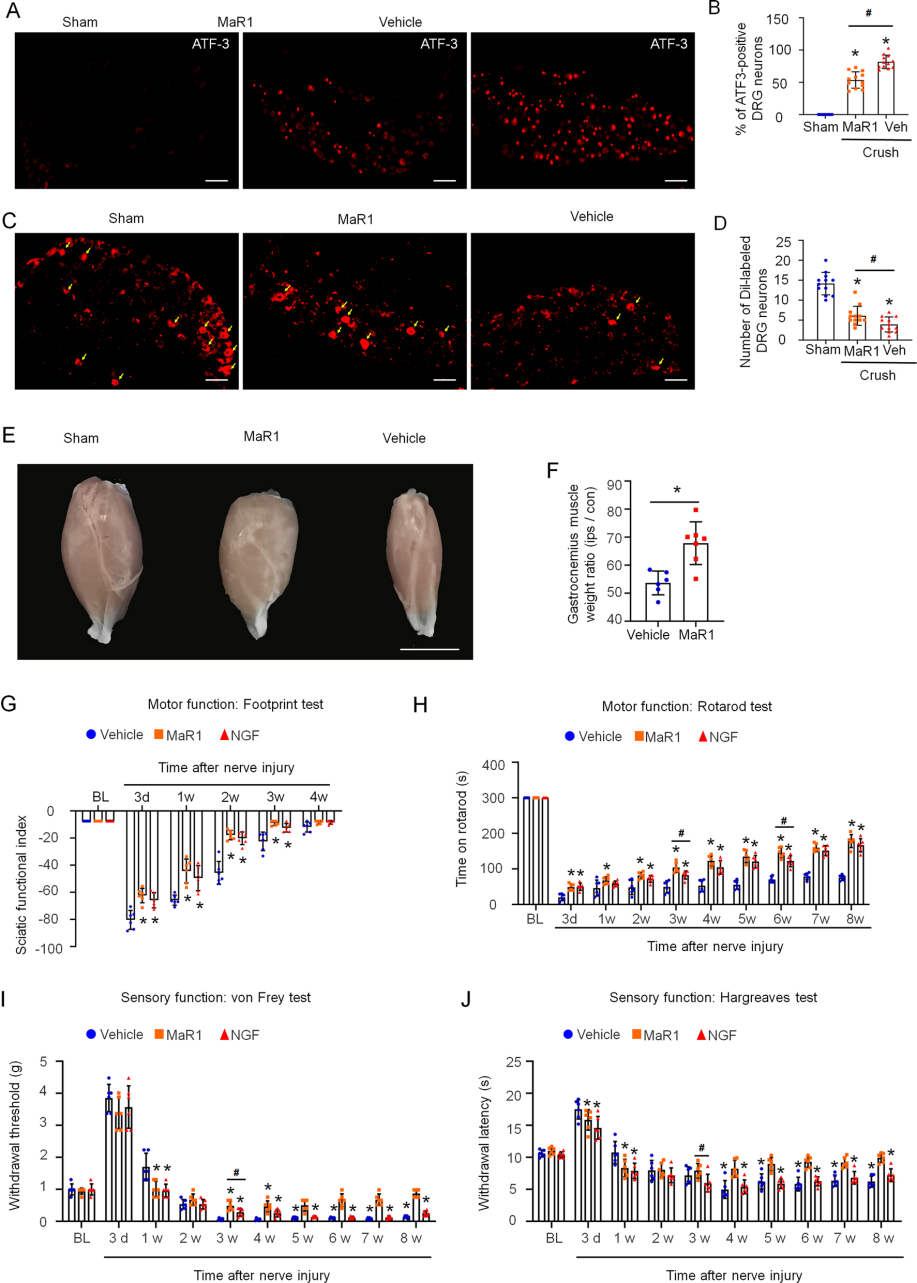
MaR1 protected against DRG neuronal damage and improved functional neurological recovery. Immunohistochemical analysis of ATF3 was used to determine the number of DRG neurons after 7 days of MaR1 or saline (vehicle) treatment (**A**, **B**). Neuronal regeneration was detected by the Dil labeling of DRG neurons after 9 days of MaR1 or vehicle administration (**C**, **D**). MaR1 (500 ng) or saline (vehicle) was administered using the sterile gelatin sponge to the injured region, scale bars = 50 μm. The data are presented as the mean ± SD, *p < 0.05 versus the sham group; ^#^*p* < 0.05 versus the vehicle group, one-way ANOVA. **E**, **F** The size and weight of the gastrocnemius muscle were measured after 9 days treated with MaR1 or saline. Scale bars = 5 mm. The data are presented as the mean ± SD, *p < 0.05 versus the vehicle group, Student’s *t*-test. **G**–**J** The local application of MaR1 (500 ng in a sterile gelatin sponge) or NGF (500 ng in sterile gelatin sponge) to the injured nerve promoted motor and sensory function recovery, but MaR1 was more effective. The data are presented as the mean ± SD, *n* = 6 mice in each group, *p < 0.05 versus the BL group, ^#^*p* < 0.05 versus the NGF group, ^$^*p* < 0.05 versus both the BL and NGF groups, two-way ANOVA and Bonferroni post hoc test

Next, we examined whether MaR1 could attenuate DRG neuronal damage induced by nerve crush injury in mice. After the surgery, the damaged nerves of mice in the experimental group were covered with a sterilized hemostatic gelatin sponge containing 500 ng of MaR1, while those of mice in the control group were covered with the saline vehicle. Immunohistochemical analysis of ATF3 showed that MaR1 significantly reduced the number of damaged DRG neurons after 7 days of treatment (Fig. 4A, B). Our results suggested that MaR1 not only prohibited more DRG neuronal damage after nerve crush injury but also promoted the recovery of motor nerves and nerve path reconstruction. To confirm this hypothesis, we observed the regeneration of DRG neurons by intraplantarly injecting Dil, a fluorescent tracer dye, at 7 days after nerve injury. Two days later, more neurons were dyed with Dil in the MaR1 group than in the vehicle control group (Fig. 4C, D), which indicated that MaR1 promoted nerve path reconstruction. Because muscle atrophy is a common phenomenon after PNI, we isolated the gastrocnemius muscles from mice treated with MaR1 or saline at 9 days after surgery to evaluate the ability of MaR1 to prevent muscle atrophy. Unsurprisingly, both the size and weight of the gastrocnemius muscles in the MaR1 group were larger than those of the vehicle group (Fig. 4E, F). Taken together, these results showed that MaR1 reduced DRG neuronal damage, inhibited muscle atrophy, and promoted the regeneration of DRG neurons after nerve crush injury.

### MaR1 promoted functional recovery after nerve injury more effectively than NGF

Because MaR1 administered at a lower dosage promoted neurite outgrowth better than NGF (Fig. 3G, H), we next compared the effects of MaR1 and NGF at the same dose (500 ng) on motor function and sensory function recovery in mice with nerve crush injury. Although the basic motor functions measured by the footprint test were not significantly different between the two groups of mice treated with MaR1 and NGF separately, the complex motor function measured by the rotarod test showed that MaR1 worked more quickly and efficiently than NGF to promote motor function recovery after nerve crushing (Fig. 4G, H). Similar results were obtained in the von Frey and Hargreaves tests, as MaR1 promoted the recovery of normal mechanical and thermal sensations after nerve injury faster than NGF (Fig. 4I, J). Therefore, we suggest that MaR1 is better for PNI treatment than NGF, which requires a higher dosage and a longer treatment time.

### MaR1 alleviated nerve injury-induced neuropathic pain

**Fig. 5:**
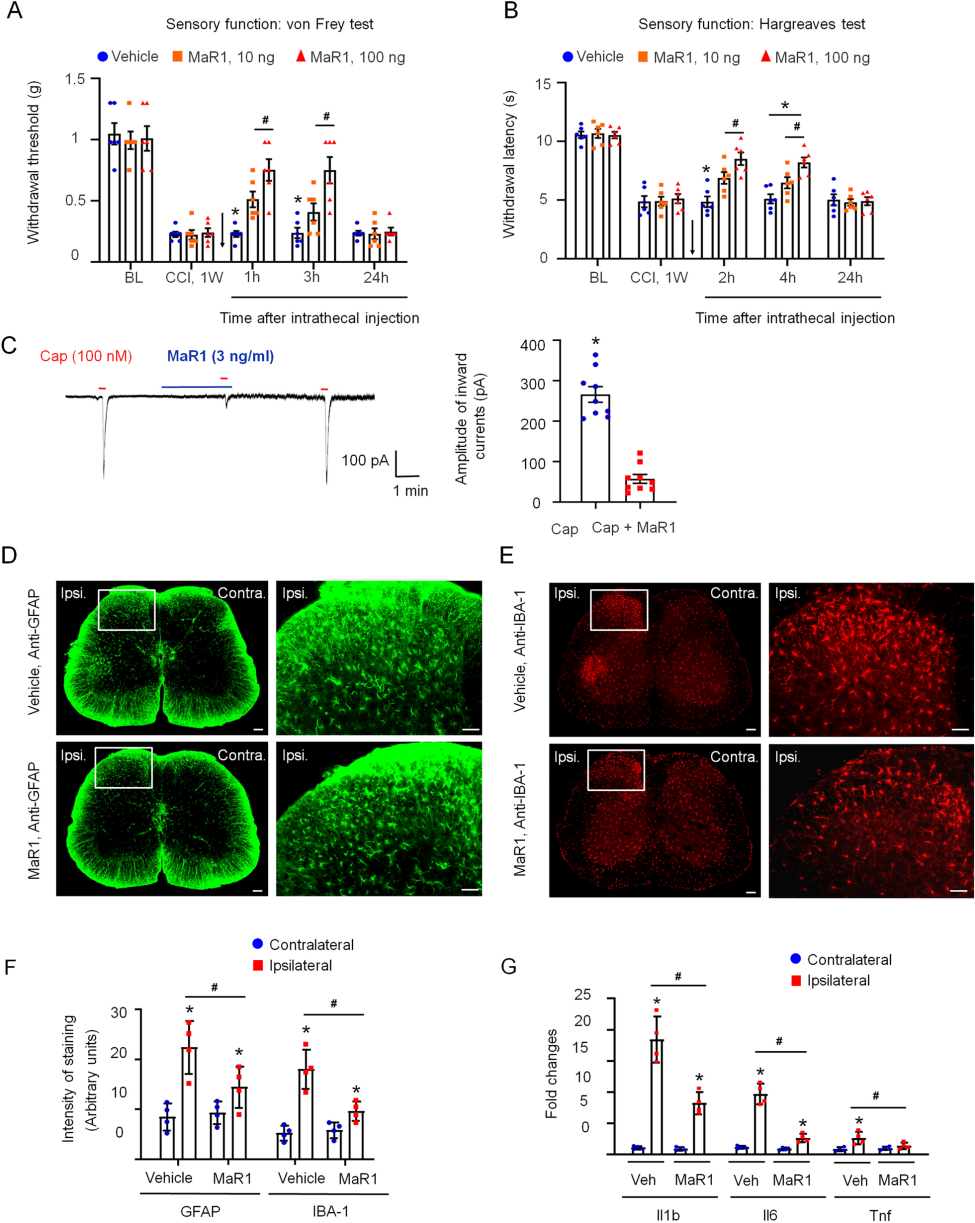
Maresin 1 alleviated nerve injury-induced neuropathic pain. Intrathecal injection of MaR1 attenuated the mechanical allodynia (**A**) and thermal hyperalgesia (**B**) induced by SCI. The data are presented as the mean ± SD, *n* = 6 mice in each group, *p < 0.05 versus the BL group; ^#^*p* < 0.05 versus the 10 ng MaR1 group, two-way ANOVA and Bonferroni post hoc test. **C** MaR1 inhibited capsaicin (CAP; 100 nM)-induced inward currents. The data are presented as the mean ± SD, *n* = 9 neurons, *p < 0.05 versus the control group, Student’s *t*-test. **D**–**F** MaR1 suppressed the activation of astrocytes (immunofluorescence staining of GFAP) and microglia (immunofluorescence staining of IBA-1) in the L4–L6 spinal dorsal horn at 7 days after sciatic nerve crush injury by double immunohistochemistry analysis. *p < 0.05 versus the contralateral group; ^#^*p* < 0.05 versus the vehicle group, Student’s *t*-test, two-way ANOVA and Bonferroni post hoc test. Intensity of staining was qualified by ImageJ. Four fluorescence positive arbitrary units were randomly selected and calculated the intensity of staining with background subtraction by analyze-measure function of ImageJ. **G** qRT-PCR indicated that MaR1 inhibited the increased mRNA expression of Il1β, Il6, and TNF-a in the ipsilateral L4–L6 region induced by sciatic nerve crush injury at 7 days after the treatment. The data are presented as the mean ± SD, *n* = 4 mice in each group. *p < 0.05 versus the contralateral group; ^#^*p* < 0.05 versus the vehicle group, Student’s *t*-test, two-way ANOVA and Bonferroni post hoc test

Next, we evaluated the effects of MaR1 on neuropathic pain in CCI mice. As shown in Fig. 5A, B the ipsilateral paw withdrawal threshold and paw withdrawal latency were dramatically decreased at 1 week after the surgery. The intrathecal injection of MaR1 (10 ng or 100 ng) rapidly reversed mechanical allodynia within 1 h, and the effect lasted for more than 3 h and was dose-dependent (Fig. 5A). Similarly, MaR1 also reduced the thermal hyperalgesia in CCI mice (Fig. 5B). The analgesic effects of MaR1 disappeared at 24 h after treatment.

Transient receptor potential vanilloid 1 (TRPV1) is a type of TRP channel that plays an important role in mediating somatic inflammatory pain after injury [30, 31], and substantial efforts have been made to develop small-molecule inhibitors of TRP channels (e.g., TRPV1 and TRPA1). A previous study suggested that MaR1 inhibits capsaicin-induced TRPV1 currents and neuronal activity in trigeminal ganglion and DRG neurons [13, 32]. Here, our patch-clamp recording results showed that 100 nM capsaicin elicited a noticeable inward current in the perfusion of mouse DRG neurons, and the inward current was almost completely inhibited by pretreatment with MaR1 (3 ng/ml) (Fig. 5C), which is consistent with previous studies reporting that MaR1 potently modulates TRPV1.

### MaR1 inhibited glial cell activation and inflammation in the spinal cord after nerve crush injury

Maintenance of the glial cell resting state is important for central nervous system homeostasis [33, 34]. The activation of glial cells by noxious stimuli and inflammation triggers an inflammatory response, thereby inducing neuropathic pain. Therefore, we evaluated the expression of the astrocytic marker GFAP and the microglial marker IBA-1 in L4–L6 spinal dorsal horn sections at 7 days after sciatic nerve crush injury by double immunohistochemistry analysis. In the vehicle group, the fluorescence intensities of GFAP and IBA-1 immunoreactivity in the spinal cord dorsal horn were markedly upregulated on the ipsilateral side compared with the contralateral side (Fig. 5D–F). In contrast, the intensities of GFAP and IBA-1 immunoreactivity on the ipsilateral side were significantly reduced in the group treated with the sterile gelatin sponge containing 500 ng of MaR1 immediately after nerve crush injury (Fig. 5D–F).

Due to their roles in inflammatory propagation and neutrophil recruitment, proinflammatory cytokines are important in the initiation and maintenance of neuropathic pain [35]. The expression levels of the inflammatory factors IL1β, IL6 and TNF-ɑ in the ipsilateral L4–L6 spinal dorsal horn were increased compared with those on the contralateral side at 7 days after the nerve crush injury as determined by qRT-PCR (Fig. 5G). However, the levels of these inflammatory factors were significantly suppressed by the local application of MaR1 to the injured nerve (Fig. 5G). These results suggest that MaR1 attenuates nerve injury-induced neuropathic pain by repressing the astrocyte and microglia activation and proinflammatory cytokine production in the spinal cord.

### MaR1 promoted nerve growth via the PI3K–AKT–mTOR signaling pathway

**Fig. 6:**
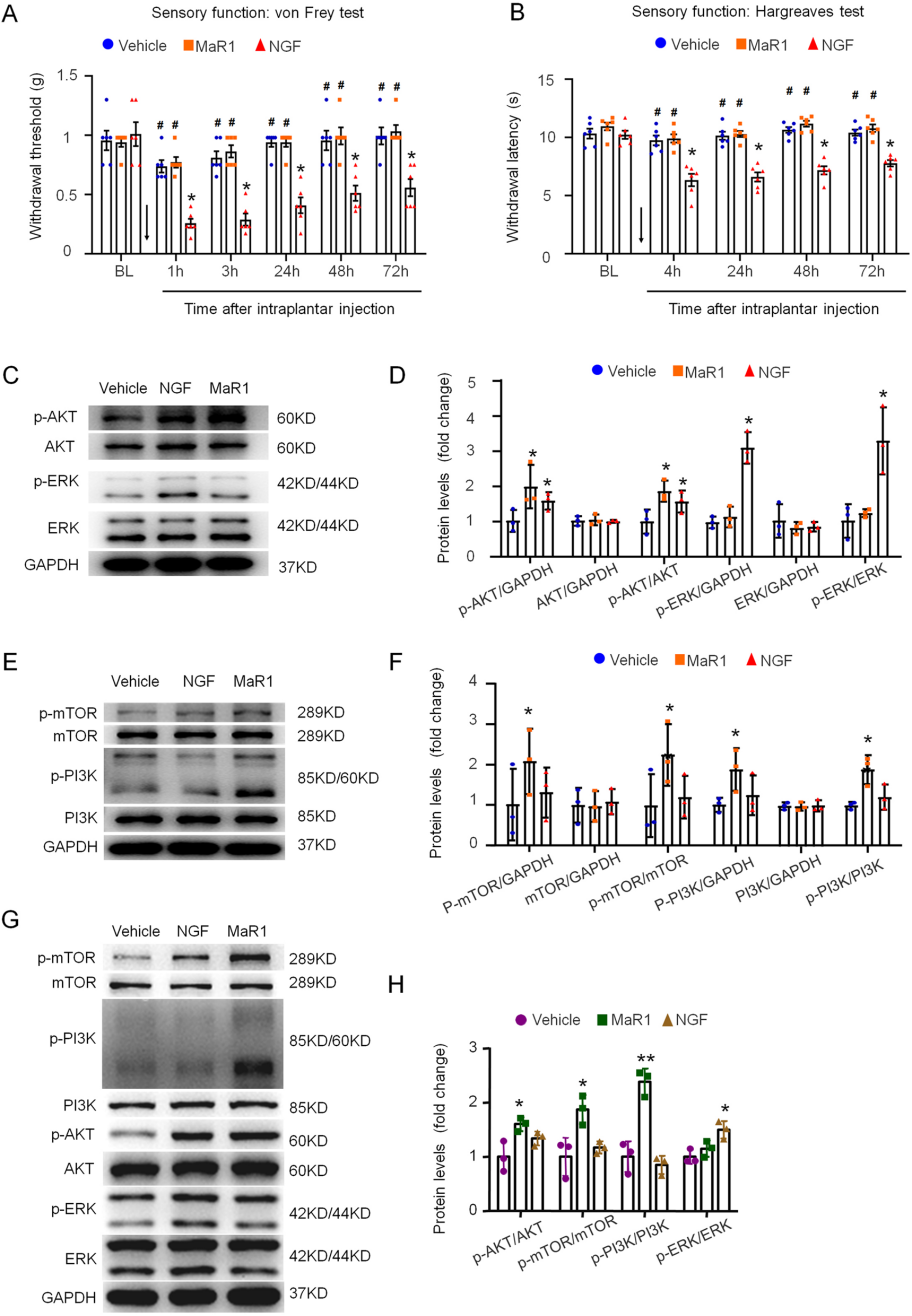
Maresin 1 regulated the PI3K–AKT–mTOR pathway. **A**, **B** Intraplantar injection of MaR1 did not affect the mechanical withdrawal threshold or paw withdrawal latency of normal mice, while NGF did. The data are presented as the mean ± SD, *n* = 6 mice in each group. *p < 0.05 MaR1 group verse the vehicle group; ^#^*p* < 0.05 NGF group verse the vehicle group, two-way ANOVA and Bonferroni post hoc test. **C**–**F** Western blot analysis indicated that the administration of MaR1 to ND7/23 cells for 60 min significantly activated the PI3K–AKT–mTOR pathway, while NGF promoted the expression of p-PI3K, p-AKT, p-mTOR and p-ERK, *p < 0.05, Student’s *t*-test and one-way ANOVA. **G**, **H** In vivo, western blot analysis of DRG neurons isolated from mice indicated that MaR1 significantly activated the PI3K–AKT–mTOR pathway, while NGF only promoted the expression of p-ERK. DRG neurons collected from the mice with MaR1 (500 ng), NGF (500 ng) or saline (vehicle) that administered using the sterile gelatin sponge to the injured region for 3 days. The data are presented as the mean ± SD, *n* = 3 mice in each group, Student’s *t*-test and one-way ANOVA test. *p < 0.05, **p < 0.01 verse vehicle group

To assess the mechanisms underlying the analgesic effect of MaR1 in PNI, we examined its influence on the basic pain thresholds of naïve mice and compared it with that of NGF. After the intraplantar injection of saline (20 μl), MaR1 (50 ng in 20 μl saline) and NGF (50 ng in 20 μl saline), we observed no significant changes in the pain thresholds between the vehicle and MaR1 groups (Fig. 6A, B). However, the pain thresholds were decreased significantly in the NGF group at all the tested time points as determined by the von Frey and Hargreaves tests (Fig. 6A, B). Thus, given the role of NGF in promoting pain, MaR1 is much better for the treatment of nerve injury.

Because the mechanisms underlying MaR1 function are complicated and relatively unknown, we investigated whether MaR1 functions via the same pathway as NGF to better understand how it regulates neuronal growth. We treated ND7/23 cells with MaR1 or NGF for 60 min, and western blot analysis revealed that phosphorylated AKT (p-AKT) was upregulated in the MaR1 and NGF groups compared with the vehicle group, while phosphorylated ERK (p-ERK) was upregulated in only the NGF group compared with the vehicle group (Fig. 6C, D). There were no detectable differences in AKT and ERK expression among all three groups (Fig. 6C, D). We next examined the effect of MaR1 on the phosphorylation of mTOR, a common downstream protein in the Akt signaling pathway. Phosphorylated mTOR (p-mTOR) levels were increased in both the MaR1 and NGF treatment groups, and the protein expression of mTOR was not significantly different among the three groups (Fig. 6E, F). The PI3K–AKT pathway is well known to mediate neuronal survival, and we next investigated the protein expression of phosphorylated PI3K (p-PI3K). Our results clearly demonstrated that MaR1 triggered p-PI3K protein expression, like NGF, did but did not affect PI3K expression (Fig. 6E, F). Moreover, western blot analysis also exhibited the upregulated levels of p-PI3K, p-AKT and p-mTOR in the DRG neurons collected from mice treated with MaR1 for 3 days compared with that from the vehicle group, while the p-ERK/ERK level was only increased in NGF treated group (Fig. 6G, H). Taken together, our results showed that MaR1 regulates neural growth through the PI3K–AKT–mTOR signaling pathway both in vitro and in vivo.

## Discussion

PNI is a common occurrence that causes motor, sensory and autonomic nervous system dysfunction as well as neuropathic pain. While surgical intervention is the main treatment for PNI, conservative and pharmacological treatments as well as cell-based therapies, gene therapies and growth factors are also popular for patients. Several growth factors have already been identified and preclinical applied for the treatment of PNI, and NGF is one of the best studied. NGF plays vital roles in promoting the growth and survival of neurons. In recent decades, an increasing number of studies have suggested that NGF antagonism can ameliorate pain and pain-related behavior [4, 36–40]. Preclinical and clinical trials have demonstrated that the direct intradermal injection of NGF into rodents and humans clearly activates and sensitizes nociceptors [41–43]. Therefore, a variety of strategies have been designed to target the NGF pathway and thereby reduce neuropathic pain. MaR1, a newly identified anti-inflammatory and proresolving mediator, could be an important reagent for conservative treatment. Here, we showed that MaR1 stimulated the DRG growth much more strongly than NGF at the same dosage (Fig. 3G, H). We also demonstrated that MaR1 protected damaged DRG neurons and promoted functional neurological recovery. In mice with sciatic nerve crush injury, MaR1 at a low dose accelerated the recovery of both motor function and sensory function, promoted neural regeneration, and reduced DRG neuronal damage (Fig. 4I, J), which suggests that the use of MaR1 for PNI treatment will ease the economic burdens on patients.

In addition, the paw withdrawal threshold and paw withdrawal latency were decreased after the injection of NGF into the plantar tissues of normal mice, while there were no changes in these parameters in the mice receiving the MaR1 injection (Fig. 6A, B). This result suggests that even though NGF plays a neuroprotective role, its side effect of inducing mechanical and thermal pain represents a significant limitation. In contrast, MaR1 was not shown to induce pain but rather inhibited CCI-induced neuropathic pain development in our study (Fig. 4I, J). Both sciatic nerve chronic constriction injury (CCI) model and crush injury model are typical models for PNI. Sciatic crush injury causes severe anatomical damage with a large number of axons fractured and is one of the most widely applied models for the study of nerve repair and regeneration [44]. Motor and sensory impairment is the primary injury after sciatic nerve crush, and neuropathic pain is the secondary pain that occurs during nerve regeneration. CCI with extrusion leads to mild injury with little axon damage, and the inflammatory response triggers pathological pain, hence provides an opportunity to mimic neuropathic injury and much more popular be used for studying neuropathic pain [45, 46]. Therefore, the effects of MaR1 on nerve regeneration were studied by local sciatic nerve administration in sciatic crush model while the effects of MaR1 on pain relieve and the expression patterns of inflammatory factors in spinal cord dorsal horn were examined in CCI model. The intrathecal injection of MaR1 into CCI model mice reduced allodynia to some extent in a dose-dependent manner (Fig. 5A. B). All of the above results strongly indicate that MaR1 should be considered a novel target for preclinical PNI treatment.

While the mechanisms of MaR1 are not well understood, those underlying NGF-induced pain have been studied extensively and might provide some clues about how MaR1 exerts its effects. Previous studies have demonstrated that NGF binds to TrkA–TrkA or TrkA–p75NTR to phosphorylate the TrkA cytoplasmic receptor and then triggers numerous second-messenger cascades (e.g., PI3K, AKT, ERK, mTOR) to affect the growth, differentiation and survival of neuronal cells [6, 47, 48]. AKT and ERK have been indicated to significantly contribute to the pathogenesis of various neurodegenerative diseases and to PNI [49, 50]. The PI3K/AKT pathway is activated by the NGF/TrkA complex and then participates in the cognitive dysfunctional pathological process [51], regulates neurotrophin retrograde axonal transportation in the nervous system [52] and determines neuronal polarity and axon growth [53, 54]. In neuronal cell lines, PI3K promotes the outgrowth and retraction of neurites [55, 56]. Since NGF is known to bind TrkA to initiate downstream signaling pathways, such as PI3K–AKT and Ras–MEK, followed by activation of the ERK or mTOR signal transduction pathway and, finally, the regulation of cytokine secretion, we aimed to examine whether MaR1 functions via a similar pathway in ND7/23 cells and DRG neurons collected from mice by western blot. Interestingly, in ND7/23 cells, the protein expression of p-AKT was improved to similar levels in the MaR1 and NGF treatment groups, while the AKT expression was not noticeably altered in either group (Fig. 6C, D); compared with the vehicle group, p-AKT/AKT level was upregulated in MaR1 treated mice but no significant change was found in NGF treated group (Fig. 6G, H). However, no matter in ND7/23 cells or DRG neurons isolated from mice, neither the p-ERK level nor the ERK level was changed by the administration of MaR1, unlike in the NGF treatment group (Fig. 6E–H). In ND7/23cells, the expression levels of AKT downstream of p-mTOR and AKT upstream of PI3K were elevated in both the MaR1 and NGF treatment groups, which indicated that like NGF, MaR1 promotes the neuronal growth process via the PI3K–AKT–mTOR pathway in vitro, while NGF also functions via the PI3K–ERK pathway. In vivo, MaR1 also participates in PI3K–AKT–mTOR pathway while NGF seems like only gets involved in ERK signaling pathway (Fig. 6G, H).

Thus far, little is known about the MaR1 receptors. Colas et al. discovered that MaR1 is a partial agonist of recombinant human leukotriene B4 receptor (BLT1), suggesting that BLT1 is a potential receptor for MaR1 [57]. Recently, human leucine-rich repeat containing G protein-coupled receptor 6 (LGR6), a plasma membrane GPCR, was screened out as a receptor for MaR1 [58]. MaR1 and LGR6 interactions in phagocytes were clearly demonstrated to play a role in resolving inflammation. Another confirmed receptor for MaR1 is retinoic acid-related orphan receptor α (RORα), which induces nonalcoholic steatohepatitis (NASH) protection through the MaR1/RORα/12-lipoxygenase (12-LOX) autoregulatory circuit [59]. RORα and LGR6 are the molecular targets for MaR1 in chronic NASH and acute sepsis, respectively. However, whether RORα or LGR6, or even both, functions as a MaR1 receptor and thereby triggers intracellular cascades to promote PNI recovery requires further study. A deeper and updated understanding of this phenomenon will likely significantly advance the chances of MaR1 being investigated in clinical trials.

Besides, in our sciatic crush injury mice the basic motor function tested by Footprint could easily revered about 1 month while complicated motor action potentials examined by Rotarod test were slightly recovered even after 3 months (Fig. 1A, B), which is consistent with our previous results that the compound muscle action potentials (CMAPs) not completely restored even after 56 days [60]. It might due to the different strategies to construct sciatic nerve crush models, such as the distinct tools, time and force for extrusion lead to different injury degrees and recovery period. Also, the different animal models cause inconsistency since we used mice instead of the widely used rats [61].

In conclusion, we provided clear evidence that MaR1 should be considered a novel analgesic agent for the treatment of neuropathic pain and as a new activator for nerve regeneration to improve functional neurological recovery after nerve crush injury. MaR1 should be considered as the mainstay treatment for PNI rather than NGF, which is expensive and has some uncertain adverse effects.

## Conclusions

Peripheral nerve injury (PNI) is a public health concern that results in sensory and motor disorders as well as neuropathic pain and secondary lesions. Currently, effective treatments for PNI are still limited. One well-known treatment is nerve growth factor (NGF), which improves nerve regeneration but accelerates neuropathic pain. New cytokine(s) or drug molecular(s) that function better than NGF are urgently needed for patients with PNI. Maresin 1 (MaR1) is an anti-inflammatory and proresolving mediator that has the potential to regenerate tissue. Our study demonstrated that the administration of MaR1 to a mouse model of PNI not only promoted neurological functional recovery by protecting damaged DRG neurons and promoting injured axonal regeneration but also alleviated neuropathic pain by inhibiting glial cell activation and the inflammatory response in the spinal cord. Therefore, our results suggest that MaR1 is a promising therapeutic agent for PNI.

## Supplementary Information

**Additional file 1: Figure S1.** Schematic overview of the experimental timeline. (A) Sciatic nerve crush injury model was established 500 ng MaR1 was applied by sponge bandage to the injured leg. The motor functions and sensory functions were monitored from 3 days after injury to 8 weeks indicated by Fig. 3. (B) Intraplantar injection of MaR1 or NGF into the naïve mice to examine the effects of MaR1 and NGF on pain indicated by Fig. 6A, B. (C) CCI model was established and intrathecal injection of MaR1 was performed after 1 week injured, then mechanical allodynia and thermal hyperalgesia were measured at 1 h, 4H, and 24 h after application of MaR1, and the results are shown in Fig. 5A and B.

**Additional file 2: Figure S2.** NGF dose-dependently promoted axon regeneration in vitro. NGF stimulated neurite outgrowth in DRG neurons grown in explant and dissociated cultures in vitro. Neurofilament staining was employed to examine the outgrowth of neurites in the DRG. (A, B) NGF increased the neurite length and number in a dose-dependent manner. DRG neurons cultured with NGF for 36 h. (C, D) The EC50 values at which NGF increased the neurite length and number were measured. NGF was added in the culture medium. The data are presented as the mean ± SD, *p < 0.05 versus the control group, one-way ANOVA.

**Additional file 3: Table S1.** Statistical report.

**Additional file 4: Table S2.** *T* test comparison.

## Data Availability

The datasets used and/or analyzed during the current study are available from the corresponding author on reasonable request.

## Abbreviations

PNI: Peripheral nerve injury
NGF: Nerve growth factor
MaR1: Maresin 1
TRPV1: Transient receptor potential vanilloid 1
DRG: Dorsal root ganglion
CCI: Chronic constriction injury model
SFI: Sciatic function index
TNF-α: Tumor necrosis factor-alpha
IL-1β: Interleukin (IL)-1-beta
IL-6: Interleukin (IL)-6
ATF-3: Activating Transcription Factor 3
GFAP: Glial fibrillary acidic protein
IBA1: Ionized calcium binding adapter molecule 1
NF200: Neurofilament 200
p-AKT: Phosphorylated AKT
p-ERK: Phosphorylated ERK
p-PI3K: Phosphorylated PI3K
p-mTOR: Phosphorylated mTOR
BLT1: Leukotriene B4 receptor
LGR6: Leucine-rich repeat containing G protein-coupled receptor 6
RORα: Retinoic acid-related orphan receptor α
NASH: Nonalcoholic steatohepatitis
12-LOX: 12-Lipoxygenase
CMAPs: Compound muscle action potentials
