# Supplementary figures and images for: Maresin 1 promotes nerve regeneration and alleviates neuropathic pain after nerve injury
Source: J Neuroinflammation. 2022 Feb 2;19:32. doi: 10.1186/s12974-022-02405-1 (PMC8809034; doi:10.1186/s12974-022-02405-1)

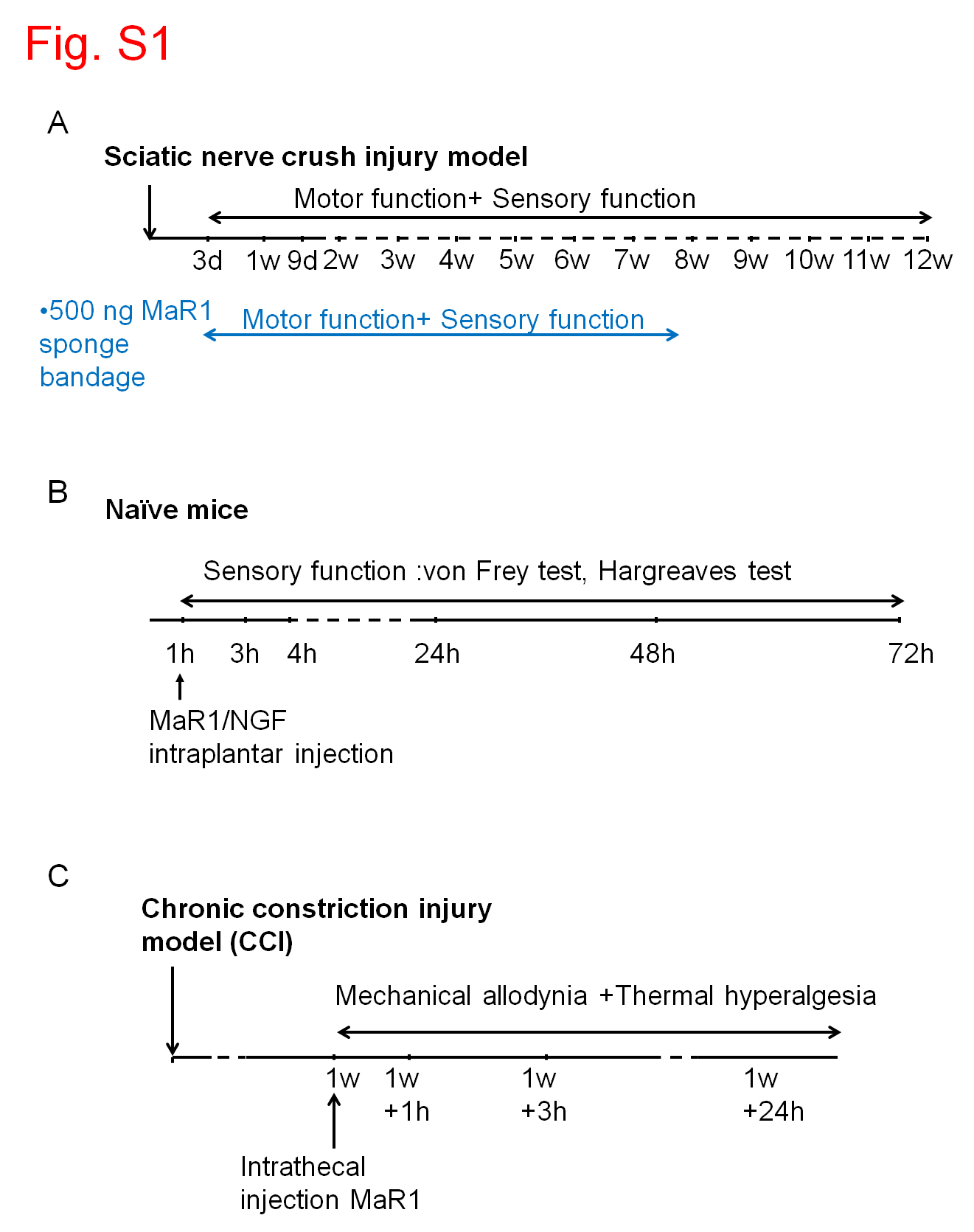

Supplement: Supplementary file 1 — Additional file 1: Figure S1. Schematic overview of the experimental timeline. (A) Sciatic nerve crush injury model was established 500 ng MaR1 was applied by sponge bandage to the injured leg. The motor functions and sensory functions were monitored from 3 days after injury to 8 weeks indicated by Fig. 3. (B) Intraplantar injection of MaR1 or NGF into the naïve mice to examine the effects of MaR1 and NGF on pain indicated by Fig. 6A, B. (C) CCI model was established and intrathecal injection of MaR1 was performed after 1 week injured, then mechanical allodynia and thermal hyperalgesia were measured at 1 h, 4H, and 24 h after application of MaR1, and the results are shown in Fig. 5A and B. [file 12974_2022_2405_MOESM1_ESM.jpg]

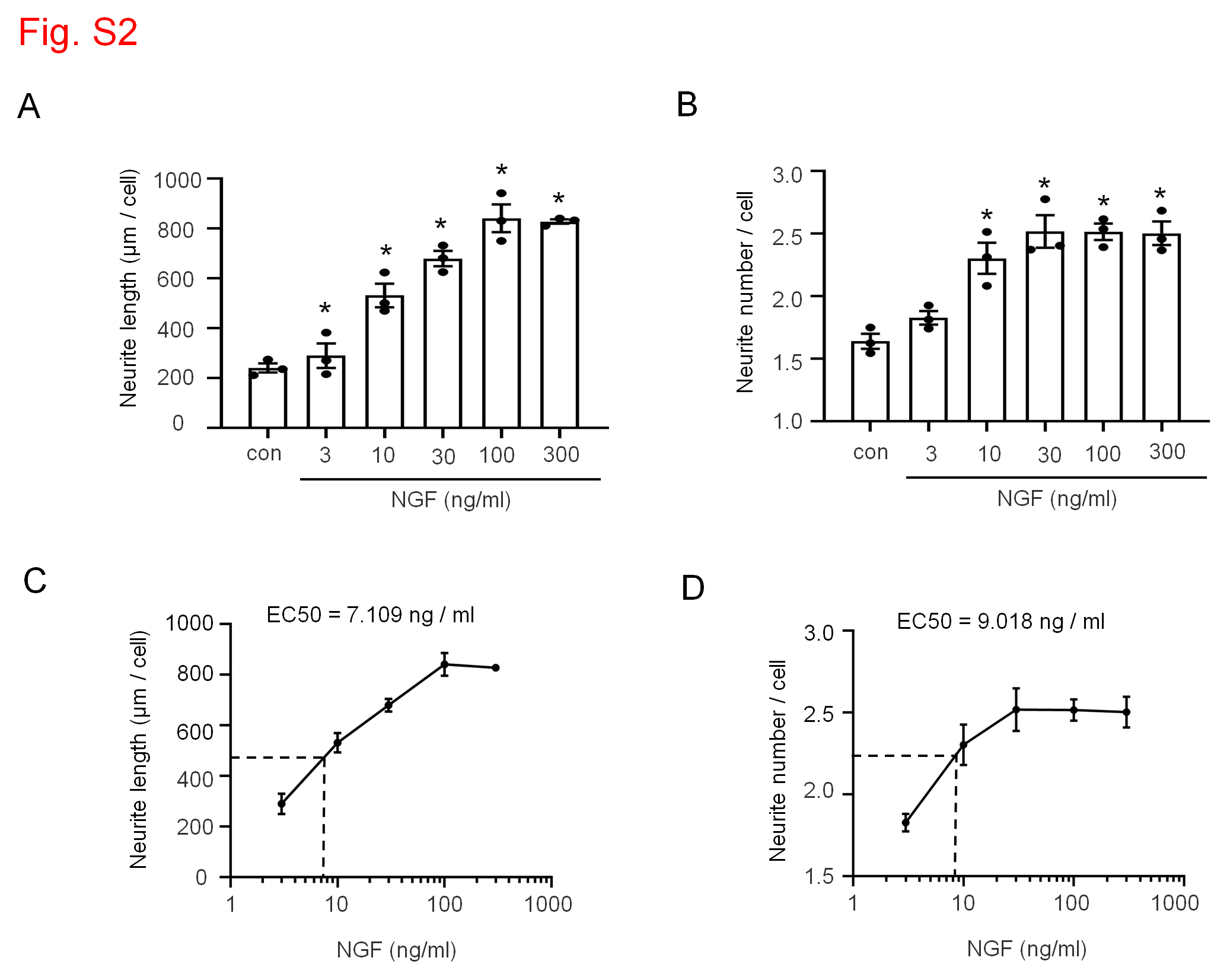

Supplement: Supplementary file 2 — Additional file 2: Figure S2. NGF dose-dependently promoted axon regeneration in vitro. NGF stimulated neurite outgrowth in DRG neurons grown in explant and dissociated cultures in vitro. Neurofilament staining was employed to examine the outgrowth of neurites in the DRG. (A, B) NGF increased the neurite length and number in a dose-dependent manner. DRG neurons cultured with NGF for 36 h. (C, D) The EC50 values at which NGF increased the neurite length and number were measured. NGF was added in the culture medium. The data are presented as the mean ± SD, *p < 0.05 versus the control group, one-way ANOVA. [file 12974_2022_2405_MOESM2_ESM.jpg]
