# Supplementary material for: Maresin 1 promotes nerve regeneration and alleviates neuropathic pain after nerve injury
Source: J Neuroinflammation. 2022 Feb 2;19:32. doi: 10.1186/s12974-022-02405-1 (PMC8809034; doi:10.1186/s12974-022-02405-1)
Supplement: Supplementary file 3 — Additional file 3: Table S1. Statistical report. [file 12974_2022_2405_MOESM3_ESM.pdf]

Table 1 Statistical report

	TEST USED	Animal		DESCRIPTIVE STATS (AVERAGE,VARIANCE)		P VALUE	DEGREE OF FREEDOM & F/t/z/R/ETC VALUE	E value
FIGURE NUMBER	WHICH TEST?	EXACT VALUE	DEFINED?	Mean	SD	EXACT VALUE	VALUE	E value
Fig.1A	One-way ANOVA Bonferroni post hoc	8	Adult male mice (8-10 weeks)	-24.68125<br>-33.66875<br>-30.32<br>-28.975<br>-35.18375<br>-34.73875<br>-30.2475<br>-22.64375	20.6424<br>31.76186<br>27.4817<br>31.49203<br>27.6097<br>34.97856<br>26.89575<br>20.3996	P<0.0001	F (7, 56) = 104.9	14
Fig. 1B	One-way ANOVA Bonferroni post hoc	8	Adult male mice (8-10 weeks)	300<br>20.25<br>39.25<br>41.63<br>45.38<br>53.13<br>60<br>64.25<br>70.25<br>66.88<br>74.13<br>78.25<br>80.13<br>89.38	0<br>11.88<br>22.86<br>20.6<br>16.67<br>15.93<br>14.86<br>12.06<br>9.633<br>11.84<br>10.03<br>10.9<br>9.891<br>9.561	P<0.0001	F (13, 98) = 190.1	14

Fig.1C	One-way ANOVA<br>Bonferroni post hoc	8	Adult male mice<br>(8-10 weeks)	0.99<br>3.878<br>1.663<br>0.5138<br>0.05<br>0.04125<br>0.1075<br>0.1238<br>0.1063<br>0.1388<br>0.14<br>0.175<br>0.2263<br>0.2463	0.2286<br>0.8536<br>0.3682<br>0.1608<br>0.03817<br>0.03227<br>0.06541<br>0.02774<br>0.06413<br>0.06081<br>0.05855<br>0.09165<br>0.09782<br>0.09273	P<0.0001	F (13, 98) = 124.1	14
Fig.1D	One-way ANOVA<br>Bonferroni post hoc	8	Adult male mice<br>(8-10 weeks)	10.66<br>17.47<br>10.53<br>7.73<br>5.444<br>4.705<br>6.23<br>5.285<br>6.436<br>6.221<br>7.228<br>8.343<br>9.19<br>10.18	0.5838<br>1.581<br>1.349<br>0.8462<br>0.6968<br>0.9036<br>0.8362<br>1.362<br>0.7654<br>0.9818<br>1.345<br>1.041<br>0.6317<br>0.7216	P<0.0001	F (13, 98) = 84.14	14

Fig.2A	One-way ANOVA<br>Bonferroni post hoc	6,6,6,6	Adult male mice<br>(8-10 weeks)	Naïve<br>-7.5<br>-7.966667<br>-7.916667<br>-7.9<br>-8<br>Sham<br>-7.5<br>-7.983334<br>-7.95<br>-7.816667<br>-8.05<br>sham+vehicle<br>-7.5<br>-8.016666<br>-8.05<br>-7.883333<br>-7.866667<br>sham+MaR1<br>-7.5<br>-7.816667<br>-7.933333<br>-7.883333<br>-7.933333	Naïve<br>0<br>0.2581988<br>0.3125168<br>0.1673322<br>0.3033149<br>Sham<br>0<br>0.2041243<br>0.3449639<br>0.2639445<br>0.3146426<br>sham+vehicle<br>0<br>0.3488075<br>0.2880971<br>0.3710345<br>0.3011091<br>sham+MaR1<br>0<br>0.2639444<br>0.2943921<br>0.2483277<br>0.350238	P=0.9757	F (12, 100) = 0.3548	20

Fig.2B	One-way ANOVA<br>Bonferroni post hoc	6,6,6,6	Adult male mice<br>(8-10 weeks)	Naïve<br>300.5<br>300.5<br>302<br>303.5<br>300.8333<br>Sham<br>300.3333<br>300.5<br>302.8333<br>301.8333<br>301.6667<br>sham+vehicle<br>300.6667<br>301.3333<br>302<br>303<br>302.1667<br>sham+MaR1<br>300.8333<br>301.3333<br>302.8333<br>301.6667<br>299.6667	Naïve<br>3.271086<br>1.643168<br>1.67332<br>2.428992<br>3.188521<br>Sham<br>2.160247<br>0.83666<br>0.7527726<br>2.483277<br>2.42212<br>sham+vehicle<br>2.33809<br>1.75119<br>1.67332<br>1.788854<br>1.47196<br>sham+MaR1<br>2.041241<br>1.032796<br>1.169045<br>2.581989<br>1.861899	P=0.6554	F (12, 100) = 0.7942	20

Fig.2C	One-way ANOVA<br>Bonferroni post hoc	6,6,6,6	Adult male mice<br>(8-10 weeks)	Naïve<br>0.9933333<br>1.025<br>1.055<br>1.108333<br>1.01<br>Sham<br>0.9933333<br>0.9566666<br>1.063333<br>1.016667<br>1.1<br>sham+vehicle<br>0.9933333<br>0.9716666<br>1.031667<br>1.04<br>0.9866667<br>sham+MaR1<br>0.9933333<br>0.9866667<br>0.9866667<br>1.055<br>1.016667	Naïve<br>0.2703824<br>0.1873766<br>0.1930544<br>0.2115104<br>0.07348467<br>Sham<br>0.2703824<br>0.2024516<br>0.1364795<br>0.2301883<br>0.09295157<br>sham+vehicle<br>0.2703824<br>0.2694748<br>0.1637579<br>0.09295157<br>0.2192411<br>sham+MaR1<br>0.2703824<br>0.2192411<br>0.2192411<br>0.1930544<br>0.2301883	P=0.9994	F (12, 100) = 0.1620	20

Fig.2D	One-way ANOVA, Bonferroni post hoc	6,6,6,6	Adult male mice (8-10 weeks)	Naïve<br>10.14833<br>9.991667<br>9.993334<br>10.38833<br>10.25833<br>Sham<br>10.45<br>10.17833<br>10.26<br>10.11667<br>10.05<br>sham+vehicle<br>10.375<br>10.36<br>10.40833<br>10.25167<br>10.32167<br>sham+MaR1<br>10.63167<br>10.48667<br>10.38<br>10.28667<br>10.04833	Naïve<br>0.5438903<br>0.3969592<br>0.1901229<br>0.4056315<br>0.6670957<br>Sham<br>0.4258168<br>0.1720949<br>0.4353848<br>0.1351543<br>0.2068819<br>sham+vehicle<br>0.6155241<br>0.5092348<br>0.4234817<br>0.310639<br>0.4649481<br>sham+MaR1<br>0.4752859<br>0.3771827<br>0.3896667<br>0.2922101<br>0.4534055	P=0.5661	F (12, 100) = 0.8834	20
Fig.3C	One-way ANOVA Bonferroni post hoc	3,3,3,3,3,3,3	mouse DRG neuron	240.2<br>281.1<br>372.6<br>509.5<br>701.4<br>843.1<br>861.4	35.38<br>60.73<br>88.57<br>53.76<br>86.43<br>92.12<br>43.51	P<0.0001	F (6, 14) = 42.50	14

Fig.3D	One-way ANOVA<br>Bonferroni post hoc	3,3,3,3,3,3,3	mouse DRG neuron	1.639<br>1.642<br>1.885<br>2.094<br>2.338<br>2.538<br>2.543	0.1035<br>0.1179<br>0.1102<br>0.1041<br>0.1652<br>0.1484<br>0.07485	P<0.0001	F (6, 14) = 31.19	14
Fig.3G	Unpaired T test	3,3	mouse DRG neuron	240.2<br>843.1<br>534.6<br>861.4<br>851.5	35.38<br>92.12<br>75.97<br>43.51<br>37.78	P=0.0110<br>P=0.7793	t=4.476, df=4<br>t=0.2998, df=4	
Fig.3G	One-way ANOVA<br>Bonferroni post hoc	3,3,3,3,3	mouse DRG neuron	240.2<br>843.1<br>534.6<br>861.4<br>851.5	35.38<br>92.12<br>75.97<br>43.51<br>37.78	P<0.0001	F (4, 10) = 60.26	10
Fig.3H	Unpaired T test	3,3	mouse DRG neuron	1.639<br>2.538<br>2.321<br>2.543<br>2.535	0.1035<br>0.1484<br>0.1604<br>0.1749<br>0.1777	P=0.1594<br>P=0.9566	t=1.726, df=4<br>t=0.05790, df=4	

Fig.3H	One-way ANOVA Bonferroni post hoc	3,3,3,3,3	mouse DRG neuron	1.639<br>2.538<br>2.321<br>2.543<br>2.535	0.1035<br>0.1484<br>0.1604<br>0.1749<br>0.1777	P=0.0001	F (4, 10) = 18.89	10
Fig.4B	One-way ANOVA Bonferroni post hoc	12,12,12	Dorsal root ganglion	0<br>53.58<br>81.75	0<br>12.7<br>10.43	P<0.0001	F (2, 33) = 229.9	33
Fig.4D	One-way ANOVA Bonferroni post hoc	12,12,12	Dorsal root ganglion	14.17<br>6.083<br>3.917	2.823<br>2.392<br>1.881	P<0.0001	F (2, 33) = 60.98	33
Fig.4F	Unpaired T test	6,7	Muscle	53.65<br>67.85	4.254<br>7.64	P=0.0020	t=4.032, df=11	

Fig.4G	Two-way ANOVA<br>Bonferroni post hoc	6,6,6	Adult male mice<br>(8-10 weeks)	Vehicle<br>-7.5<br>-80.38834<br>-65.775<br>-45.66667<br>-22.38167<br>-11.81333<br>MaR1<br>-7.5<br>-62.415<br>-44.305<br>-17.77333<br>-8.941667<br>-8.263333<br>NGF<br>-7.5<br>-65.855<br>-49.57167<br>-20.11<br>-12.39167<br>-8.308333	Vehicle<br>0<br>6.971757<br>3.738585<br>8.480453<br>6.806333<br>3.760216<br>MaR1<br>0<br>5.310008<br>8.861524<br>3.454317<br>1.673946<br>1.20427<br>NGF<br>0<br>5.599031<br>9.253683<br>4.658961<br>3.188394<br>1.309418	P<0.0001	F (10, 90) = 7.832	15

Fig.4H	Two-way ANOVA<br>Bonferroni post hoc	6,6,6	Adult male mice<br>(8-10 weeks)	Vehicle<br>300<br>20.16667<br>46<br>47.83333<br>49.5<br>52.83333<br>55.33333<br>70<br>78.83334<br>75<br>MaR1<br>300<br>48.33333<br>67<br>81.33334<br>103.8333<br>122.3333<br>134.5<br>145.6667<br>159.5<br>178.1667<br>NGF<br>300<br>49.83333<br>57.83333<br>71.83334<br>82.66666<br>104.5<br>120.3333<br>121.6667<br>151.3333<br>166.5	Vehicle<br>0<br>9.703951<br>22.56546<br>20.21303<br>17.24819<br>16.55798<br>12.53262<br>8.694826<br>9.174239<br>7.536578<br>MaR1<br>0<br>9.500877<br>10.8074<br>9.136009<br>14.07717<br>14.63785<br>16.71825<br>13.93796<br>10.91329<br>18.26928<br>NGF<br>0<br>9.907909<br>8.01041<br>9.703951<br>11.48332<br>19.42936<br>17.38582<br>15.39697<br>14.30618<br>18.76966	P<0.0001	F (18, 150) = 10.70	15

Fig.4I	Two-way ANOVA<br>Bonferroni post hoc	6,6,6	Adult male mice<br>(8-10 weeks)	Vehicle<br>0.995<br>3.853333<br>1.696667<br>0.53<br>0.06<br>0.05666666<br>0.09666666<br>0.1016667<br>0.075<br>0.1316667<br>MaR1<br>0.9416667<br>3.388333<br>1.01<br>0.675<br>0.4816667<br>0.46<br>0.4966667<br>0.6566666<br>0.675<br>0.865<br>NGF<br>0.995<br>3.573333<br>0.9566666<br>0.53<br>0.2816667<br>0.2533333<br>0.1366667<br>0.12<br>0.115<br>0.2466667	Vehicle<br>0.1754708<br>0.4265521<br>0.4291698<br>0.1479189<br>0.03521363<br>0.03444803<br>0.03204164<br>0.02228602<br>0.03209361<br>0.03488075<br>MaR1<br>0.09389712<br>0.4916267<br>0.2470627<br>0.1756986<br>0.1449713<br>0.1764086<br>0.1790717<br>0.2003663<br>0.1756986<br>0.1259762<br>NGF<br>0.1754708<br>0.6625004<br>0.2024516<br>0.1479189<br>0.1202359<br>0.1165619<br>0.0273252<br>0.03464102<br>0.04806246<br>0.07814517	P<0.0001	F (18, 100) = 6.945	15

Fig.4J	Two-way ANOVA Bonferroni post hoc	6,6,6	Adult male mice (8-10 weeks)	Vehicle<br>10.6<br>17.5<br>10.75<br>7.95<br>7.183333<br>4.966667<br>6.2<br>5.816667<br>6.35<br>6.2<br>MaR1<br>10.98333<br>15.85<br>8.316667<br>8.05<br>7.9<br>8.216667<br>8.966667<br>9.283333<br>9.183333<br>9.933333<br>NGF<br>10.38333<br>14.61667<br>7.866667<br>7.183333<br>5.966667<br>5.383333<br>5.883333<br>6.233334<br>6.8<br>7.216667	Vehicle<br>0.5291505<br>1.508642<br>1.723659<br>1.569395<br>1.321237<br>1.39523<br>1.2<br>1.074089<br>0.8871301<br>0.8763561<br>MaR1<br>0.5776388<br>1.593424<br>1.361494<br>1.100455<br>1.126055<br>1.330288<br>1.013246<br>0.8612007<br>0.8158838<br>0.7447597<br>NGF<br>0.457894<br>1.769086<br>1.214358<br>1.160029<br>1.376469<br>1.077806<br>0.7547628<br>0.7089899<br>0.9859005<br>0.9907911	P<0.0001	F (18, 150) = 5.896	15

Fig.5A	Two-way ANOVA<br>Bonferroni post hoc	6,6,6	Adult male mice<br>(8-10 weeks)	Vehicle<br>1.048333<br>0.225<br>0.2283333<br>0.2366667<br>0.235<br>10 ng/ml MaR1<br>0.995<br>0.2216667<br>0.5116667<br>0.4083333<br>0.23<br>100 ng/ml MaR1<br>1.01<br>0.24<br>0.7516667<br>0.75<br>0.2483333	Vehicle<br>0.2143284<br>0.05504543<br>0.06013872<br>0.1038589<br>0.04806245<br>10 ng/ml MaR1<br>0.1754708<br>0.09558591<br>0.157406<br>0.1734839<br>0.1100909<br>100 ng/ml MaR1<br>0.2470627<br>0.08717798<br>0.2183041<br>0.2641212<br>0.07808115	P<0.0001	F (8, 75) = 5.193	15

Fig.5B	Two-way ANOVA Bonferroni post hoc	6,6,6	Adult male mice (8-10 weeks)	Vehicle<br>10.56667<br>4.866667<br>4.85<br>5.1<br>5.016666<br>10 ng/ml MaR1<br>10.68333<br>4.9<br>6.883333<br>6.466667<br>4.8<br>100 ng/ml MaR1<br>10.53333<br>5.116667<br>8.516666<br>8.2<br>4.9	Vehicle<br>0.6918575<br>1.177568<br>1.127386<br>0.9777524<br>1.170328<br>10 ng/ml MaR1<br>0.9410987<br>0.9423376<br>1.228685<br>1.162182<br>0.6870226<br>100 ng/ml MaR1<br>0.6860515<br>0.9826835<br>1.307542<br>1.0583<br>0.8414273	P<0.0001	F (8, 75) = 5.018	15
Fig.5C	Unpaired T test	9,9	mouse DRG neuron	266.1<br>57.12	58.25<br>33.18	P<0.0001	t=9.353, df=16	
Fig.5F GFAP	Two-way ANOVA Bonferroni post hoc	4,4	Spinal Dorsal horn	Control<br>8.475<br>9.175<br>Ipsi<br>22.4<br>14.3	Control<br>2.707243<br>2.291106<br>Ipsi<br>5.303458<br>4.140048	P=0.0138	F (1, 3) = 27.13	12
Fig.5F GFAP vehicle	Unpaired T test	4,4	Spinal Dorsal horn	8.475<br>22.4	2.707<br>5.303	P=0.0034	t=4.677, df=6	

Fig.5F GFAP MaR1	paired T test	4,4	Spinal Dorsal horn	9.175<br>14.3	2.291<br>4.14	P=0.0201	t=4.532, df=3	
Fig.5F IBA1	Two-way ANOVA Bonferroni post hoc	4,4	Spinal Dorsal horn	Control<br>5.075<br>5.825<br>Ipsi<br>17.9<br>9.625	Control<br>1.517399<br>1.560716<br>Ipsi<br>3.939543<br>1.951709	P=0.0501	F (1, 3) = 10.12	12
Fig.5F IBA1 vehicle	Unpaired T test	4,5	Spinal Dorsal horn	5.075<br>17.9	1.517<br>3.94	P=0.0009	t=6.076, df=6	
Fig.5F IBA1 MaR1	Unpaired T test	4,6	Spinal Dorsal horn	5.825<br>9.625	1.561<br>1.952	P=0.0228	t=3.041, df=6	
Fig.5G IL-1β	Two-way ANOVA Bonferroni post hoc	4,4	Spinal Dorsal horn	Control<br>1<br>1<br>Ipsi<br>18.35<br>8.3	Control<br>0.2684523<br>0.2786874<br>Ipsi<br>3.711693<br>1.766352	P=0.0366	F (1, 3) = 12.99	12
Fig.5G IL-1β vehicle	Unpaired T test	4,4	Spinal Dorsal horn	1<br>18.35	0.2685<br>3.712	p<0.0001	t=9.324, df=6	
Fig.5G IL-1β MaR1	Unpaired T test	4,4	Spinal Dorsal horn	1<br>8.3	0.2787<br>1.766	P=0.0002	t=8.165, df=6	

Fig.5G IL-6	Two-way ANOVA<br>Bonferroni post hoc	4,4	Spinal Dorsal horn	Control<br>1<br>1<br>Ipsi<br>9.525<br>2.725	Control<br>0.222411<br>0.2099206<br>Ipsi<br>1.652019<br>0.6751543	P=0.0032	F (1, 3) = 76.04	12
Fig.5G IL-6<br>vehicle	Unpaired T test	4,4	Spinal Dorsal horn	1<br>9.525	0.2224<br>1.652	P<0.0001	t=10.23, df=6	
Fig.5G IL-6<br>MaR1	Unpaired T test	4,4	Spinal Dorsal horn	1<br>2.725	0.2099<br>0.6752	P=0.0028	t=4.880, df=6	
Fig.5G TNF	Two-way ANOVA<br>Bonferroni post hoc	4,4	Spinal Dorsal horn	Control<br>1<br>1<br>Ipsi<br>2.7875<br>1.385	Control<br>0.3311596<br>0.284605<br>Ipsi<br>1.001977<br>0.53	P=0.0626	F (1, 3) = 8.400	12
Fig.5G TNF<br>vehicle	Unpaired T test	4,4	Spinal Dorsal horn	1<br>2.788	0.3312<br>1.002	P=0.0147	t=3.388, df=6	
Fig.5G TNF<br>MaR1	Unpaired T test	4,4	Spinal Dorsal horn	1<br>1.385	0.2846<br>0.53	P=0.2478	t=1.280, df=6	

Fig.6A	Two-way ANOVA<br>Bonferroni post hoc	6,6,6	Adult male mice<br>(8-10 weeks)	Vehicle<br>0.9566666<br>0.7383333<br>0.81<br>0.9416667<br>0.9566666<br>0.995<br>MaR1<br>0.9416667<br>0.7716666<br>0.865<br>0.9416667<br>0.995<br>1.033333<br>NGF<br>1.01<br>0.2566667<br>0.2883333<br>0.4083333<br>0.5116667<br>0.5583333	Vehicle<br>0.2024516<br>0.1281276<br>0.1372589<br>0.09389712<br>0.2024516<br>0.1754708<br>MaR1<br>0.09389712<br>0.1096206<br>0.1259762<br>0.09389712<br>0.1754708<br>0.1306394<br>NGF<br>0.2470627<br>0.09244818<br>0.1252863<br>0.1633911<br>0.157406<br>0.1772475	$P<0.0001$	$F(10, 90) = 4.676$	15

<table>
<tr><td>Fig.6B</td><td>Two-way ANOVA
Bonferroni post hoc</td><td>6,6,6</td><td>Adult male mice
(8-10 weeks)</td><td>Vehicle
10.33333
9.75
10.15
10.65
10.41667
MaR1
10.95
9.916667
10.3
11.18333
10.8
NGF
10.23333
6.333333
6.6
7.166667
7.783333</td><td>Vehicle
1.04435
1.021273
0.8408331
0.5958188
0.6524312
MaR1
0.8264382
0.8565434
0.6260989
0.6047039
0.7874008
NGF
0.861781
1.298717
0.9715967
0.8617812
0.6645801</td><td>P<0.0001</td><td>F (8, 75) = 5.416</td><td>15</td></tr>
<tr><td>Fig.6D AKT</td><td>One-way ANOVA
Bonferroni post hoc</td><td>3,3,3</td><td>ND7/23 cells</td><td rowspan="2">p-AKT/GAPDH
1
1.972
1.565
AKT/GAPDH
1
1.025
0.9829
p-AKT/AKT
1
1.861
1.57</td><td rowspan="2">p-AKT/GAPDH
0.3109
0.6122
0.2522
AKT/GAPDH
0.1356
0.1524
0.03843
p-AKT/AKT
1
1.861
1.57</td><td>P=0.0784
P=0.9111
P=0.0400</td><td>F (2, 6) = 4.010
F (2, 6) = 0.09458
F (2, 6) = 5.772</td><td></td></tr>
<tr><td>Fig.6D AKT</td><td>paired T test
Unpaired T test</td><td>3,3</td><td>ND7/23 cells</td><td>P=0.0391
P=0.0354
P=0.8408
P=0.8443
P=0.0300
P=0.0489</td><td>t=4.910, df=2
t=2.445, df=4
t=0.2144, df=4
t=0.2095, df=4
t=3.296, df=4
t=2.152, df=4</td><td></td></tr>
</table>

Fig.6D ERK	One-way ANOVA<br>Bonferroni post hoc	3,3,3	ND7/23 cells	p-ERK/GAPDH<br>1<br>1.153<br>3.115<br>ERK/GAPDH<br>1<br>0.7987<br>0.8331<br>p-ERK/ERK<br>1<br>1.223<br>3.273	p-ERK/GAPDH<br>0.1653<br>0.2911<br>0.4435<br>ERK/GAPDH<br>0.4765<br>0.1648<br>0.1313<br>p-ERK/ERK<br>0.4746<br>0.1107<br>0.953	P=0.0003<br>P=0.6965<br>P=0.0075	F (2, 6) = 40.55<br>F (2, 6) = 0.3845<br>F (2, 6) = 12.33	
Fig.6D ERK	Unpaired T test	3,3	ND7/23 cells			P=0.4731<br>P=0.0015<br>P=0.5273<br>P=0.5900<br>P=0.4733<br>P=0.0209	t=0.7913, df=4<br>t=7.741, df=4<br>t=0.6916, df=4<br>t=0.5850, df=4<br>t=0.7908, df=4<br>t=3.697, df=4	
Fig.6F<br>mTOR	One-way ANOVA<br>Bonferroni post hoc	3,3,3	ND7/23 cells	p-mTOR/GAPDH<br>1<br>2.067<br>1.297<br>mTOR/GAPDH<br>1<br>0.9743<br>1.088<br>p-mTOR/mTOR<br>1<br>2.255<br>1.21	p-mTOR/GAPDH<br>0.8863<br>0.806<br>0.6194<br>mTOR/GAPDH<br>0.4299<br>0.3777<br>0.3092<br>p-mTOR/mTOR<br>0.774<br>0.7614<br>0.5202	P=0.2962<br>P=0.9275<br>P=0.1381	F (2, 6) = 1.501<br>F (2, 6) = 0.07625<br>F (2, 6) = 2.804	
Fig.6F<br>mTOR	paired T test<br>Unpaired T test	3,3	ND7/23 cells			P=0.0136<br>P=0.2047<br>P=0.9418<br>P=0.7867<br>P=0.0047<br>P=0.1963	t=5.946, df=2<br>t=1.035, df=2<br>t=0.07773, df=4<br>t=0.2893, df=4<br>t=10.20, df=2<br>t=1.082, df=2	

Fig.6F PI3K	One-way ANOVA<br>Bonferroni post hoc	3,3,3	ND7/23 cells	p-PI3K/GAPDH<br>1<br>1.877<br>1.236<br>PI3K/GAPDH<br>1<br>0.9868<br>1.009<br>p-PI3K/PI3K<br>1<br>1.89<br>1.206	p-PI3K/GAPDH<br>0.176<br>0.5217<br>0.4909<br>PI3K/GAPDH<br>0.08996<br>0.1027<br>0.1401<br>p-PI3K/PI3K<br>0.09104<br>0.3604<br>0.3111	P=0.1029<br>P=0.9718<br>P=0.0186	F (2, 6) = 3.402<br>F (2, 6) = 0.02872<br>F (2, 6) = 8.319	
Fig.6F PI3K	paired T test<br>Unpaired T test	3,3	ND7/23 cells			P=0.0483<br>P=0.3005<br>P=0.8754<br>P=0.9315<br>P=0.0143<br>P=0.3317	t=4.382, df=2<br>t=0.6152, df=2<br>t=0.1671, df=4<br>t=0.09145, df=4<br>t=4.148, df=4<br>t=1.103, df=4	
Fig.6H<br>p-AKT/AKT	One-way ANOVA<br>Bonferroni post hoc	3,3,3	mouse DRG neuron	1<br>1.341<br>1.600	0.2792<br>0.125<br>0.1249	p=0.0235	F (2, 6) = 7.476	6
	paired T test<br>Unpaired T test	3,3,3	mouse DRG neuron			p=0.3337<br>p=0.0273	t=1.932, df=4<br>t=3.400, df=4	
Fig.6H<br>p-<br>mTOR/TOR	One-way ANOVA<br>Bonferroni post hoc	3,3,3	mouse DRG neuron	1<br>1.171<br>1.872	0.3503<br>0.1094<br>0.2684	p=0.0145	F (2, 6) = 9.302	6
	paired T test<br>Unpaired T test	3,3,3	mouse DRG neuron			p=0.4646<br>p=0.0267	t=0.8076, df=4<br>t=3.424, df=4	
Fig.6H<br>p-PI3K/PI3K	One-way ANOVA<br>Bonferroni post hoc	3,3,3	mouse DRG neuron	1<br>0.8478<br>2.383	0.2845<br>0.1661<br>0.2425	p=0.0004	F (2, 6) = 38.49	6
	paired T test<br>Unpaired T test	3,3,3	mouse DRG neuron			p=0.4684<br>p=0.0030	t=0.8002, df=4<br>t=6.408, df=4	

Fig.6H<br>p-ERK/ERK	One-way ANOVA<br>Bonferroni post hoc	3,3,3	mouse DRG neuron	1<br>1.499<br>1.148	0.1318<br>0.1586<br>0.1502	p=0.0154	F (2, 6) = 9.064	6
	paired T test<br>Unpaired T test	3,3,3	mouse DRG neuron			p=0.0138<br>p=0.2682	t=4.188, df=4<br>t=1.285, df=4	
Fig. S2A	One-way ANOVA<br>Bonferroni post hoc	3,3,3,3,3,3	mouse DRG neuron	240.3<br>289.4<br>531.4<br>679.6<br>841.4<br>828.1	31.6<br>85.14<br>81.82<br>53.43<br>96.35<br>14.9	P<0.0001	F (5, 12) = 44.99	12
Fig. S2B	One-way ANOVA<br>Bonferroni post hoc	3,3,3,3,3,3	mouse DRG neuron	1.639<br>1.827<br>2.303<br>2.518<br>2.515<br>2.503	0.1035<br>0.09296<br>0.2157<br>0.2245<br>0.114<br>0.1632	P<0.0001	F (5, 12) = 17.44	12
