# Supplementary material for: Maresin 1 promotes nerve regeneration and alleviates neuropathic pain after nerve injury
Source: J Neuroinflammation. 2022 Feb 2;19:32. doi: 10.1186/s12974-022-02405-1 (PMC8809034; doi:10.1186/s12974-022-02405-1)
Supplement: Supplementary file 4 — Additional file 4: Table S2. T test comparison. [file 12974_2022_2405_MOESM4_ESM.pdf]

Table 2 T test comparison

Contents

	Figure			Page
3G	10 ng/ml MaR1		……………………	1
3G	10 ng/ml NGF		……………………	2
3G	100 ng/ml MaR1		……………………	3
3G	100 ng/ml NGF		……………………	4
3H	10 ng/ml MaR1		……………………	5
3H	10 ng/ml NGF		……………………	6
3H	100 ng/ml MaR1		……………………	7
3H	100 ng/ml NGF		……………………	8
6D	p-AKT/GAPDH	Control	……………………	9
6D	p-AKT/GAPDH	MaR1	……………………	10
6D	p-AKT/GAPDH	NGF	……………………	11
6D	AKT/GAPDH	Control	……………………	12
6D	AKT/GAPDH	MaR1	……………………	13
6D	AKT/GAPDH	NGF	……………………	14
6D	p-AKT/AKT	Control	……………………	15
6D	p-AKT/AKT	MaR1	……………………	16
6D	p-AKT/AKT	NGF	……………………	17
6D	p-ERK/GAPDH	Control	……………………	18
6D	p-ERK/GAPDH	MaR1	……………………	19
6D	p-ERK/GAPDH	NGF	……………………	20
6D	ERK/GAPDH	Control	……………………	21
6D	ERK/GAPDH	MaR1	……………………	22
6D	ERK/GAPDH	NGF	……………………	23
6D	p-ERK/ERK	Control	……………………	24
6D	p-ERK/ERK	MaR1	……………………	25
6D	p-ERK/ERK	NGF	……………………	26
6F	p-mTOR/GAPDH	Control	……………………	27
6F	p-mTOR/GAPDH	MaR1	……………………	28
6F	p-mTOR/GAPDH	NGF	……………………	29
6F	mTOR/GAPDH	Control	……………………	30
6F	mTOR/GAPDH	MaR1	……………………	31
6F	mTOR/GAPDH	NGF	……………………	32
6F	p-mTOR/mTOR	Control	……………………	33
6F	p-mTOR/mTOR	MaR1	……………………	34
6F	p-mTOR/mTOR	NGF	……………………	35
6F	p-PI3K/GAPDH	Control	……………………	36
6F	p-PI3K/GAPDH	MaR1	……………………	37
6F	p-PI3K/GAPDH	NGF	……………………	38
6F	PI3K/GAPDH	Control	……………………	39
6F	PI3K/GAPDH	MaR1	……………………	40
6F	PI3K/GAPDH	NGF	……………………	41
6F	p-PI3K/PI3K	Control	……………………	42
6F	p-PI3K/PI3K	MaR1	……………………	43
6F	p-PI3K/PI3K	NGF	……………………	44

Fig 3G

10 ng/ml Maresin 1
process report

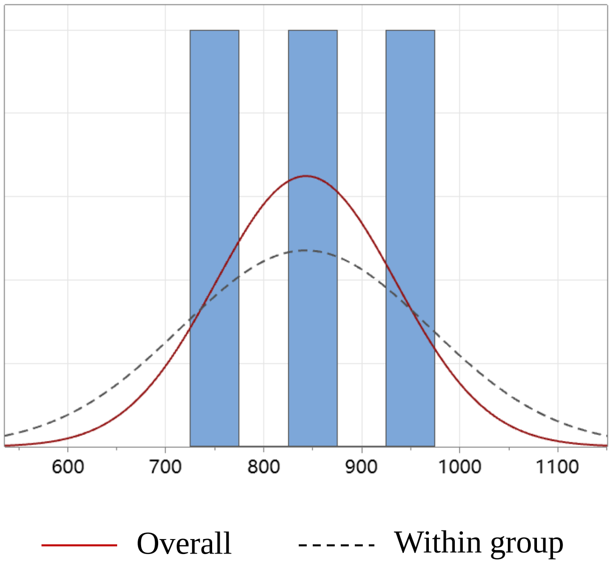

10 ng/ml Maresin 1
Normal distribution

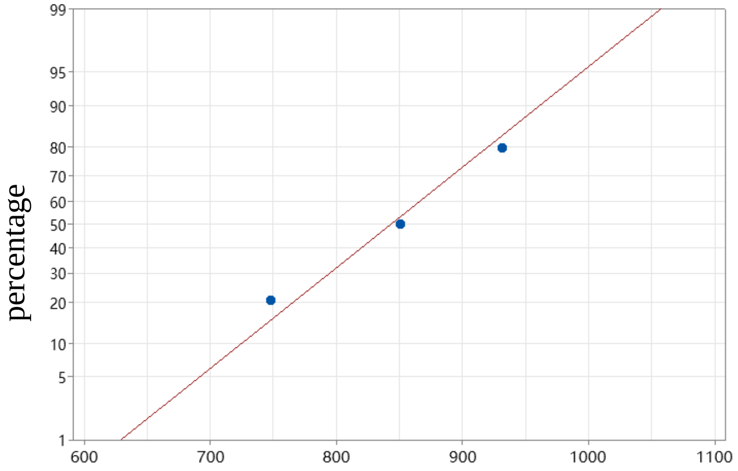

Process data	
Lower Specification Limit	100
Upper specification limit	400
Mean	843.143
N	3
SD （Overall）	92.1194
SD （Within group）	127.061

Overall	
Pp	0.54
PPL	2.69
PPU	-1.6
Ppk	-1.6
Cpm	*

Within group	
Cp	0.39
CPL	1.95
CPU	-1.16
Cpk	-1.16

Mean	84.31
SD	92.12
N	3
AD	0.195
P value	0.606

Fig 3G

10 ng/ml NGF
process report

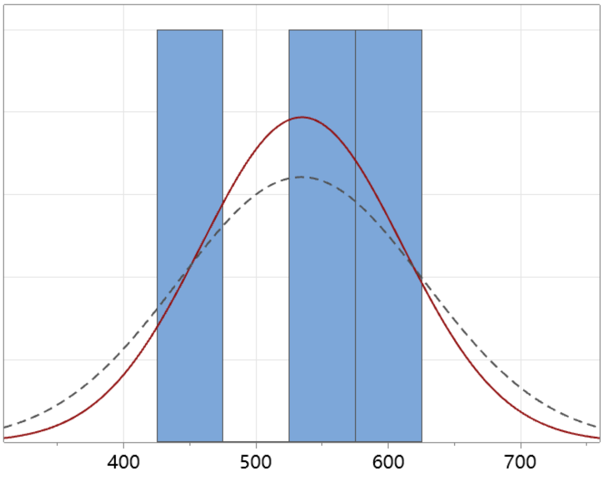

Overall ------ Within group

10 ng/ml NGF
Normal distribution

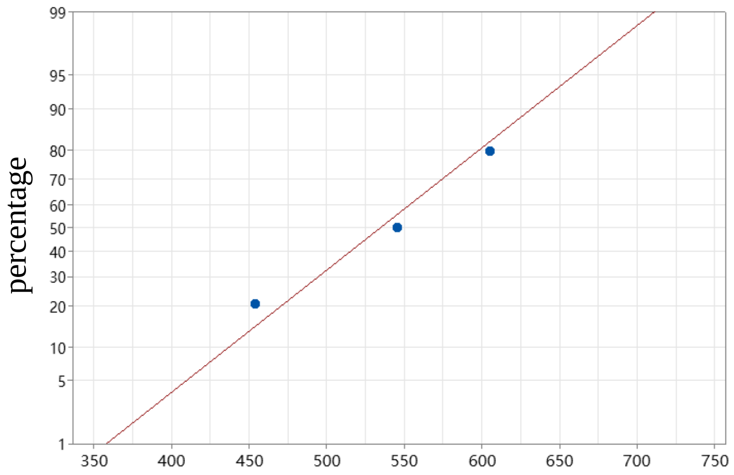

Process data	
Lower Specification Limit	100
Upper specification limit	400
Mean	534.58
N	3
SD （Overall）	75.9656
SD （Within group）	93.0708

Overall	
Pp	0.66
PPL	1.91
PPU	-0.59
Ppk	-0.59
Cpm	*

Within group	
Cp	0.54
CPL	1.56
CPU	-0.48
Cpk	-0.48

Mean	534.6
SD	75.97
N	3
AD	0.206
P value	0.557

Fig 3G

100 ng/ml Maresin 1
process report

100 ng/ml Maresin 1
Normal distribution

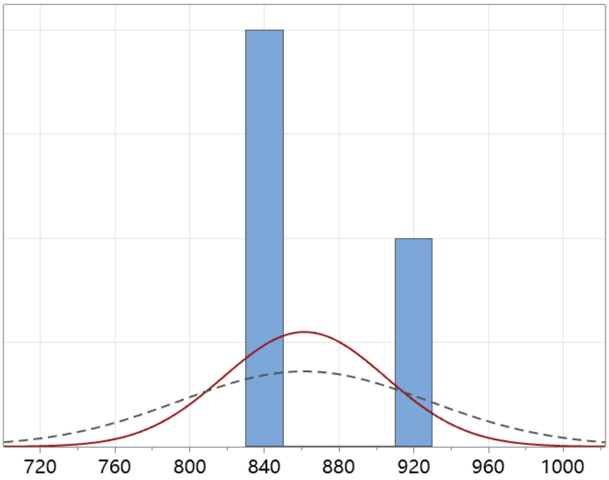

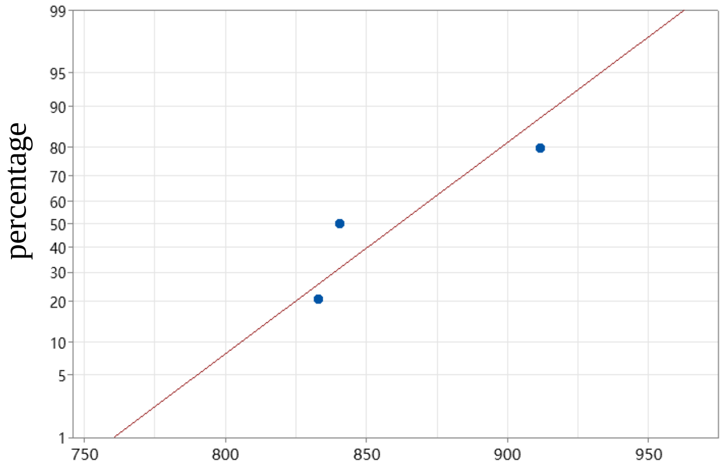

Process data	
Lower Specification Limit	100
Upper specification limit	400
Mean	861.43
N	3
SD （Overall）	43.5061
SD （Within group）	66.5452

Overall	
Pp	1.15
PPL	5.83
PPU	-3.54
Ppk	-3.54
Cpm	*

Within group	
Cp	0.75
CPL	3.81
CPU	-2.31
Cpk	-2.31

Mean	861.4
SD	43.51
N	3
AD	0.397
P value	0.12

Fig 3G

100 ng/ml NGF
process report

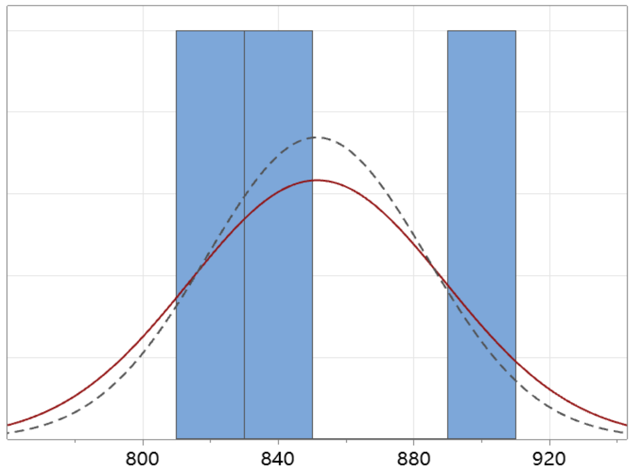

100 ng/ml NGF
Normal distribution

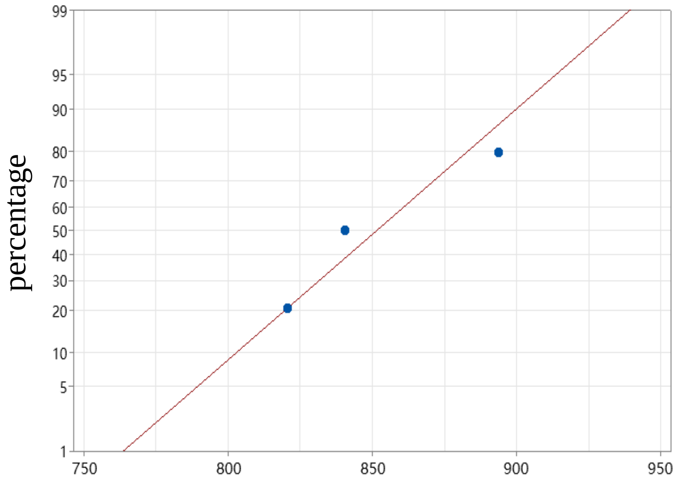

Process data	
Lower Specification Limit	100
Upper specification limit	400
Mean	851.456
N	3
SD （Overall）	37.7825
SD （Within group）	32.3987

Overall	
Pp	1.32
PPL	6.63
PPU	-3.98
Ppk	-3.98
Cpm	*

Within group	
Cp	1.54
CPL	7.73
CPU	-4.64
Cpk	-4.64

Mean	851.5
SD	37.78
N	3
AD	0.262
P value	0.375

Fig 3H

10 ng/ml Maresin 1
process report

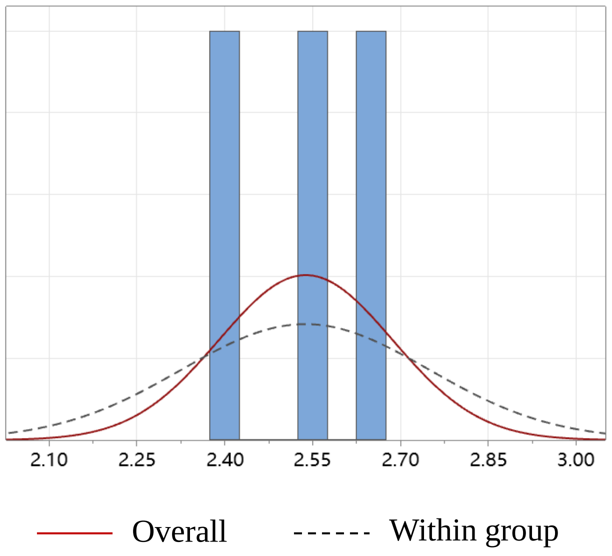

10 ng/ml Maresin 1
Normal distribution

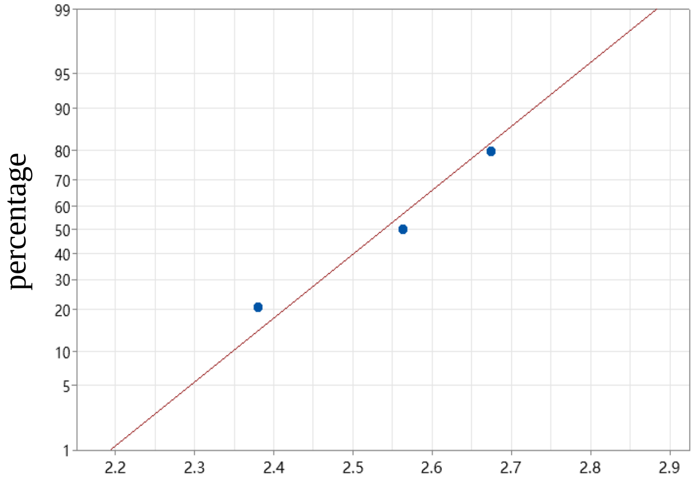

Process data	
Lower Specification Limit	100
Upper specification limit	400
Mean	2.5384
N	3
SD （Overall）	0.148412
SD （Within group）	0.211569

Overall	
Pp	336.9
PPL	-218.9
PPU	892.7
Ppk	-218.9
Cpm	*

Within group	
Cp	236.33
CPL	-153.55
CPU	626.21
Cpk	-153.55

Mean	2.538
SD	0.1484
N	3
AD	0.212
P value	0.536

Fig 3H

10 ng/ml NGF
process report

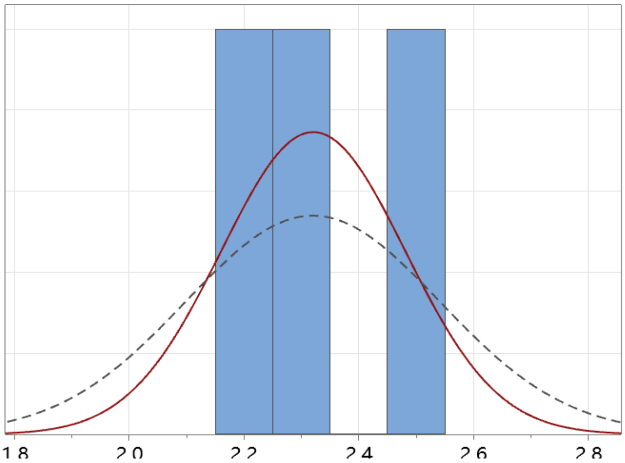

10 ng/ml NGF
Normal distribution

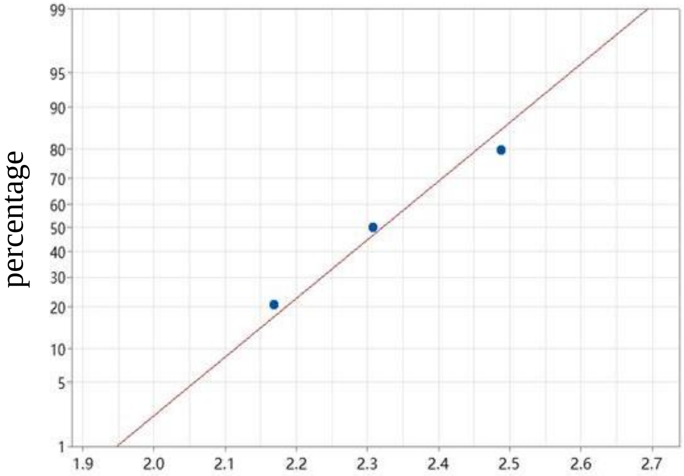

—— Overall ------ Within group

Process data	
Lower Specification Limit	100
Upper specification limit	400
Mean	2.32057
N	3
SD （Overall）	0.160424
SD （Within group）	0.22172

Overall	
Pp	311.67
PPL	-2020.96
PPU	826.31
Ppk	-202.96
Cpm	*

Within group	
Cp	225.51
CPL	-146.85
CPU	597.87
Cpk	-146.85

Mean	2.321
SD	0.1604
N	3
AD	0.195
P value	0.603

Fig 3H

100 ng/ml Maresin 1
process report

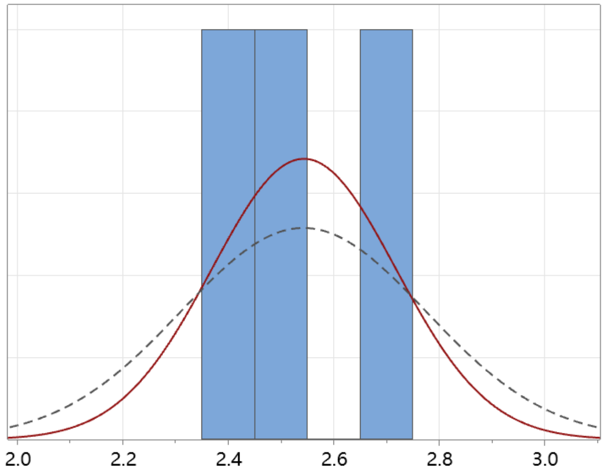

100 ng/ml Maresin 1
Normal distribution

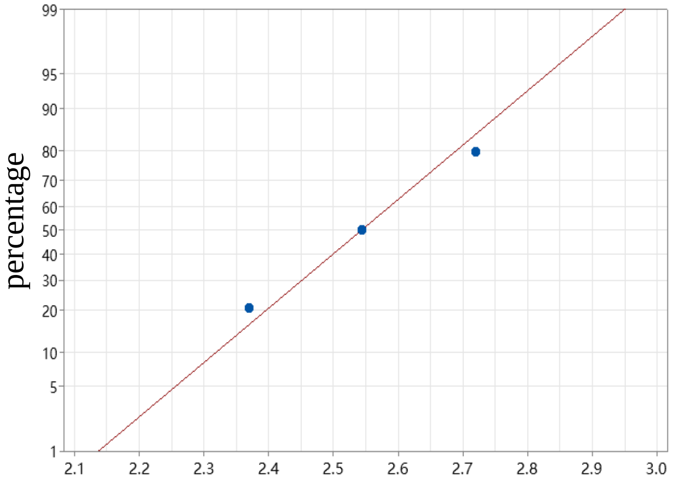

Process data	
Lower Specification Limit	1
Upper specification limit	4
Mean	2.5433
N	3
SD （Overall）	0.174851
SD （Within group）	0.232048

Overall	
Pp	2.86
PPL	2.94
PPU	2.78
Ppk	2.78
Cpm	*

Within group	
Cp	2.15
CPL	2.22
CPU	2.09
Cpk	2.09

Mean	2.543
SD	0.1749
N	3
AD	0.19
P value	0.631

Fig 3H

100 ng/ml NGF
process report

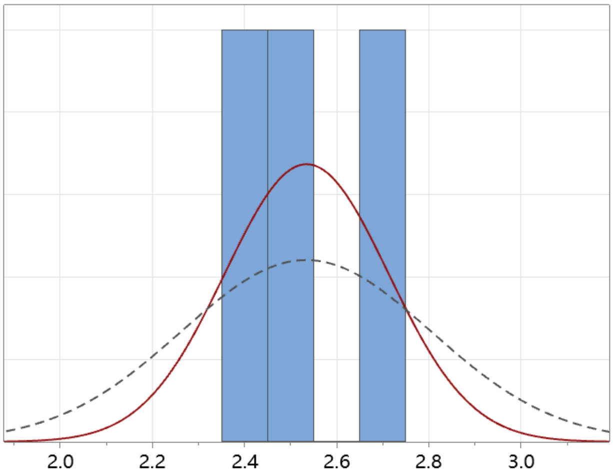

100 ng/ml NGF
Normal distribution

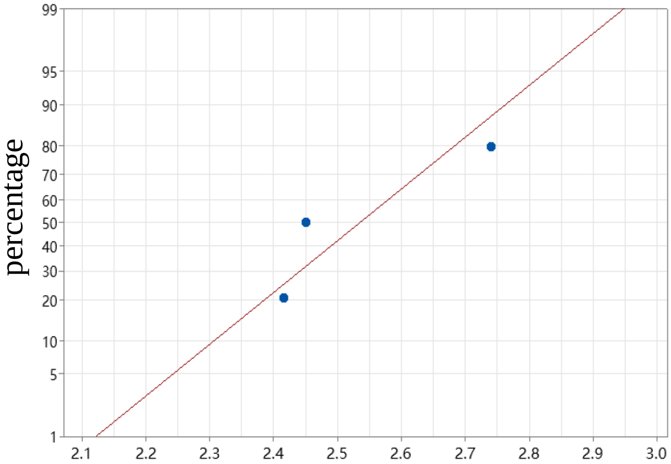

Process data	
Lower Specification Limit	1
Upper specification limit	4
Mean	2.53497
N	3
SD （Overall）	0.177659
SD （Within group）	0.271454

Overall	
Pp	2.81
PPL	2.88
PPU	2.75
Ppk	2.75
Cpm	*

Within group	
Cp	1.84
CPL	1.88
CPU	1.80
Cpk	1.80

Mean	2.535
SD	0.1777
N	3
AD	0.387
P value	0.131

Fig 6D p-AKT/GAPDH

Control
process report

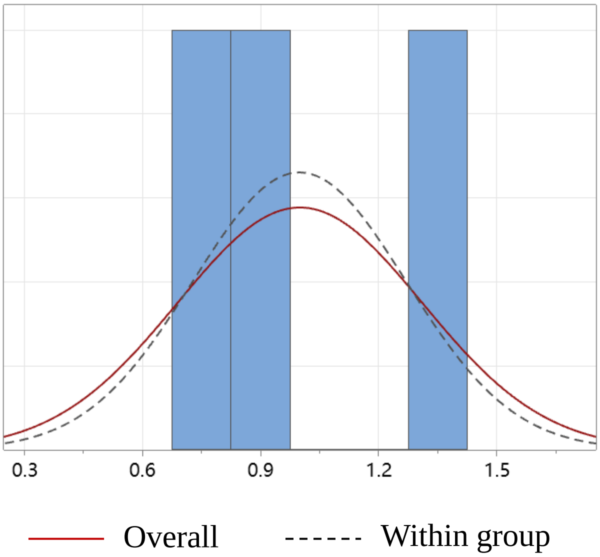

Control
Normal distribution

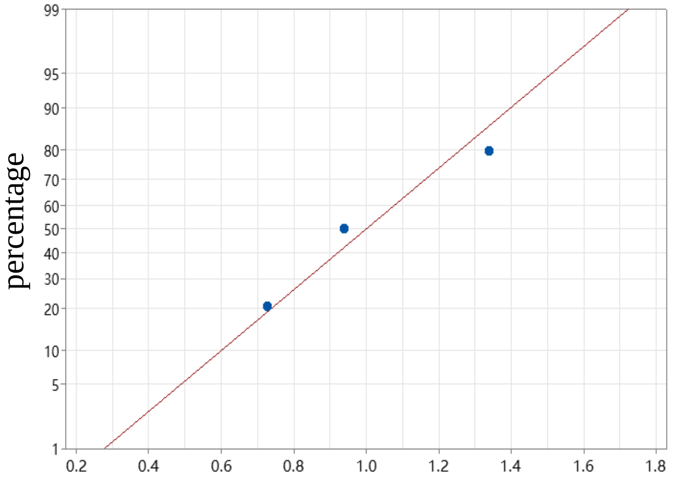

Process data	
Lower Specification Limit	0
Upper specification limit	1.5
Mean	1
N	3
SD （Overall）	0.31093
SD （Within group）	0.271503

Mean	1
SD	0.3109
N	3
AD	0.223
P value	0.508

Fig 6D p-AKT/GAPDH

Maresin 1
process report

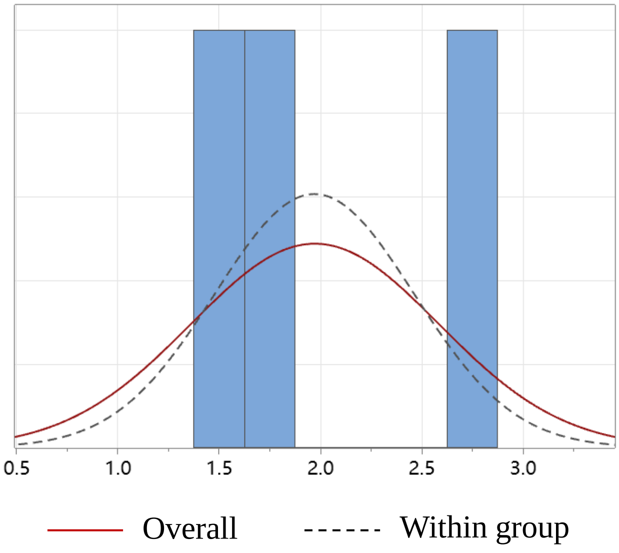

Maresin 1
Normal distribution

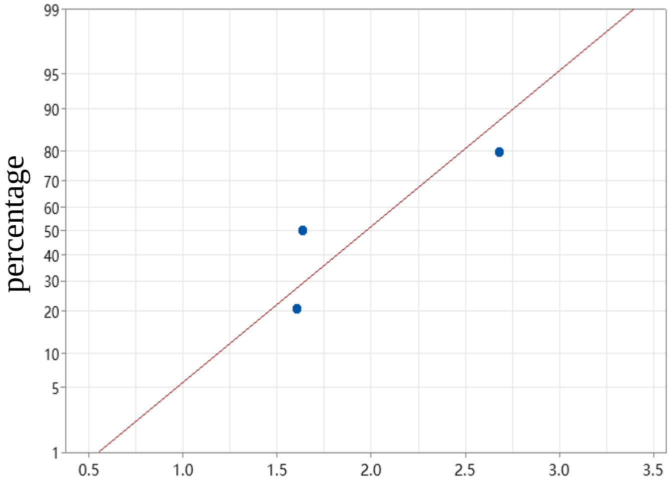

Process data	
Lower Specification Limit	0
Upper specification limit	3
Mean	1.97009
N	3
SD （Overall）	0.612245
SD （Within group）	0.492434

Mean	1.972
SD	0.6122
N	3
AD	0.458
P value	0.073

Fig 6D p-AKT/GAPDH

NGF
process report

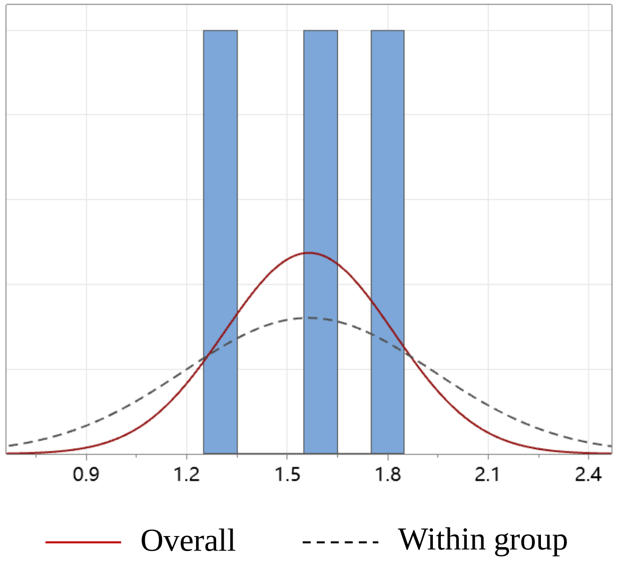

Process data	
Lower Specification Limit	0
Upper specification limit	3
Mean	1.56525
N	3
SD （Overall）	0.252193
SD （Within group）	0.373339

NGF
Normal distribution

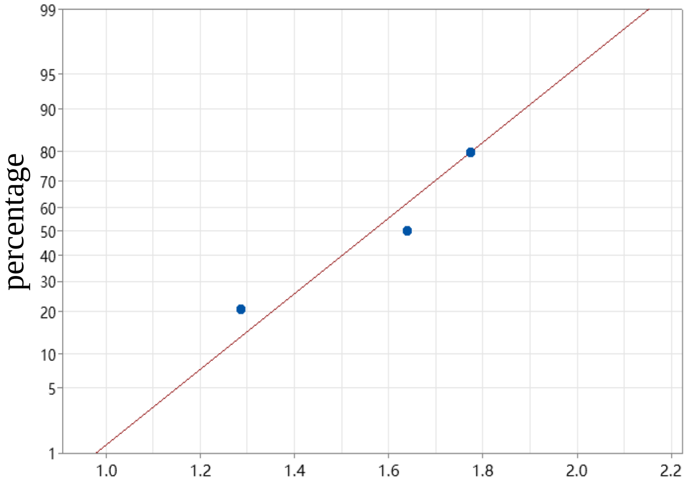

Mean	1.565
SD	0.2522
N	3
AD	0.261
P value	0.378

Fig 6D AKT/GAPDH

Control
process report

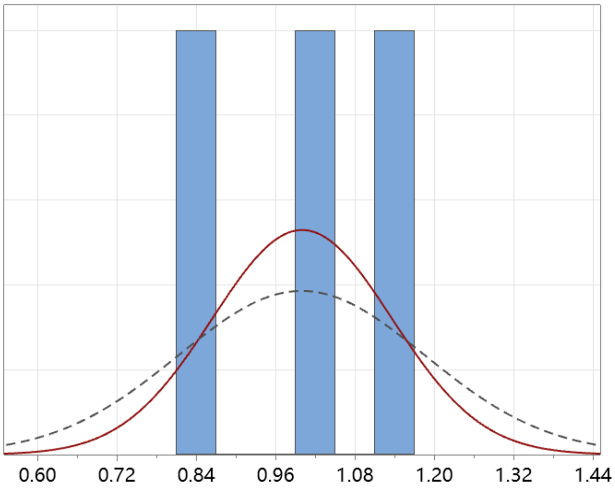

Control
Normal distribution

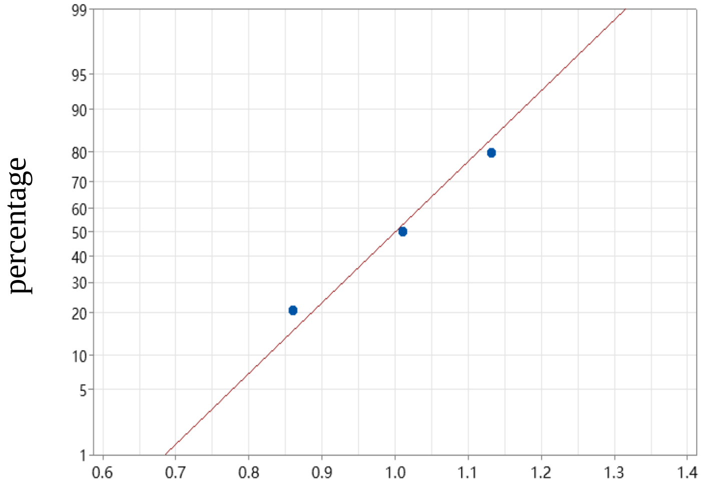

Overall ------ Within group

Process data	
Lower Specification Limit	0
Upper specification limit	3
Mean	1
N	3
SD （Overall）	0.135625
SD （Within group）	0.186267

Mean	1
SD	0.1356
N	3
AD	0.193
P value	0.612

Fig 6D AKT/GAPDH

Maresin 1
process report

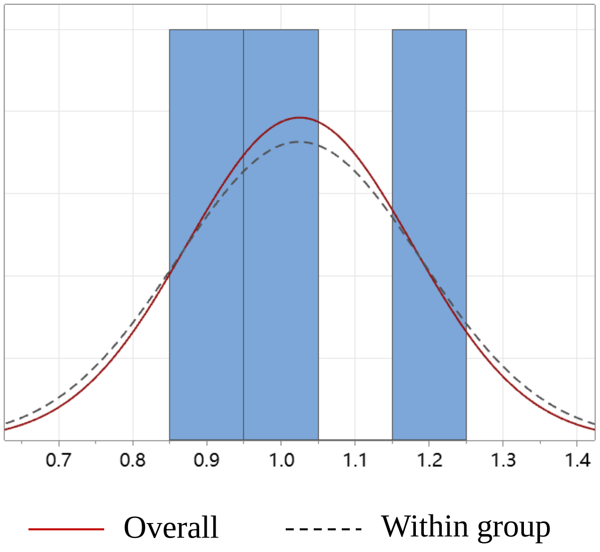

Maresin 1
Normal distribution

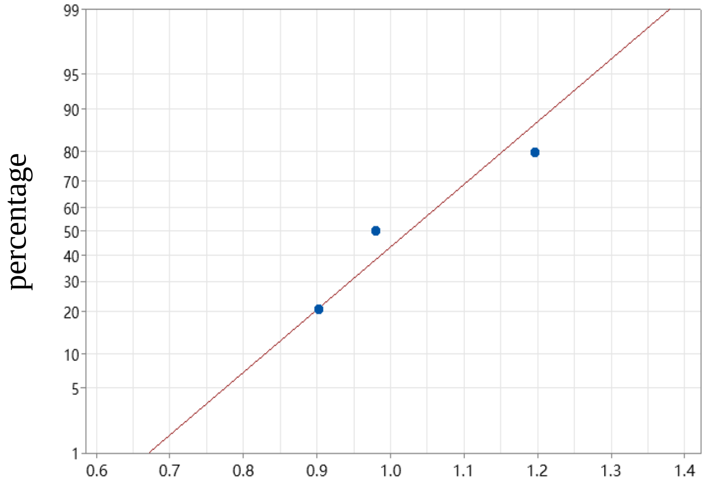

Process data	
Lower Specification Limit	0
Upper specification limit	3
Mean	1.02525
N	3
SD （Overall）	0.152395
SD （Within group）	0.164687

Mean	1.025
SD	0.1524
N	3
AD	0.268
P value	0.358

Fig 6D AKT/GAPDH

NGF
process report

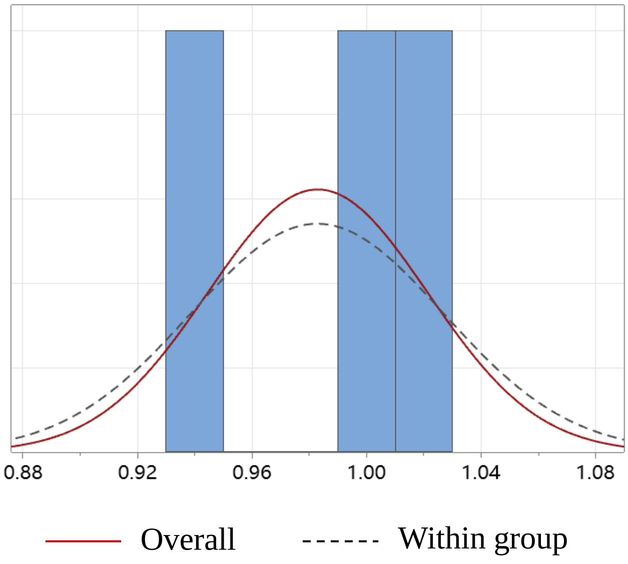

NGF
Normal distribution

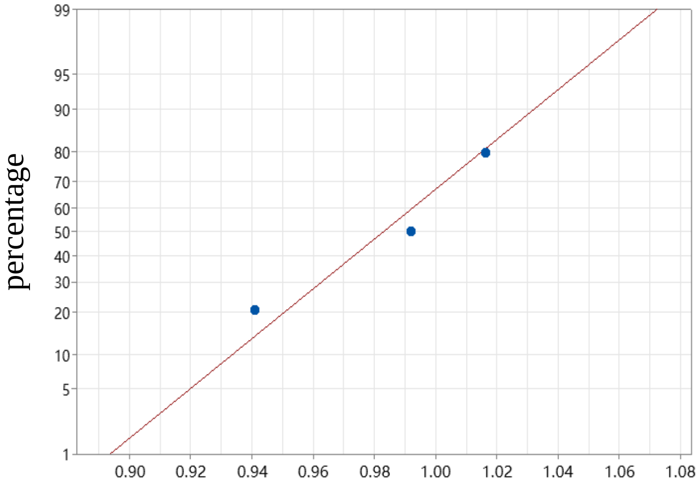

Process data	
Lower Specification Limit	0
Upper specification limit	3
Mean	0.98295
N	3
SD （Overall）	0.0374305
SD （Within group）	0.0441986

Mean	0.9829
SD	0.03843
N	3
AD	0.234
P value	0.470

Fig 6D p-AKT/AKT

Control
process report

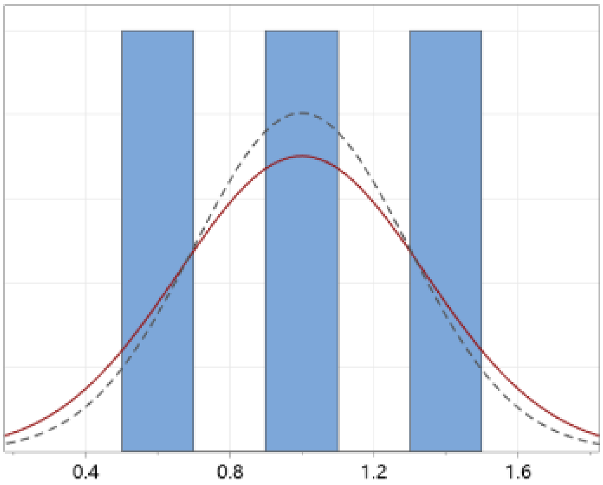

Overall ------ Within group

Control
Normal distribution

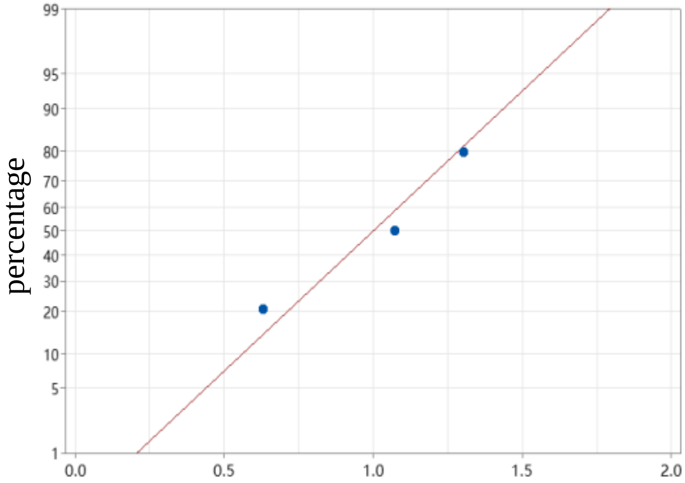

Process data	
Lower Specification Limit	0
Upper specification limit	3
Mean	1
N	3
SD （Overall）	0.34115
SD （Within group）	0.297513

Mean	1
SD	0.3411
N	3
AD	0.226
P value	0.503

Fig 6D p-AKT/AKT

Maresin 1
process report

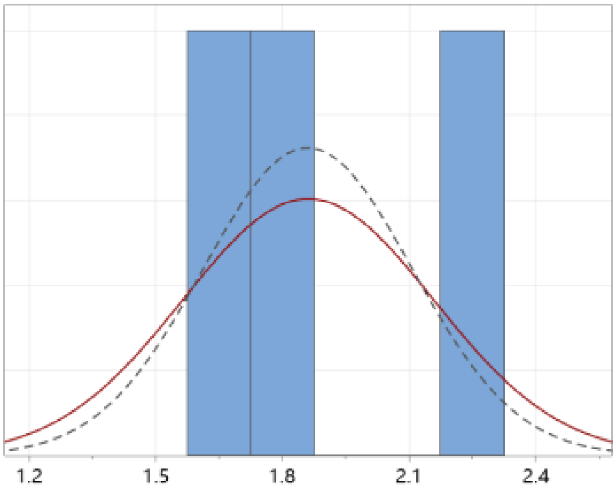

Maresin 1
Normal distribution

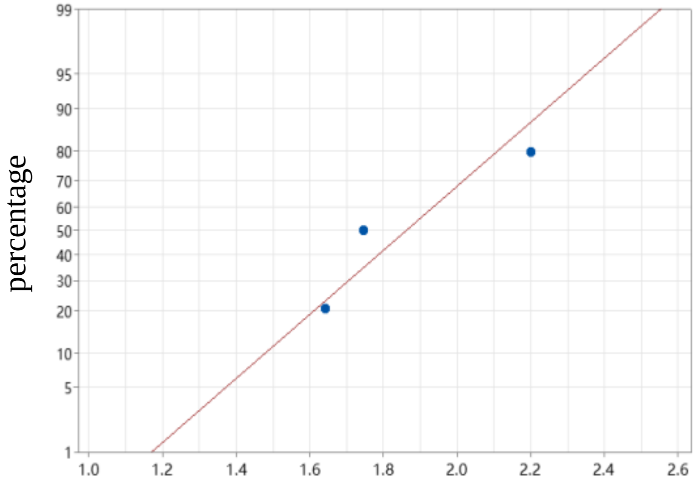

—— Overall ------ Within group

Process data	
Lower Specification Limit	0
Upper specification limit	3
Mean	1.86136
N	3
SD （Overall）	0.297453
SD （Within group）	0.247916

Mean	1.861
SD	0.2975
N	3
AD	0.323
P value	0.228

Fig 6D p-AKT/AKT

NGF
process report

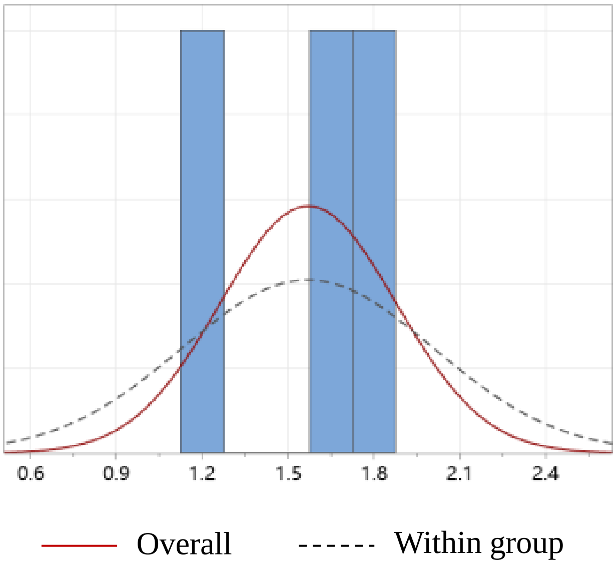

NGF
Normal distribution

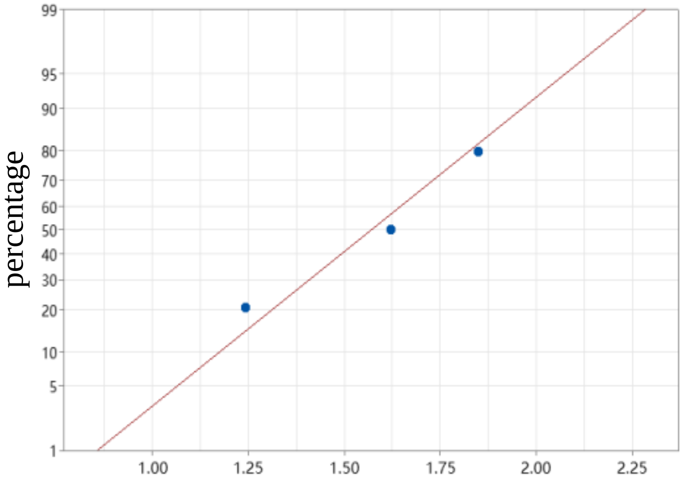

Process data	
Lower Specification Limit	0
Upper specification limit	3
Mean	1.57046
N	3
SD （Overall）	0.307454
SD （Within group）	0.438596

Mean	1.570
SD	0.3075
N	3
AD	0.213
P value	0.535

Fig 6D p-ERK/GAPDH

Control
process report

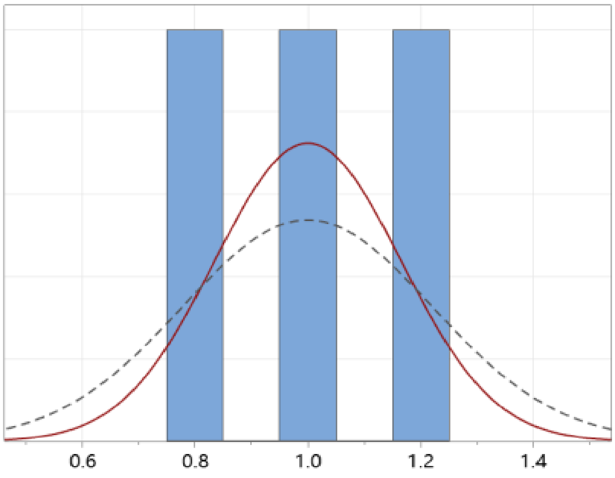

Process data	
Lower Specification Limit	0
Upper specification limit	3
Mean	1
N	3
SD （Overall）	0.165292
SD （Within group）	0.222481

Control
Normal distribution

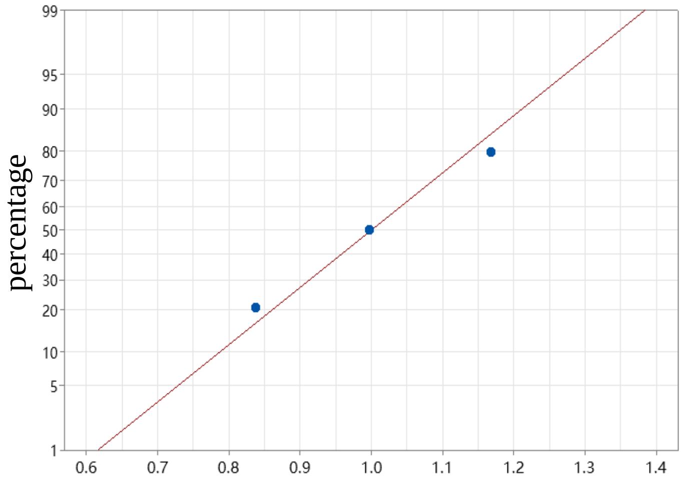

Mean	1
SD	0.1653
N	3
AD	0.190
P value	0.628

Fig 6D p-ERK/GAPDH

Maresin 1
process report

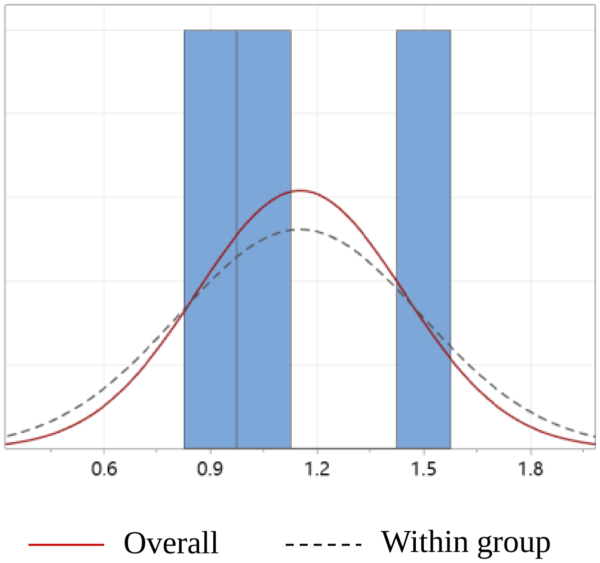

Maresin 1
Normal distribution

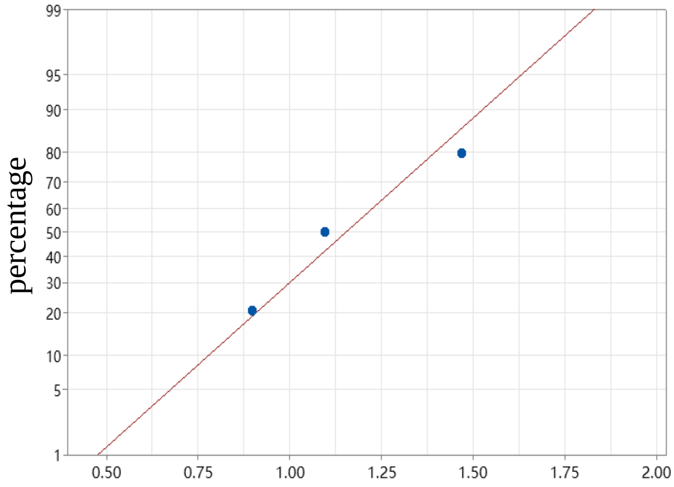

Process data	
Lower Specification Limit	0
Upper specification limit	3
Mean	1.15295
N	3
SD （Overall）	0.291149
SD （Within group）	0.342681

Mean	1.153
SD	0.2911
N	3
AD	0.223
P value	0.508

Fig 6D p-ERK/GAPDH

NGF
process report

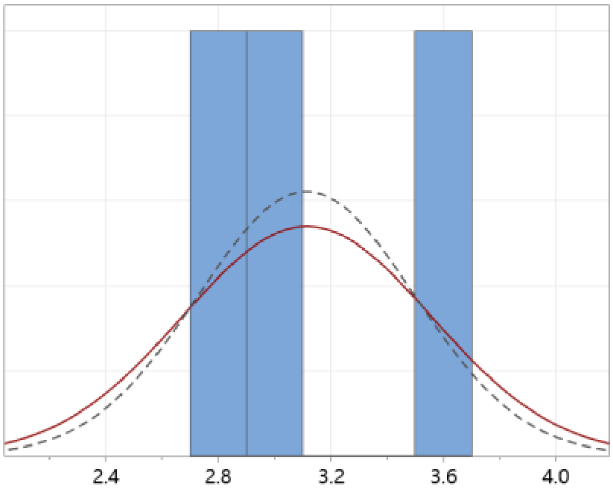

Overall ------ Within group

NGF
Normal distribution

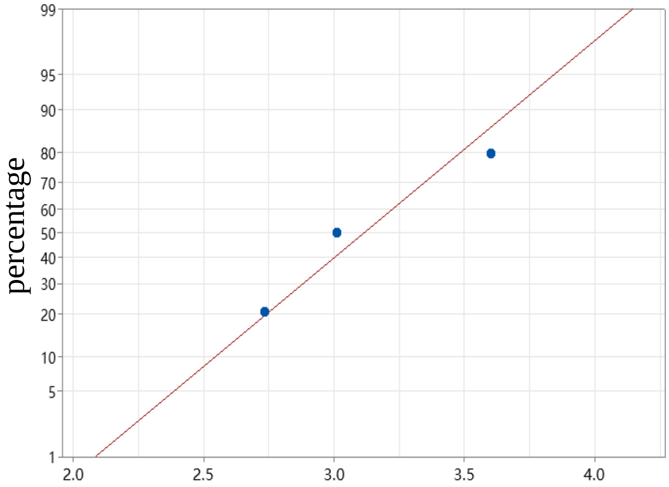

Process data	
Lower Specification Limit	0
Upper specification limit	3
Mean	3.11511
N	3
SD （Overall）	0.443453
SD （Within group）	0.384926

Mean	3.115
SD	0.4435
N	3
AD	0.236
P value	0.463

Fig 6D ERK/GAPDH

Control
process report

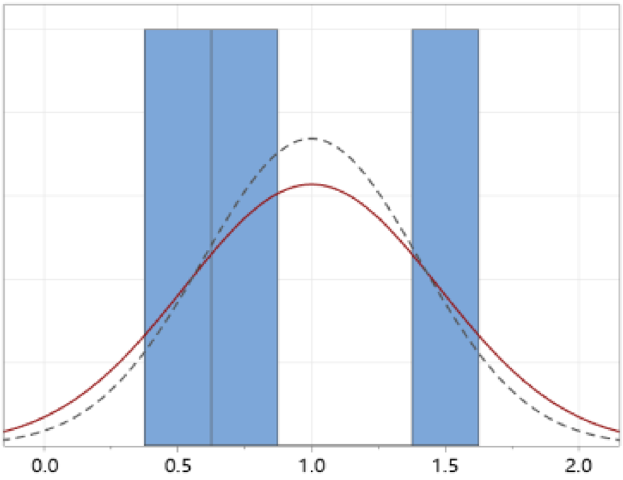

—— Overall ------ Within group

Control
Normal distribution

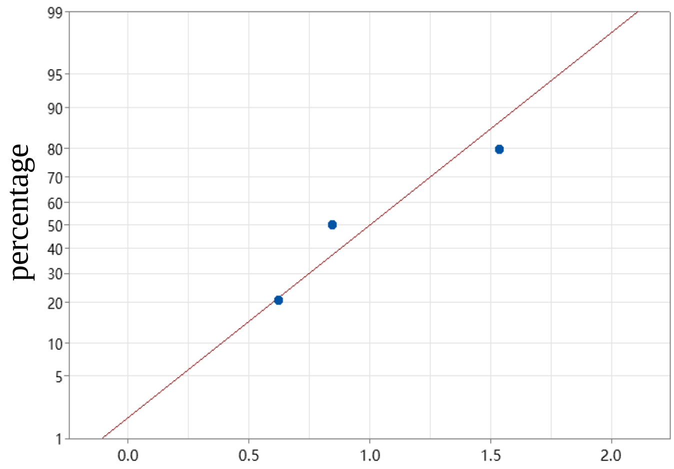

Process data	
Lower Specification Limit	0
Upper specification limit	3
Mean	1
N	3
SD （Overall）	0.476455
SD （Within group）	0.405293

Mean	1
SD	0.4865
N	3
AD	0.280
P value	0.326

Fig 6D ERK/GAPDH

Maresin 1
process report

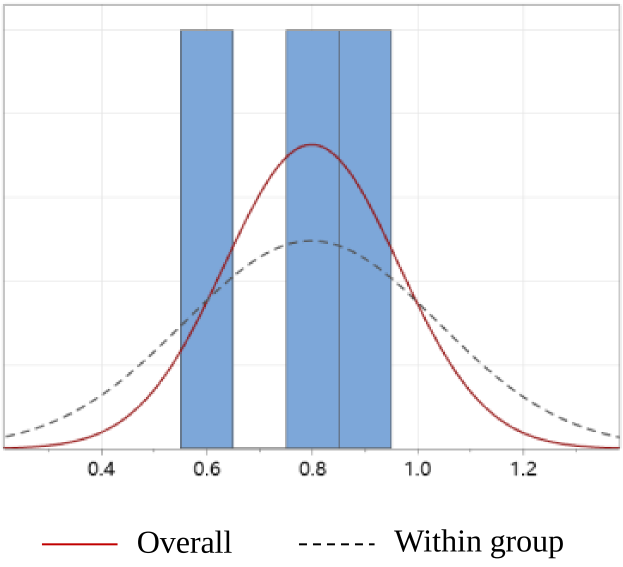

Maresin 1
Normal distribution

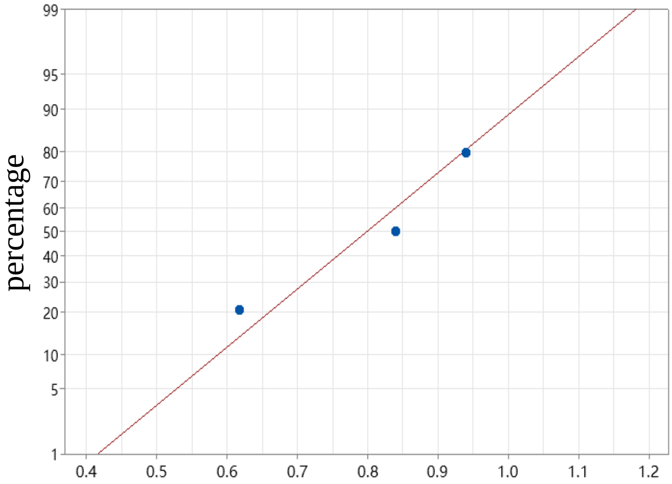

Process data	
Lower Specification Limit	0
Upper specification limit	3
Mean	0.79871
N	3
SD （Overall）	0.164758
SD （Within group）	0.24117

Mean	0.7987
SD	0.1648
N	3
AD	0.242
P value	0.443

Fig 6D ERK/GAPDH

NGF
process report

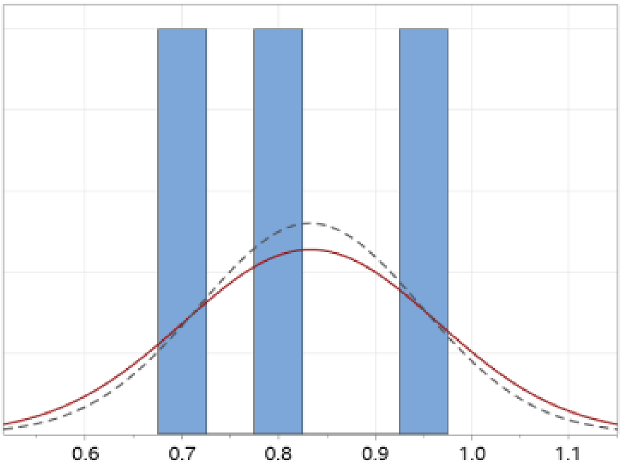

Overall ------ Within group

Process data	
Lower Specification Limit	0
Upper specification limit	3
Mean	0.833094
N	3
SD （Overall）	0.131272
SD （Within group）	0.114989

NGF
Normal distribution

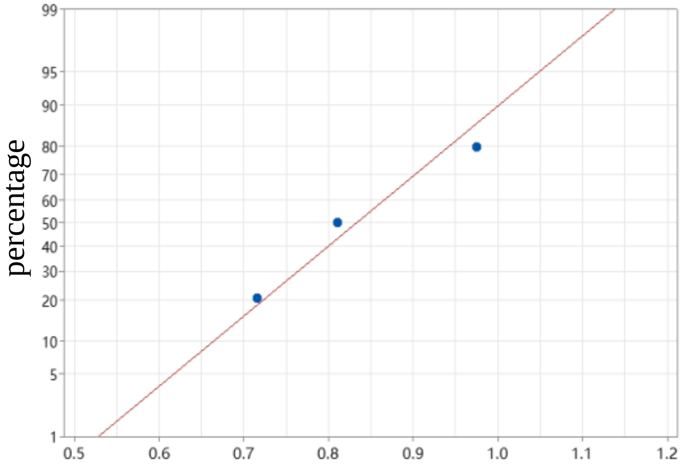

Mean	0.8331
SD	0.1313
N	3
AD	0.216
P value	0.525

Fig 6D p-ERK/ERK

Control
process report

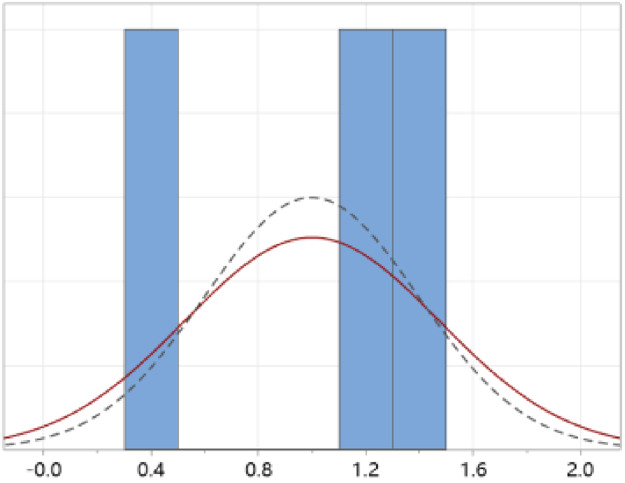

Overall ------ Within group

Control
Normal distribution

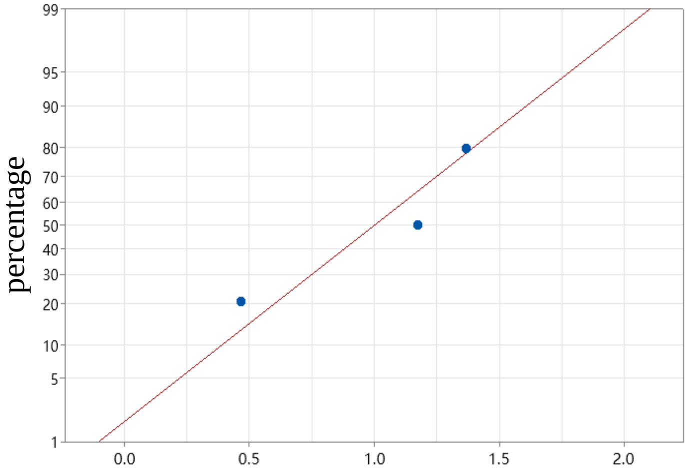

Process data	
Lower Specification Limit	0
Upper specification limit	3
Mean	1
N	3
SD （Overall）	0.474591
SD （Within group）	0.399161

Mean	1
SD	0.4745
N	3
AD	0.304
P value	0.268

Fig 6D p-ERK/ERK

Maresin 1
process report

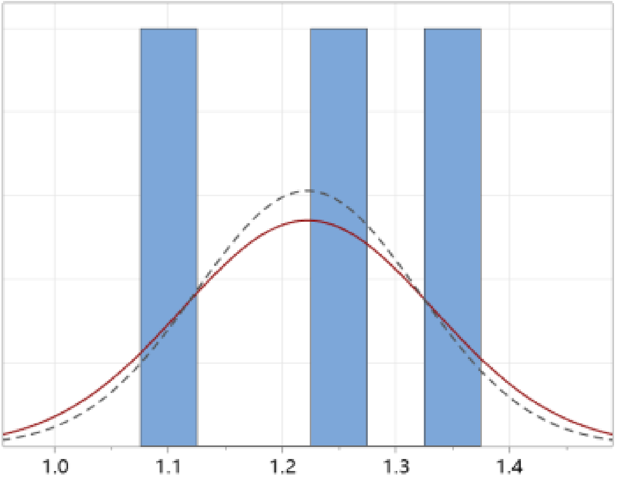

—— Overall ------ Within group

Process data	
Lower Specification Limit	0
Upper specification limit	3
Mean	1.22251
N	3
SD （Overall）	0.110702
SD （Within group）	0.0978861

Maresin 1
Normal distribution

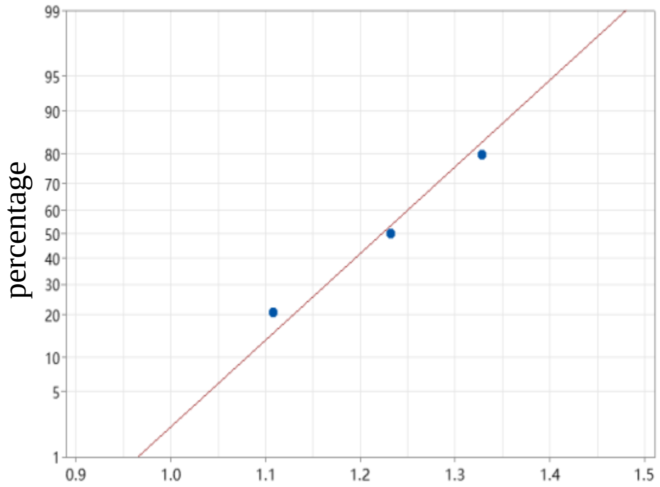

Mean	1.226
SD	0.1107
N	3
AD	0.195
P value	0.604

Fig 6F p-ERK/ERK

NGF
process report

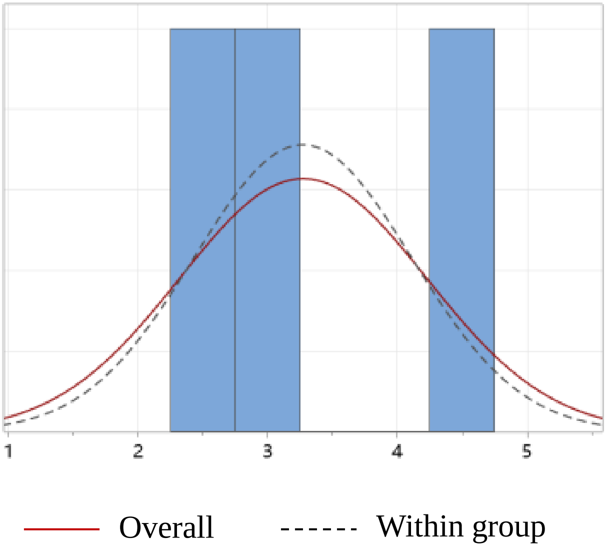

NGF
Normal distribution

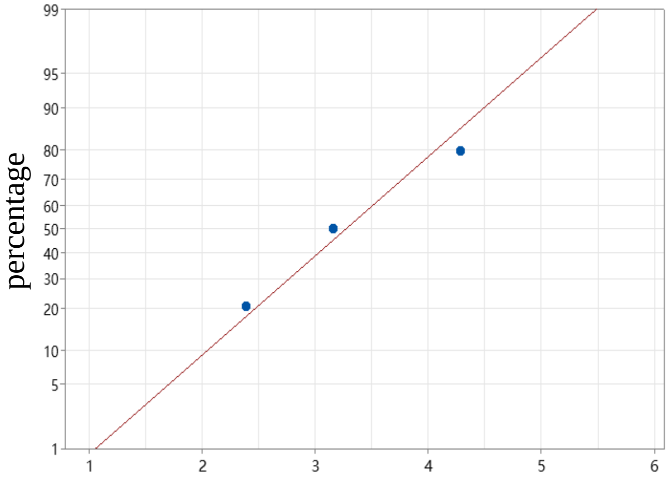

Process data	
Lower Specification Limit	0
Upper specification limit	3
Mean	3.27268
N	3
SD （Overall）	0.953012
SD （Within group）	0.840288

Mean	3.273
SD	0.953
N	3
AD	0.202
P value	0.576

Fig 6F p-mTOR/GAPDH

Control
process report

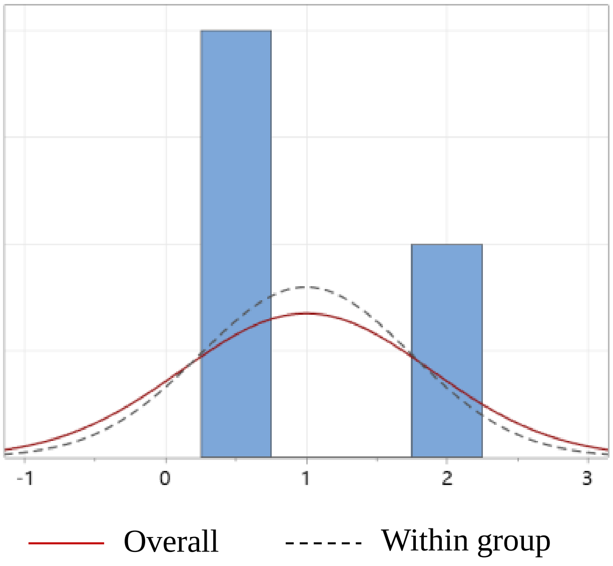

Process data	
Lower Specification Limit	0
Upper specification limit	3
Mean	1
N	3
SD （Overall）	0.882656
SD （Within group）	0.750009

Control
Normal distribution

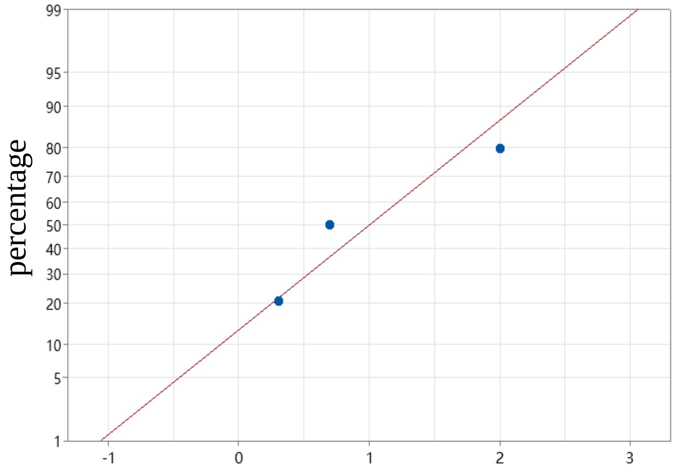

Mean	1
SD	0.8863
N	3
AD	0.291
P value	0.298

Fig 6F p-mTOR/GAPDH

Maresin 1
process report

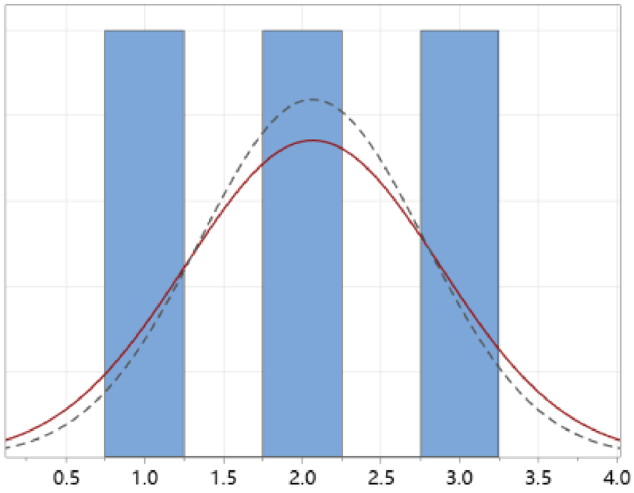

Overall ------ Within group

Maresin 1
Normal distribution

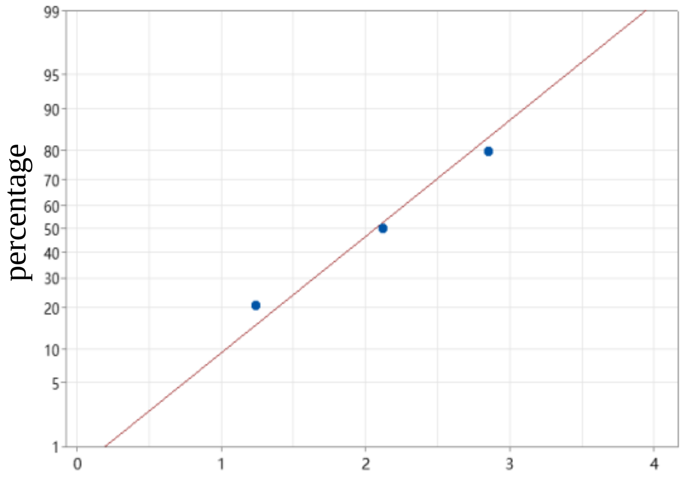

Process data	
Lower Specification Limit	0
Upper specification limit	3
Mean	2.06707
N	3
SD （Overall）	0.805958
SD （Within group）	0.713435

Mean	2.067
SD	0.806
N	3
AD	0.193
P value	0.615

Fig 6F p-mTOR/GAPDH

NGF
process report

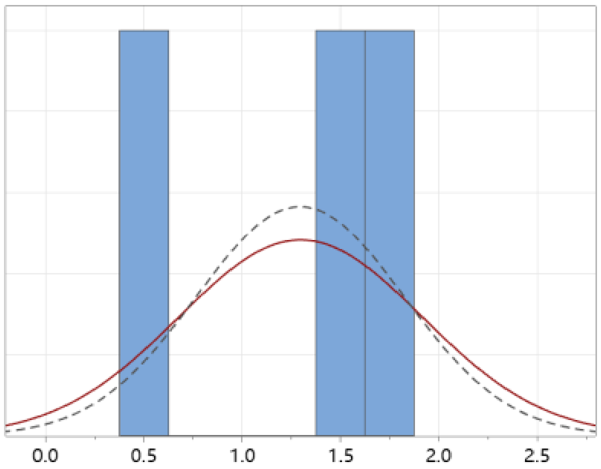

—— Overall ------ Within group

Process data	
Lower Specification Limit	0
Upper specification limit	3
Mean	1.29742
N	3
SD （Overall）	0.619381
SD （Within group）	0.528852

NGF
Normal distribution

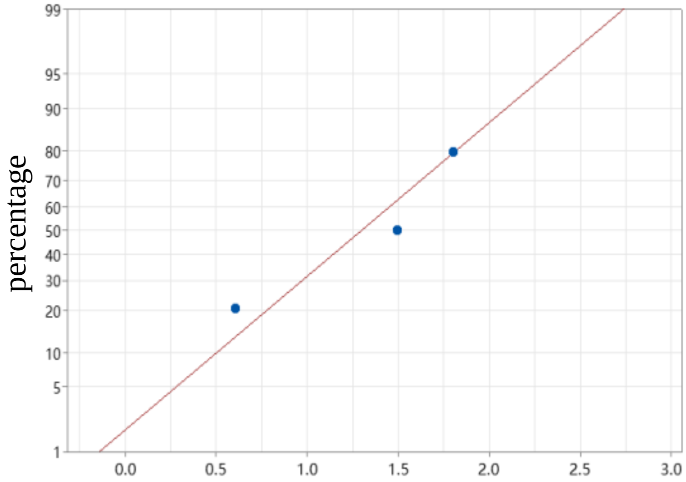

Mean	1.297
SD	0.6194
N	3
AD	0.272
P value	0.348

Fig 6F mTOR/GAPDH

Control
process report

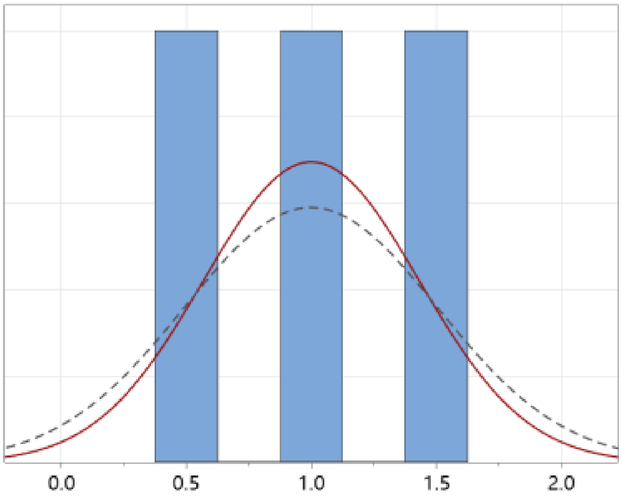

Overall ------ Within group

Control
Normal distribution

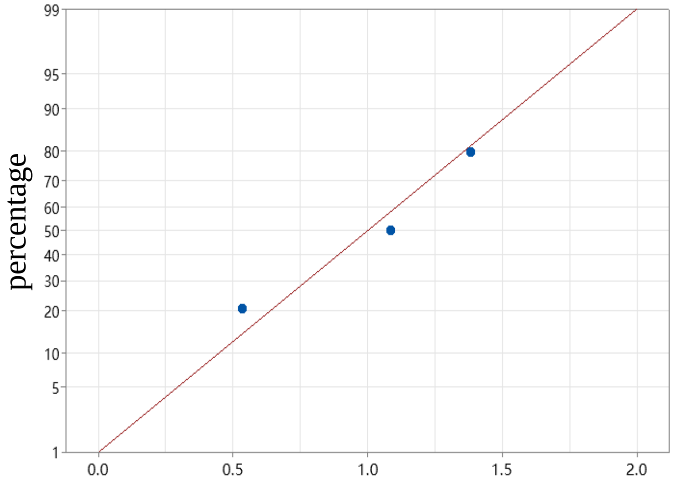

Process data	
Lower Specification Limit	0
Upper specification limit	3
Mean	1
N	3
SD （Overall）	0.429896
SD （Within group）	0.506602

Mean	1
SD	0.4299
N	3
AD	0.223
P value	0.509

Fig 6F mTOR/GAPDH

Maresin 1
process report

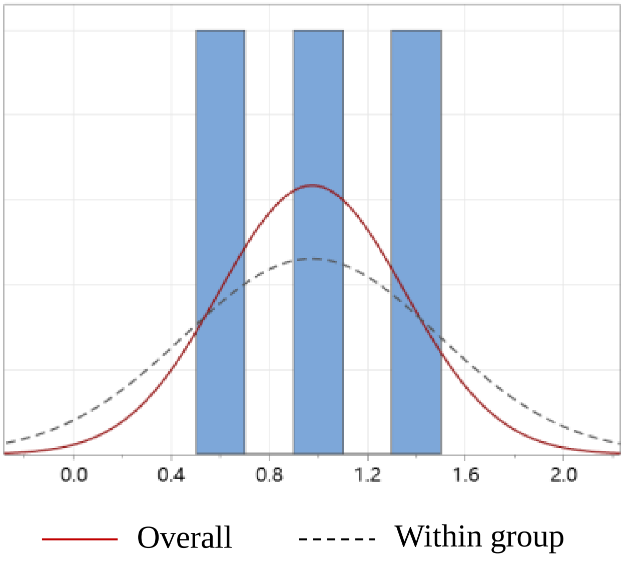

Maresin 1
Normal distribution

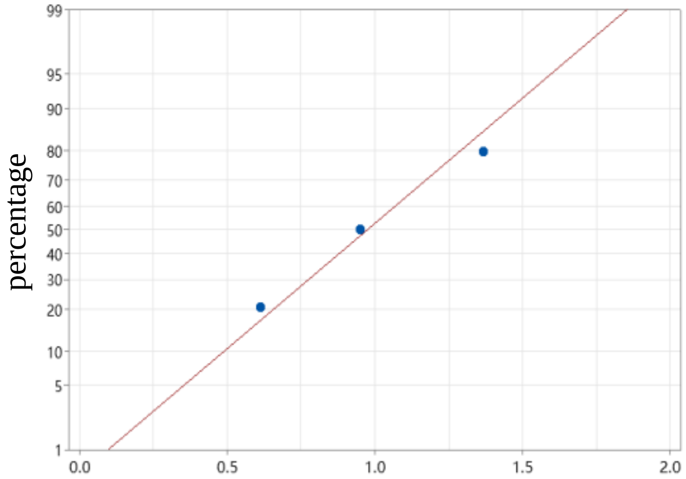

Process data	
Lower Specification Limit	0
Upper specification limit	3
Mean	0.974321
N	3
SD （Overall）	0.377658
SD （Within group）	0.519148

Mean	0.9743
SD	0.3777
N	3
AD	0.194
P value	0.611

Fig 6F mTOR/GAPDH

NGF
process report

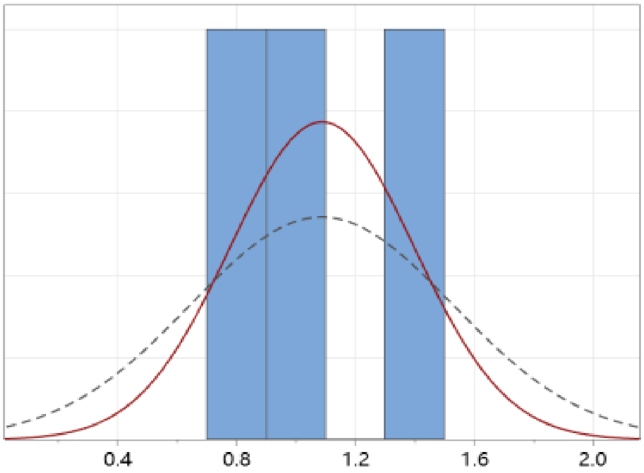

—— Overall ------ Within group

NGF
Normal distribution

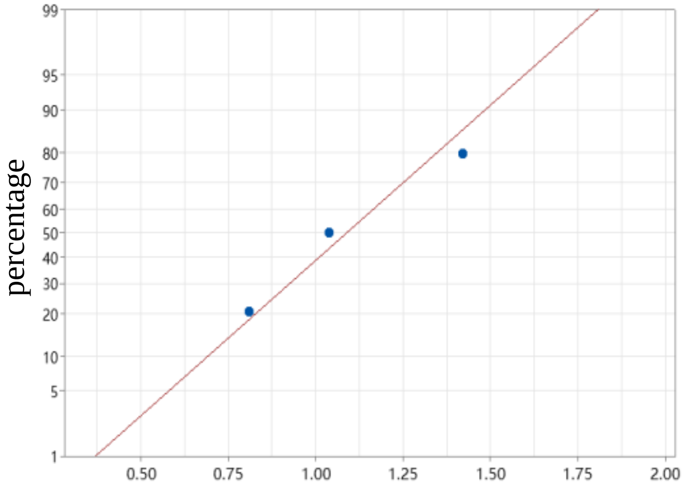

Process data	
Lower Specification Limit	0
Upper specification limit	3
Mean	1.08844
N	3
SD （Overall）	0.309237
SD （Within group）	0.441595

Mean	1.088
SD	0.3092
N	3
AD	0.214
P value	0.532

Fig 6F p-mTOR/mTOR

Control
process report

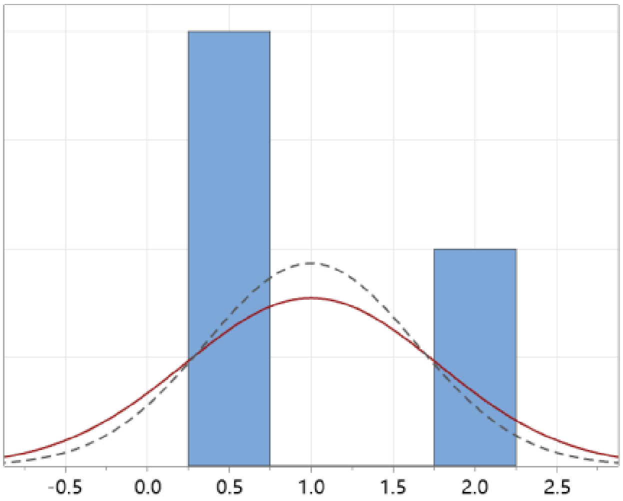

Overall ------ Within group

Control
Normal distribution

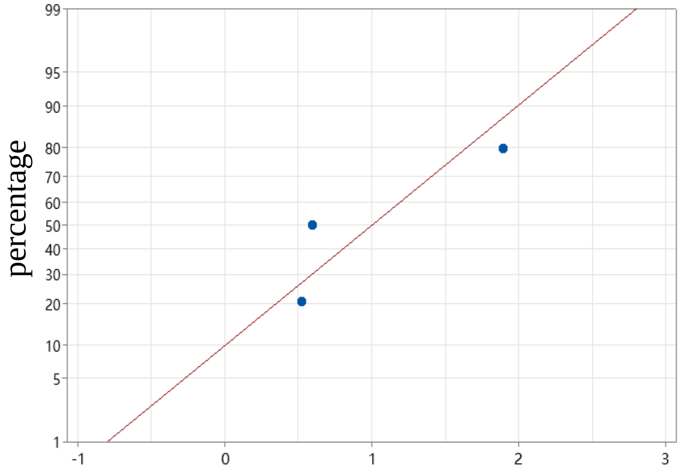

Process data	
Lower Specification Limit	0
Upper specification limit	3
Mean	1
N	3
SD （Overall）	0.774038
SD （Within group）	0.641854

Mean	1
SD	0.7740
N	3
AD	0.438
P value	0.087

Fig 6F p-mTOR/mTOR

Maresin 1
process report

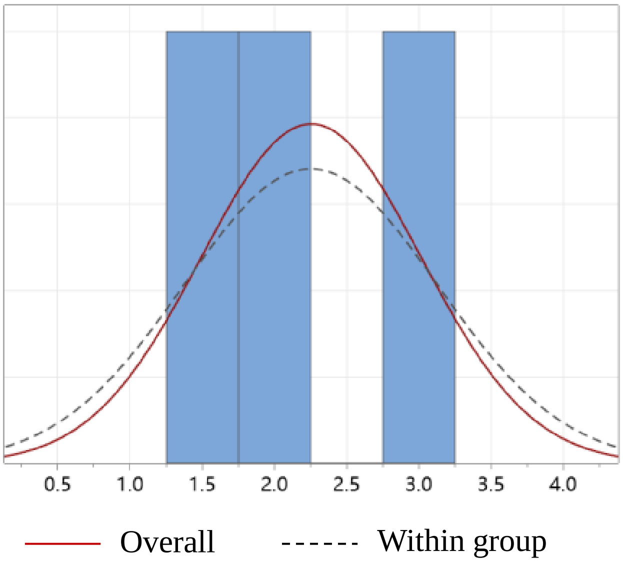

Maresin 1
Normal distribution

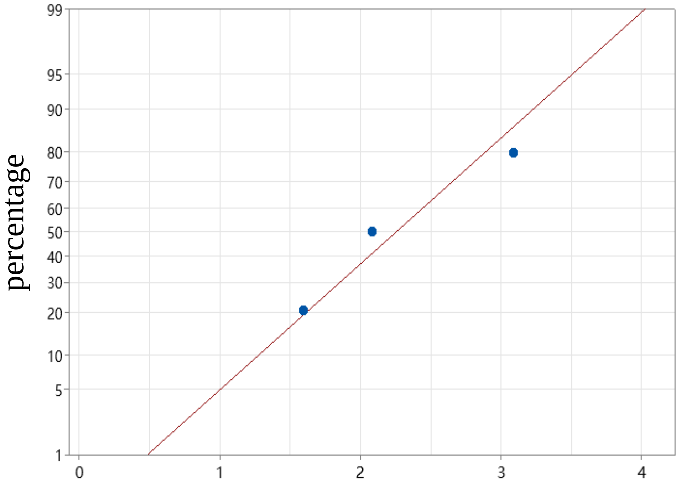

Process data	
Lower Specification Limit	0
Upper specification limit	3
Mean	2.25464
N	3
SD （Overall）	0.76141
SD （Within group）	0.877207

Mean	2.255
SD	0.7614
N	3
AD	0.233
P value	0.473

Fig 6F p-mTOR/mTOR

NGF
process report

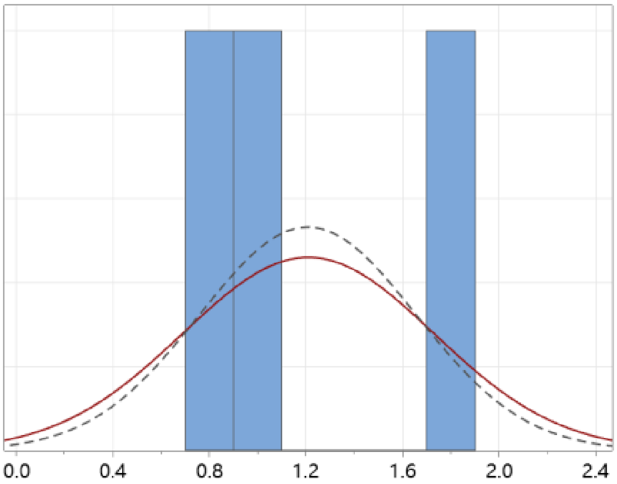

Overall ------ Within group

NGF
Normal distribution

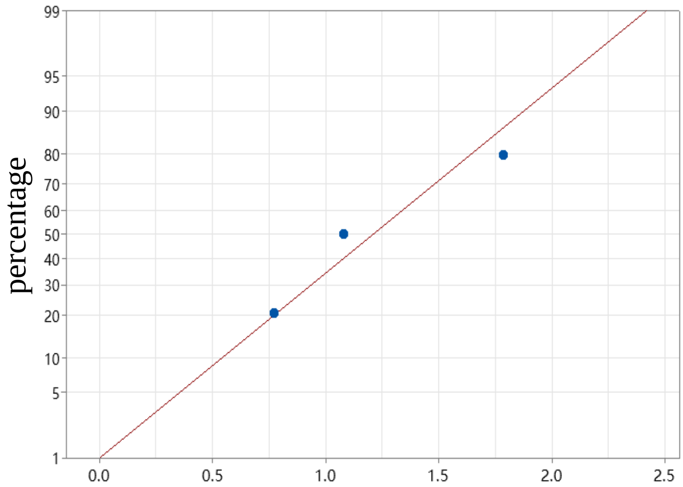

Process data	
Lower Specification Limit	0
Upper specification limit	3
Mean	1.20989
N	3
SD （Overall）	0.520171
SD （Within group）	0.449873

Mean	1.210
SD	0.5202
N	3
AD	0.244
P value	0.435

Fig 6F p-PI3K/GAPDH

Control
process report

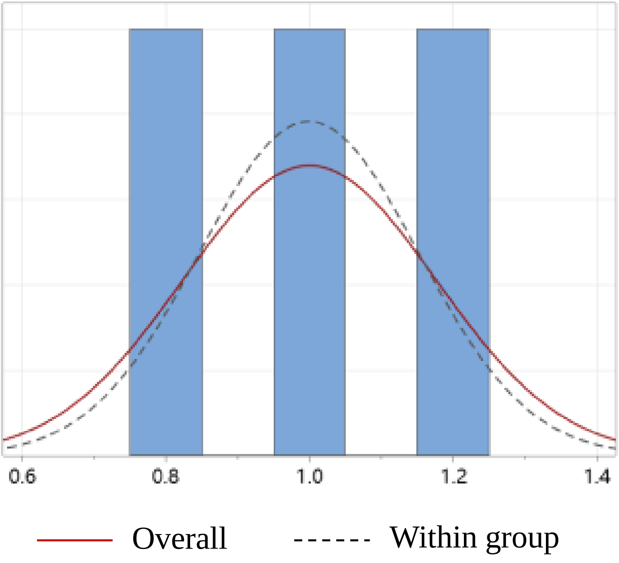

Control
Normal distribution

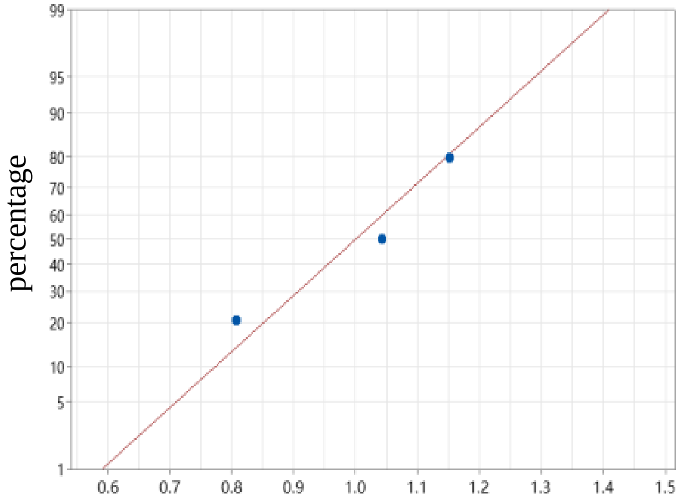

Process data	
Lower Specification Limit	0
Upper specification limit	3
Mean	1
N	3
SD （Overall）	0.170618
SD （Within group）	0.1527

Mean	1
SD	0.176
N	3
AD	0.237
P value	0.458

Fig 6F p-PI3K/GAPDH

Maresin 1
process report

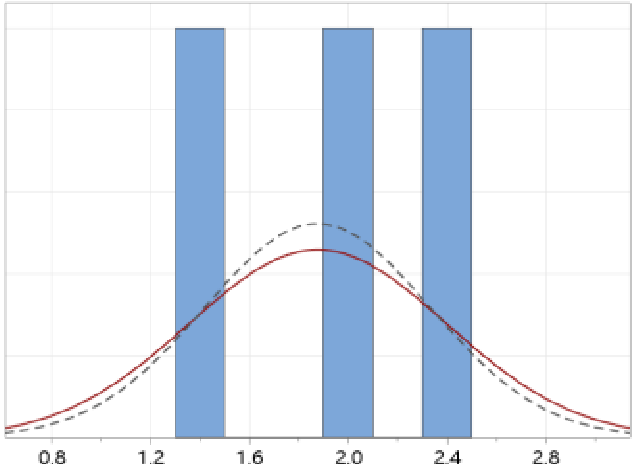

Maresin 1
Normal distribution

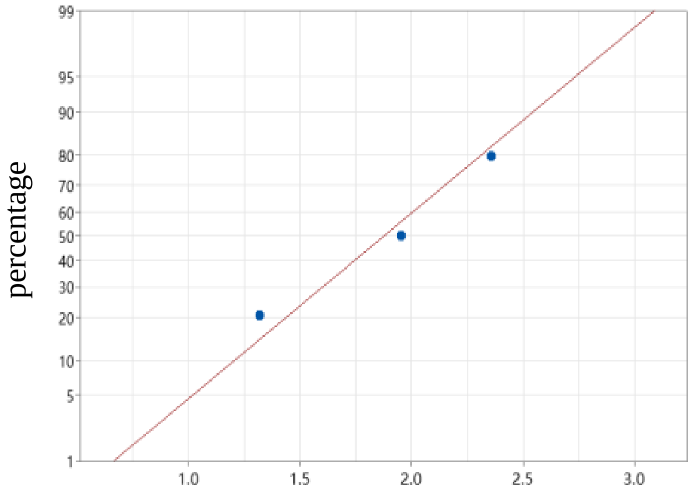

Process data	
Lower Specification Limit	0
Upper specification limit	3
Mean	1.87655
N	3
SD （Overall）	0.521674
SD （Within group）	0.458597

Mean	1.877
SD	0.5217
N	3
AD	0.208
P value	0.550

Fig 6F p-PI3K/GAPDH

NGF
process report

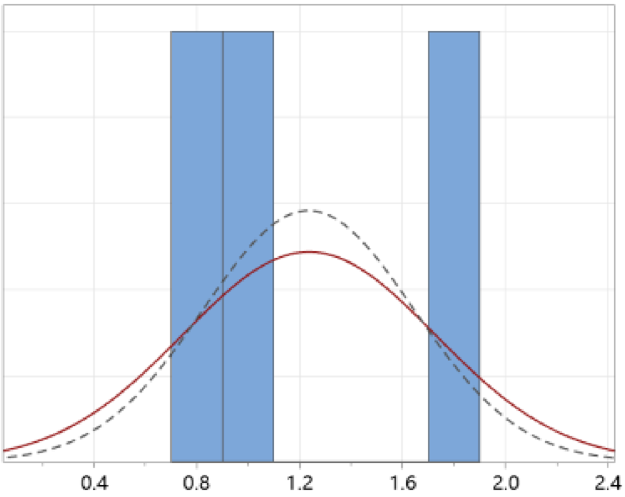

Overall ------ Within group

NGF
Normal distribution

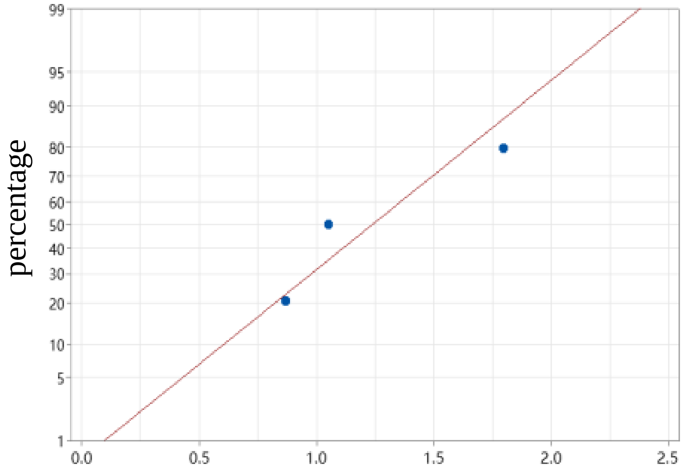

Process data	
Lower Specification Limit	0
Upper specification limit	3
Mean	1.23649
N	3
SD （Overall）	0.49094
SD （Within group）	0.410258

Mean	1.266
SD	0.4909
N	3
AD	0.317
P value	0.239

Fig 6F PI3K/GAPDH

Control
process report

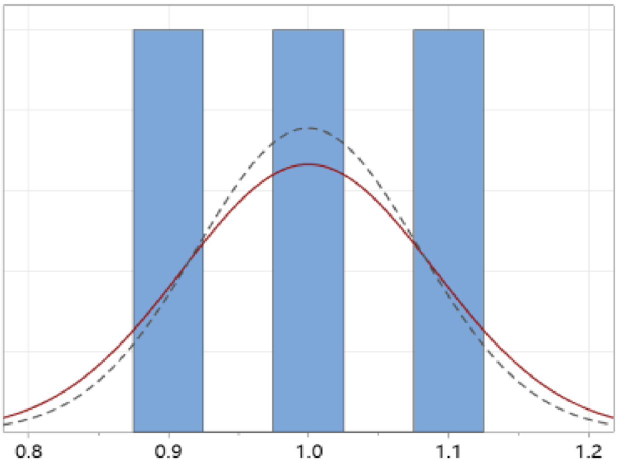

Overall ------ Within group

Control
Normal distribution

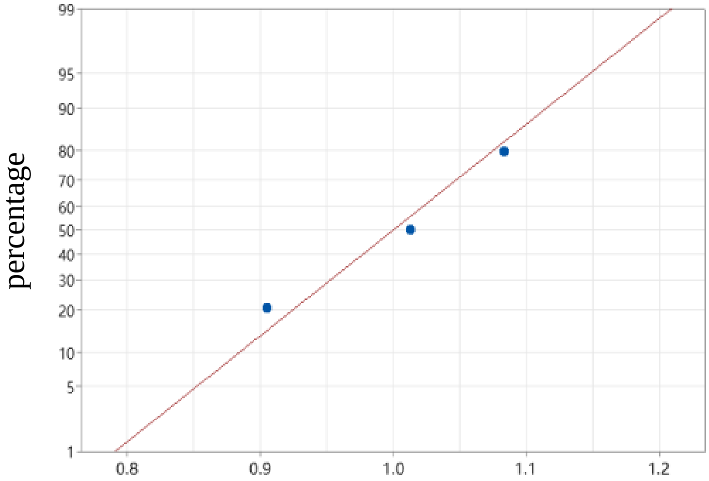

Process data	
Lower Specification Limit	0
Upper specification limit	3
Mean	1
N	3
SD （Overall）	0.0899645
SD （Within group）	0.0792083

Mean	1
SD	0.08996
N	3
AD	0.205
P value	0.563

Fig 6F PI3K/GAPDH

Maresin 1
process report

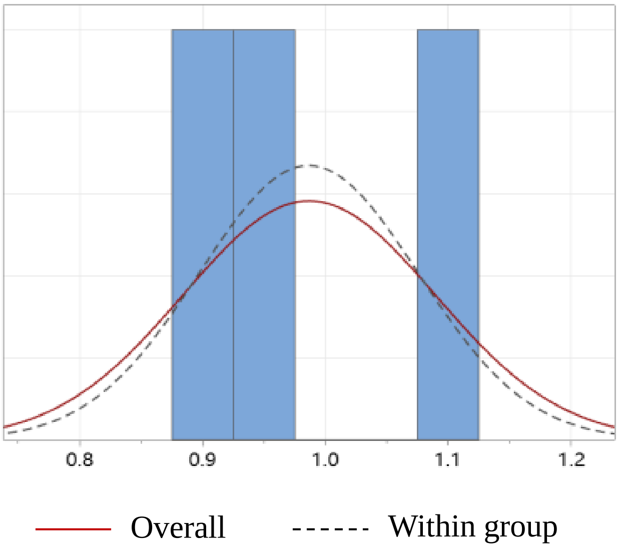

Maresin 1
Normal distribution

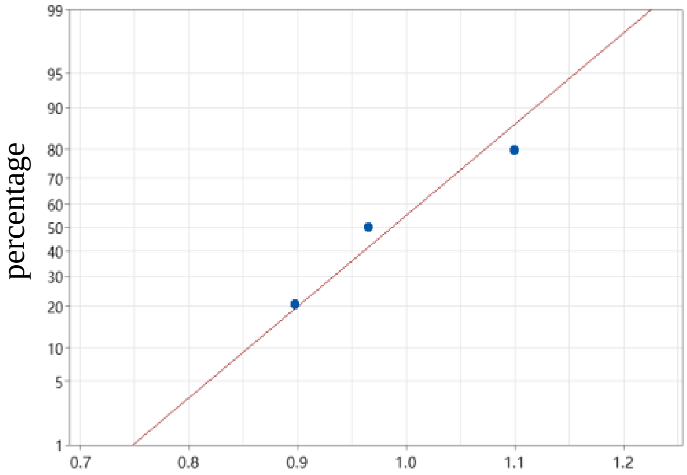

Process data	
Lower Specification Limit	0
Upper specification limit	3
Mean	0.986831
N	3
SD （Overall）	0.102702
SD （Within group）	0.084473

Mean	0.9868
SD	0.1027
N	3
AD	0.229
P value	0.491

Fig 6F PI3K/GAPDH

NGF
process report

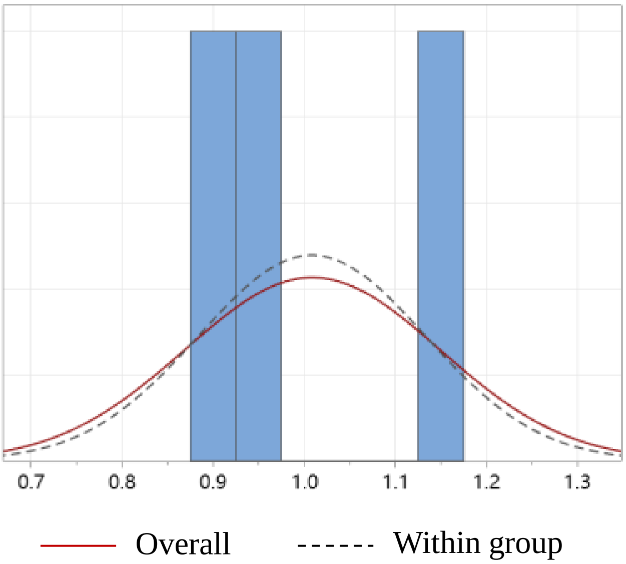

NGF
Normal distribution

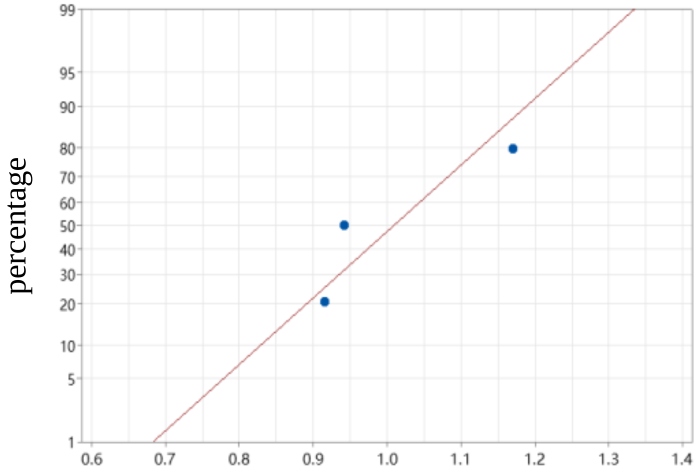

Process data	
Lower Specification Limit	0
Upper specification limit	3
Mean	1.00879
N	3
SD （Overall）	0.140144
SD （Within group）	0.125043

Mean	1.009
SD	0.1401
N	3
AD	0.390
P value	0.128

Fig 6F p-PI3K/PI3K

Control
process report

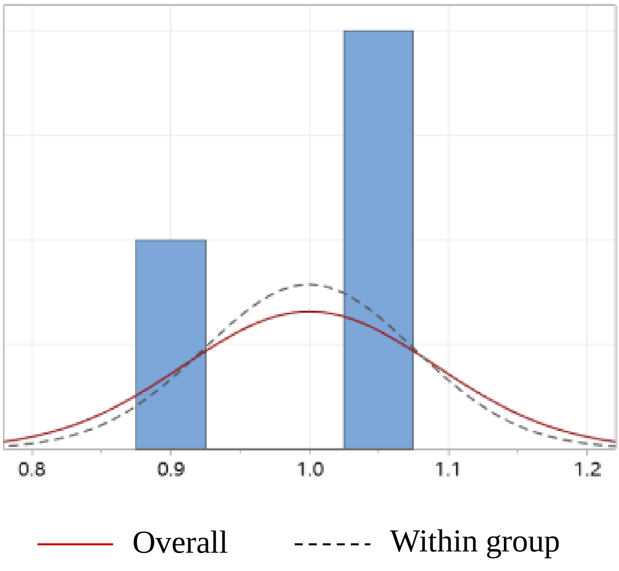

Control
Normal distribution

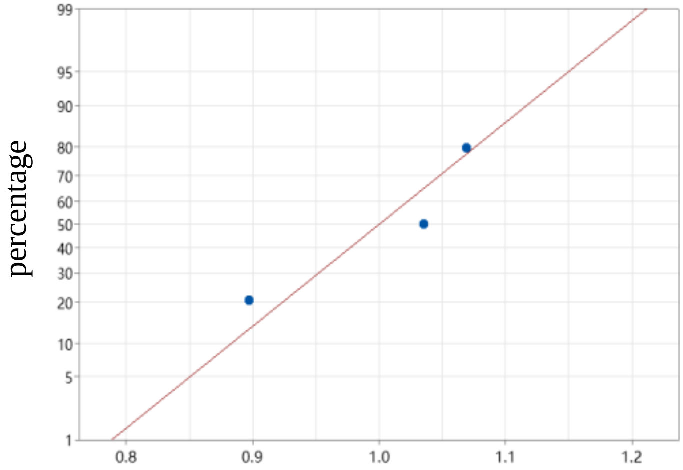

Process data	
Lower Specification Limit	0
Upper specification limit	3
Mean	1
N	3
SD （Overall）	0.0910364
SD （Within group）	0.0761467

Mean	1
SD	0.09104
N	3
AD	0.315
P value	0.243

Fig 6F p-PI3K/PI3K

Maresin 1
process report

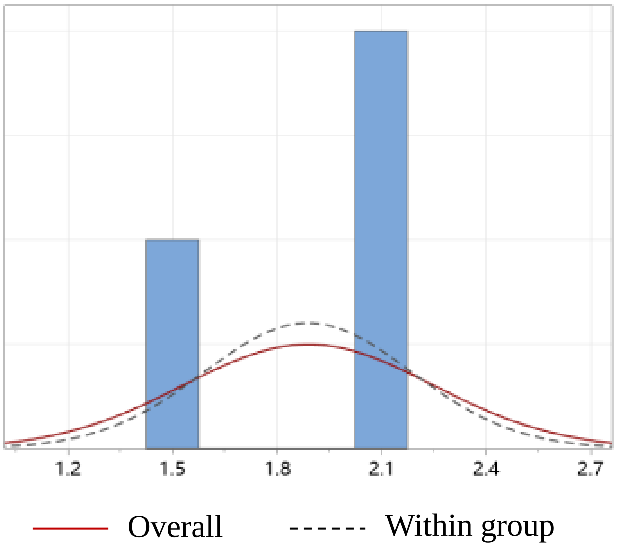

Maresin 1
Normal distribution

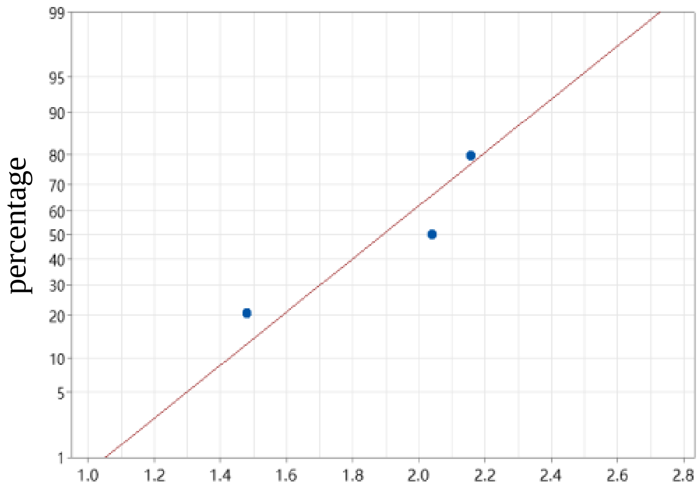

Process data	
Lower Specification Limit	0
Upper specification limit	3
Mean	1.89037
N	3
SD （Overall）	0.360439
SD （Within group）	0.299126

Mean	1.890
SD	0.3604
N	3
AD	0.332
P value	0.211

Fig 6F p-PI3K/PI3K

NGF
process report

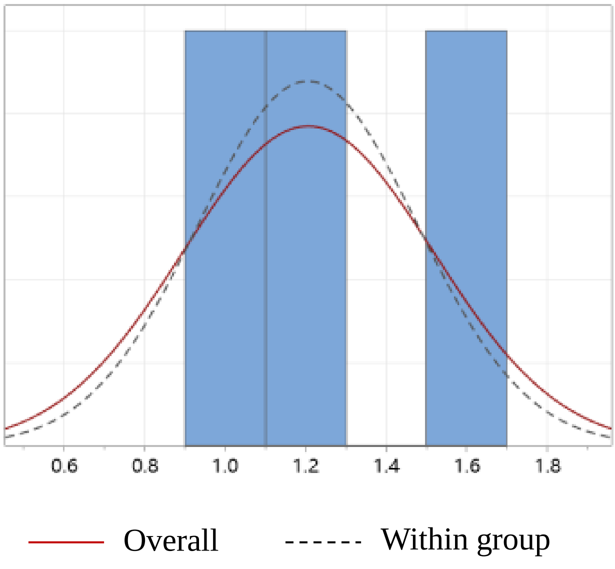

NGF
Normal distribution

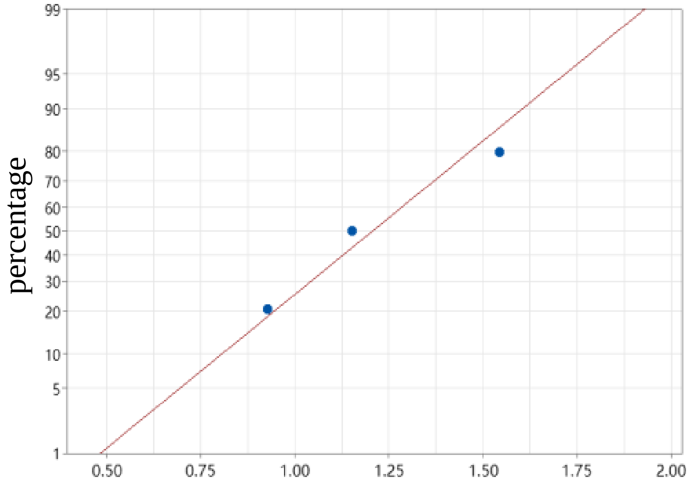

Process data	
Lower Specification Limit	0
Upper specification limit	3
Mean	1.20649
N	3
SD （Overall）	0.311057
SD （Within group）	0.272438

Mean	1.206
SD	0.3111
N	3
AD	0.216
P value	0.524
